# Identification and analyses of the chemical composition of a naturally occurring albino mutant chanterelle

**DOI:** 10.1038/s41598-021-99787-8

**Published:** 2021-10-18

**Authors:** R. Greg Thorn, Alicia Banwell, Thu Huong Pham, Natalia P. Vidal, Charles Felix Manful, Muhammad Nadeem, Alexander G. Ivanov, Beth Szyszka Mroz, Michael B. Bonneville, Norman Peter Andrew Hüner, Michele D. Piercey-Normore, Raymond Thomas

**Affiliations:** 1 Department of Biology, University of Western Ontario, 1151 Richmond St. N., London, ON N6A 5B7 Canada; 2 School of Science and the Environment, Grenfell Campus, Memorial University, 20 University Drive, Corner Brook, NL A2H 5G4 Canada; 3 Institute of Biophysics and Biomedical Engineering, Bulgarian Academy of Sciences, Acad. G. Bonchev str. Bl. 21, 1113 Sofia, Bulgaria; 4 Present Address: Department of Food Science, iFOOD Multidisciplinary Center, Aarhus University, Agro Food Park 48, 8200 Aarhus N, Denmark

**Keywords:** Biochemistry, Ecology, Evolution, Microbiology, Ecology

## Abstract

White chanterelles (Basidiomycota), lacking the orange pigments and apricot-like odour of typical chanterelles, were found recently in the Canadian provinces of Québec (QC) and Newfoundland & Labrador (NL). Our phylogenetic analyses confirmed the identification of all white chanterelles from NL and QC as *Cantharellus enelensis*; we name these forma *acolodorus*. We characterized carotenoid pigments, lipids, phenolics, and volatile compounds in these and related chanterelles. White mutants of *C. enelensis* lacked detectable β-carotene, confirmed to be the primary pigment of wild-type, golden-orange individuals, and could also be distinguished by their profiles of fatty acids and phenolic acids, and by the ketone and terpene composition of their volatiles. We detected single base substitutions in the phytoene desaturase (*Al-1*) and phytoene synthase (*Al-2*) genes of the white mutant, which are predicted to result in altered amino acids in their gene products and may be responsible for the loss of β-carotene synthesis in that form.

## Introduction

**Figure 1:**
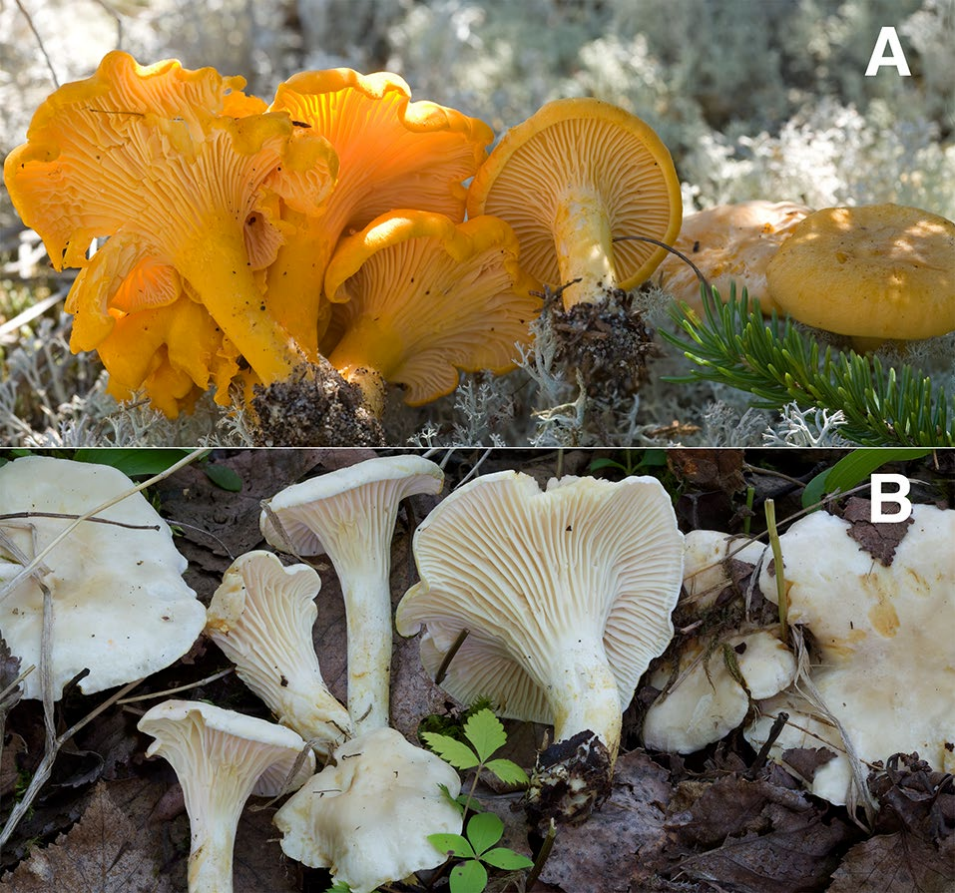
Typical and albino forms of *Cantharellus enelensis*. (**A**) Typical form, with golden-orange colouration (henceforth referred to as gold or golden). (**B**) The albino form, *C. enelensis* f. *acolodorus* (17.08.15.av01; NLW3 in chemical analyses), showing buffy yellow staining in age or on handling. When dried, the two forms are morphologically indistinguishable. Photos: Andrus Voitk.

Chanterelles (*Cantharellus*: Basidiomycota) are widely distributed and highly prized edible mushrooms with an estimated annual international export market of over $1.5 billion US^1,2^. Chanterelles are ectomycorrhizal, growing in a mutualistic association with host trees, and thus cannot be cultivated readily for commercial sale but are wild-harvested in the forest by both amateur enthusiasts and commercial mushroom pickers^3–6^. A large part of the culinary appeal of chanterelles is their brilliant golden-orange colour (Fig. 1A), their apricot-like odour, and firm texture. Chanterelles are famous for their long-lasting fruiting bodies, which can persist in the woods in good condition for weeks or months, often without undergoing decay or being consumed by slugs, fly larvae, or other invertebrates^4,7^. The chemical components that prevent microbial decay or invertebrate consumption are largely unknown, but have been suggested to be associated with the colour and odour^4^.

Until recently, most golden-orange chanterelles were referred to as *Cantharellus cibarius* Fr., and this name is still widely used in commerce and by mushroom enthusiasts. Studies using DNA sequence data have shown that *C. cibarius* is restricted to Eurasia and have delimited multiple species of golden-orange chanterelles around the world^8^. In the Canadian province of Newfoundland and Labrador (NL), the common species of golden-orange chanterelles was recently described as *Cantharellus enelensis*, and differentiated from two other, less common golden species *C. camphoratus* and *C. amethysteus*^9^, the latter now separated as a new species *C. betularum*^10^. Soon after that publication, scattered fruitings of pure white chanterelles were reported across the island of Newfoundland (Fig. 1B), often occurring mixed within normally pigmented individuals of *C. enelensis*^11^. Fruiting bodies of the white chanterelles differed not only in colour but in the absence of the apricot-like odour of the typical golden-orange specimens^11^. While investigating the NL white chanterelles, we were sent white chanterelles from Québec (QC), Canada, and specimens of commercially harvested pale chanterelles from Minnesota (MN) in the USA.

Golden chanterelles get their colour from carotenoid pigments^12,13^. Carotenoid analyses have been performed on *C. cibarius*, but not extensively studied because carotenoids can be difficult to analyze since they degrade over time and rapidly with drying^14^. The principal carotenoid in *C. cibarius* is β-carotene, which is responsible for its golden appearance, followed by lycopene, as well as some α-carotene and γ-carotene^13,15,16^. Carotenoid synthesis has been studied in the filamentous ascomycete *Neurospora crassa*, and the genes producing the key enzymes have been named for the albino phenotype of their mutant alleles. The 20-carbon precursor is formed by geranylgeranyl pyrophosphate synthetase, encoded by the gene referred to as albino-3 (*Al-3*). Dimerization to form the 40-carbon colourless phytoene is carried out by the phytoene synthase activity of the dual-function gene product of albino-2 (*Al-2*). Phytoene desaturase (encoded by albino-1, or *Al-1*) converts phytoene to lycopene through a series of cyclic reactions, which is then converted to the coloured product β-carotene by the lycopene cyclase function of *Al-2*^17^.

Our first objective was to determine the species identity of the white and pale chanterelles from NL, QC and MN using phylogenetic analyses of nuclear ribosomal DNA (internal transcribed spacer, or ITS, and large subunit, LSU) and the translation elongation factor gene (*Tef-1*). The presence of a pigmentless chanterelle with altered odour profile raised the questions of the genetic underpinning of the apparent albinism, of how the chemical composition of these variants compared to typical golden-orange specimens of *C. enelensis, C. camphoratus*, and *C. betularum*, and whether specific metabolites could be used to differentiate and identify species and colour variants of chanterelles.

## Results

**Figure 2:**
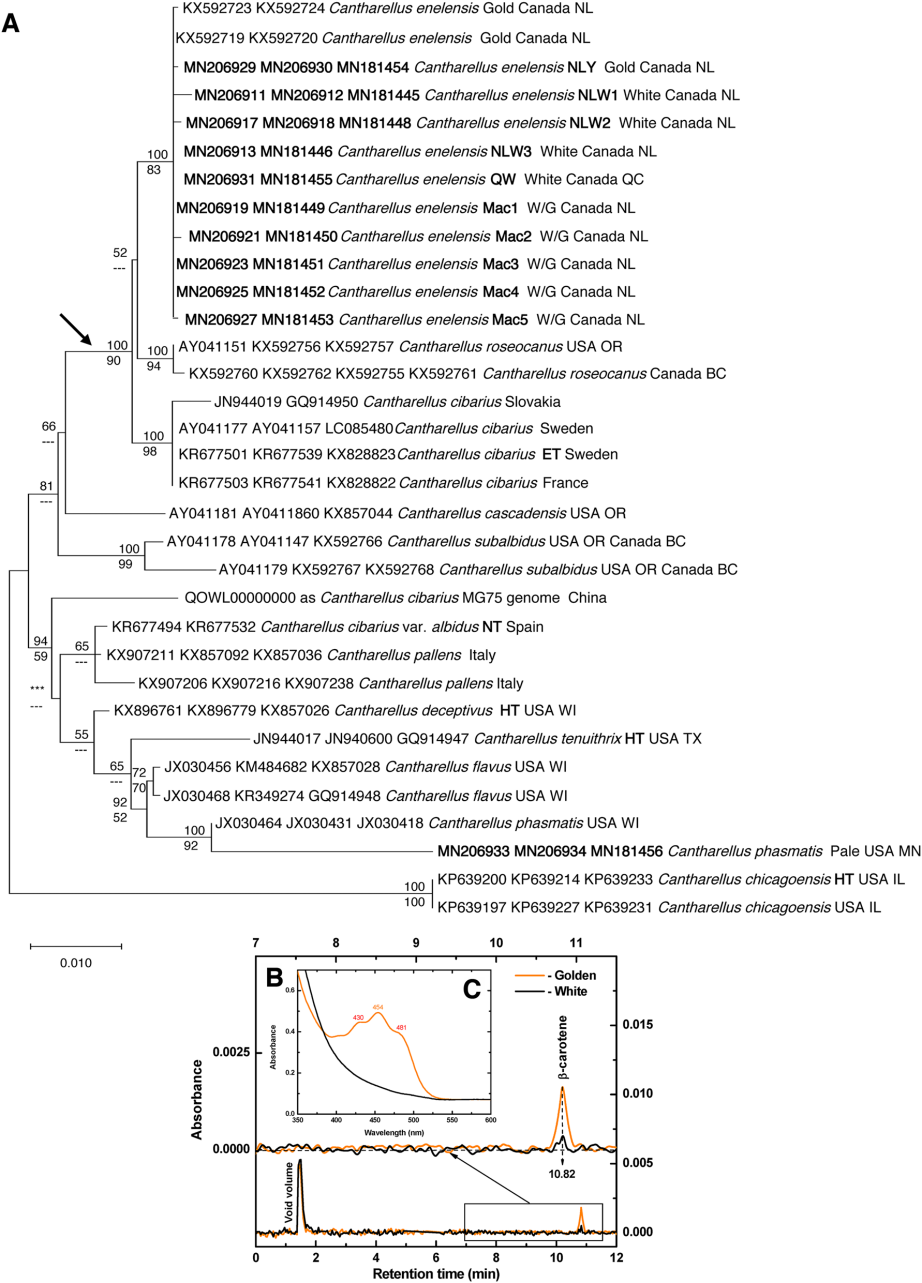
White﻿ chanterelles from Newfoundland and Québec are members of the species *Cantharellus enelensis* and are distinguished by their lack of β-carotene. (**A**) Maximum likelihood phylogeny of white and golden representatives of *Cantharellus enelensis*, related species of the core *C. cibarius* clade (arrow) and its sister group, the clade including *C. pallens* through *C. phasmatis*, rooted with *C. chicagoensis*. The tree is based on sequences from nuclear ribosomal internal transcribed spacer (ITS), large subunit (LSU) and translation elongation factor 1-alpha (Tef1) regions. All sequences are identified by the GenBank accession numbers and the name they were deposited under, and new sequences obtained in this study are indicated in bold font. Sequences from type specimens are indicated with HT (holotype), NT (neotype) or ET (epitype). Table 1 provides collection details for samples used in chemical analyses; their specimen codes used in Fig. 3 are included in bold following the species name in this tree. Node support values (%) are provided from a Bayesian inference analysis (posterior probabilities, above nodes) and a 1000× maximum likelihood bootstrap analysis (below nodes). Nodes with less than 50% support are shown by dashes, and a single node that collapsed in Bayesian analysis is shown by asterisks. (**B,C**) Representative HPLC chromatograms and absorbance spectra of pigments extracted from white and golden variants of *Cantharellus enelensis*. (**B**) Representative HPLC chromatograms, the upper traces showing an enlargement of the area of interest. (**C**) Absorbance spectra of acetone extracts.

New ITS, LSU and *Tef-1* sequences were generated from fifteen specimens in this study (Table 1) and were aligned with sequences downloaded from GenBank. The maximum likelihood tree produced in MEGA X, with node support from 1000× bootstrap replicates and from Bayesian posterior probabilities of an analysis with 5 million trees in MrBayes is shown (Fig. 2A). White chanterelles from NL and QC cluster phylogenetically with the NL golden species, *C. enelensis,* with strong bootstrap support and well separated from *C. roseocanus*, a golden species from the Pacific Coast of North America, *C. cibarius*, a golden species from Europe and *C. cascadensis,* a golden to white species found on the Pacific Coast of North America. We provide a name for this white variant at the rank of “forma”.

**Table 1:** *Cantharellus* specimens used for chemical analyses, their colour, source, preservation conditions, and GenBank accession number of reference ITS sequence.

Identification	Colour, code	Source, collection no. (Herbarium accession #s)	Preservation	GenBank No
*C. betularum* (formerly identified as *C. amethysteus*)	Golden, AY	Humber Village, NL, M. Voitk, 17.09.30.av01 (UWO-F326, DAOM 984767)	Dried	MN206940
*C. camphoratus*	Golden, CY	Deer Lake, NL, H. Mann, 17.10.22.av02 (DAOM 984889)	Dried	ND^a^
*C. enelensis*	Golden, NLY	Gambo, NL, A. Voitk, 17.08.15.av02 (UWO-F704, DAOM 984887)	Dried	MN206930
*C. enelensis*	Golden (pigment analysis)	Avalon Peninsula, NL, S. Dawson, RGT 190913/02 (UWO-F705, DAOM)	Frozen	ND
*C. enelensis*	White (pigment analysis)	Avalon Peninsula, NL, S. Dawson, RGT 190913/01 (UWO-F202, DAOM 970946)	Frozen	ND
*C. enelensis*	White, NLW1	Gambo, NL, E. Kean, 17.08.11.av06 (UWO-F703, DAOM 984884)	Dried	MN206912
*C. enelensis*	White, NLW2	St. John’s, NL, D. Sparks, 17.08.13.av01 (UWO-F706, DAOM 984885)	Dried	MN206917
*C. enelensis*	White, NLW3	Gambo, NL, B. Bryden, 17.08.15.av01 (UWO-F707, DAOM 984886)	Dried	MN206913
*C. enelensis*	White, QW	St. Alban, QC, R. Lebeuf, HRL 2585 (UWO-F708, DAOM 984888)	Dried	MN206931
*C. enelensis*	White/golden, Mac1	Brigus Junction, NL, M. Pitcher 1 (UWO-F709)	Dried	MN206919
*C. enelensis*	White/golden, Mac2	Brigus Junction, NL, M. Pitcher 2 (UWO-F710)	Dried	MN206921
*C. enelensis*	White/golden, Mac3	Brigus Junction, NL, M. Pitcher 3 (UWO-F711)	Dried	MN206923
*C. enelensis*	White/golden, Mac4	Brigus Junction, NL, M. Pitcher 4 (UWO-F712)	Dried	MN206925
*C. enelensis*	White/golden, Mac5	Brigus Junction, NL, M. Pitcher 5 (UWO-F713)	Dried	MN206927

Specimens labelled white/golden are white or golden individuals from a mixed collection of both white and golden chanterelles that were indistinguishable after desiccation.

*NL* Newfoundland & Labrador, *QC* Québec.

^a^18.09.13.av07 (UWO-F703), from same population, MN206937.

***Cantharellus enelensis* f*. acolodorus***, Voitk & Thorn, forma nova. Figure 1B MycoBank MB835379.

### Typification

CANADA. NEWFOUNDLAND AND LABRADOR: Gambo, Mint Forest resource road, in spruce forest mixed with birch, among moss and duff (48° 43′ 02.3′′ N, 54° 34′ 56.5′′ W; 143 m above sea level), 11 Aug 2017, Eugene Kean, A. Voitk coll. no. 17.08.11.av06 (holotype UWO-F730, isotype TU117603). GenBank: ITS = MN206912.

### Etymology

Acolodorus is a contracted combination adjective from Latin (a = none), to indicate without colour or odour.

### Diagnosis

A white chanterelle found among golden specimens of *C. enelensis* and resembling them in every regard except for the lack of golden colour and the characteristic “apricot note” to the odour. Briefly described and illustrated in Thorn et al.^11^; known from the Island of Newfoundland and Québec (Table 1).

A pale specimen from the US Midwest clustered phylogenetically with the US Midwest golden to whitish species, *C. phasmatis,* with strong bootstrap support (Fig. 2A), and well separated from *C. tenuithrix*, a golden to white chanterelle from the Southeastern US, *C. flavus*, a golden species from the US Midwest, *C. deceptivus*, a golden to white chanterelle from the US Midwest, *C. pallens,* a golden-white species from Europe, *C. subalbidus,* a white chanterelle from the Pacific Coast of North America, and *C. cascadensis,* a golden chanterelle with white hymenophore from the Pacific Coast of North America. Sequences of LSU and *Tef-1* of a Chinese specimen identified as *C. cibarius*, for which a draft genome was recently published^18^, placed this taxon in the unresolved clade of *C. pallens–C. phasmatis*, i.e., outside the core *C. cibarius* clade (Fig. 2A).

Newly designed primers were used to amplify and sequence portions of the phytoene desaturase gene *Al-1* (1381 bases) and dual-function phytoene synthase/lycopene cyclase gene *Al-2* (1752 bases) in the white and gold variants of *C. enelensis*. The *Al-1* gene in the white chanterelle differed from that of the gold one by a 3-base deletion, resulting in the loss of a phenylalanine in the predicted gene product, and 5 single nucleotide substitutions, of which 4 were determined to be synonymous, but a fifth was predicted to result in the replacement of valine with phenylalanine in the gene product. The *Al-2* gene of the white variant differed from that of the gold one by two base substitutions, one synonymous and another predicted to alter an arginine residue shared with *Neurospora*^19^ to a histidine.

Typical chromatograms of high-performance liquid chromatography (HPLC) pigments in acetone extracts from white and golden variants of *C. enelensis* are presented in Fig. 2B. Comparison of the chromatograms indicates a single distinct peak with a retention time of 10.82 min characteristic for ß-carotene^20^ in the golden sample, while the white mutant of *C. enelensis* does not exhibit any pigments peaks detectable by the HPLC method used. Furthermore, the absorption spectrum (Fig. 2C) of the acetone extract from golden *C. enelensis* samples exhibits three peaks at 430, 454, and 481 nm, typical for ß-carotene, which were lacking in the white sample. Thus, both HPLC and spectroscopic analyses allow us to identify the presence of ß-carotene in the golden variant of *C. enelensis*.

**Figure 3:**
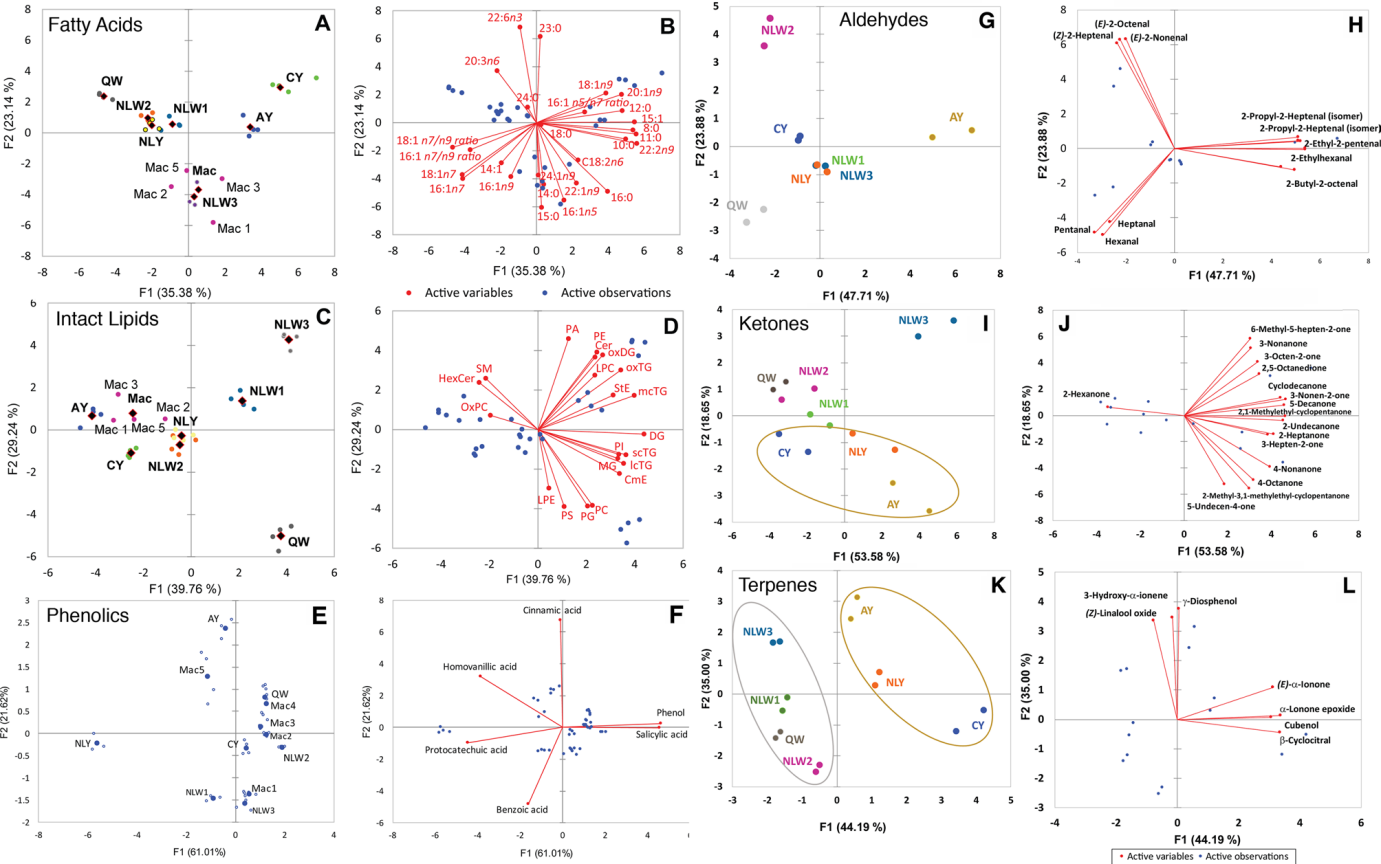
Principal components analysis (PCA) of chemical constituents of white and golden variants of Newfoundland chanterelles, showing the observations (sample clustering) and biplots showing loadings of chemical variables. (**A,B**) Fatty acids; (**C,D**) intact lipids; (**E,F**) phenolics. (**G–L**) Volatile compounds detected by headspace solid phase microextraction tandem mass spectrometry (HS-SPME-MS/MS). (**G,H**) Aldehydes; (**I,J**) ketones (ellipse highlighting the golden chanterelles); (**K,L**) terpenes (ellipses highlighting the white and golden chanterelles). Sample codes AY: *Cantharellus betularum* (golden); CY: *C. camphoratus* (golden); Mac1–Mac5: individuals from a mixture of golden and white NL chanterelles; NLW1–3: *C. enelensis* (Newfoundland, white 1–3); NLY: *C. enelensis* (Newfoundland, golden); QW: *C. enelensis* (Québec, white). For identities of fatty acids and intact lipids, see Table 2.

Chanterelles presented a complex fatty acid profile with constituents ranging from C8:0 to C24:1n9 (Table 2) with total saturated fatty acids ranging from 8.04 to 12.33 nmole%, monounsaturated fatty acids (MUFA) from 6.41 to 24.11 nmole %, *n*6-polyunsaturated fatty acids (PUFA) from 61.27 to 79.16 nmole% and *n*3-PUFA from 0.71 to 2.66 nmole% (Table 2). Golden variants of *C. camphoratus and C. betularum* were segregated in the upper right quadrant of the PCA observation and biplots based on the level of C23, C18:1n9, C20:1n9, C12:0 and C15:1 fatty acids (Fig. 3A,B)*.* Both the white (QC and NL) and golden variants of *C. enelensis* clustered together in upper left quadrant based on the combined levels of C24, C20:3n6 and C22:n6 fatty acids*.* The ratios of C18:1n7/C18:1n9 and of C16:1n5/C16:1n7 fatty acids were the most effective in discriminating the samples. All NL white *C. enelensis* had similar C18:1n7/C18:1n9 ratios (2.16 to 2.37) compared to significantly higher ratios in QC white *C. enelensis* (5.08) and the golden variants of NL *C. enelensis* (3.49). Conversely, the golden species *C. betularum* and *C. camphoratus* had significantly lower ratios of C18:1n7/C18:1n9 fatty acids (0.48 and 0.06, respectively) compared to all the other samples evaluated. These two species also had strikingly higher ratios of C16:1n5/C16:1n7 fatty acids (9.9 and 2.4, respectively) compared to all samples of *C. enelensis* (which ranged from 0.1 to 0.9; Table 2).

**Table 2:** Lipid composition (nanomole percent ± standard error) observed in white and golden variants of Newfoundland chanterelles.

	NLW1	NLW2	NLW3	QW	Mac	NLY	AY	CY
**Polar lipids**								
LPC	19.85 ± 2.50^a^	1.64 ± 0.11^c^	10.11 ± 1.32^b^	0.20 ± 0.01^c^	0.67 ± 0.07^c^	1.00 ± 0.10^c^	0.87 ± 0.05^c^	0.55 ± 0.03^c^
LPE	0.09 ± 0.05^abc^	0.21 ± 0.01^a^	0.0026 ± 0.0008^c^	0.16 ± 0.09^ab^	0.05 ± 0.02^bc^	0.19 ± 0.06^ab^	0.11 ± 0.04^abc^	0.11 ± 0.03^abc^
PA	18.50 ± 0.67^e^	20.58 ± 1.83^de^	26.17 ± 0.51^ab^	6.53 ± 0.48^g^	27.44 ± 1.18^a^	23.73 ± 0.19^bc^	22.41 ± 1.45^cd^	14.33 ± 0.47f.
PC	36.95 ± 1.08^ fg^	51.59 ± 3.33^bc^	33.46 ± 0.60^g^	73.86 ± 0.85^a^	40.75 ± 2.65^ef^	45.19 ± 0.60^de^	48.96 ± 2.61^cd^	55.74 ± 1.09^b^
OxPC	0.17 ± 0.01^cd^	0.42 ± 0.09^a^	0.12 ± 0.03^cd^	0.03 ± 0.01^d^	0.024 ± 0.008^d^	0.25 ± 0.02^bc^	0.484 ± 0.130^a^	0.35 ± 0.02^ab^
PE	21.08 ± 0.85^c^	22.48 ± 1.70^c^	27.91 ± 0.51^a^	12.61 ± 0.52^d^	27.03 ± 1.49^a^	25.88 ± 0.36^ab^	23.17 ± 1.55^bc^	20.41 ± 0.46^c^
PG	0.03 ± 0.02^d^	0.19 ± 0.04^b^	0.012 ± 0.002^d^	0.42 ± 0.07^a^	0.08 ± 0.03^cd^	0.15 ± 0.04^bc^	0.004 ± 0.002^d^	0.027 ± 0.005^d^
PI	0.052 ± 0.006^c^	0.082 ± 0.007^b^	0.064 ± 0.001^c^	0.086 ± 0.004^b^	0.094 ± 0.009^ab^	0.105 ± 0.007^a^	0.007 ± 0.004^d^	0.0010 ± 0.0007^d^
PS	2.79 ± 0.06^c^	2.80 ± 0.21^c^	1.781 ± 0.035^d^	6.07 ± 0.09^b^	3.07 ± 0.15^c^	2.95 ± 0.07^c^	2.98 ± 0.19^c^	7.82 ± 0.19^a^
SM	0.49 ± 0.14^bc^	0.008 ± 0.005^d^	0.362 ± 0.096^c^	0.035 ± 0.004^d^	0.80 ± 0.24^ab^	0.554 ± 0.008^bc^	0.98 ± 0.07^a^	0.66 ± 0.03^bc^
Total	100	100	100	100	100	100	100	100
**Neutral lipids**								
Cer	0.100 ± 0.004^c^	0.140 ± 0.004^bc^	0.262 ± 0.018^ab^	0.043 ± 0.001^c^	0.37 ± 0.13 ^a^	0.114 ± 0.008^c^	0.12 ± 0.01^c^	0.12 ± 0.01^c^
HexCer	4.72 ± 0.24^c^	1.55 ± 0.04^c^	5.95 ± 0.18^bc^	0.965 ± 0.012^c^	26.06 ± 7.71 ^a^	2.12 ± 0.06^c^	25.05 ± 0.22^a^	13.35 ± 0.55^b^
CmE	0.12 ± 0.01^bc^	0.17 ± 0.02^ab^	0.182 ± 0.009^ab^	0.216 ± 0.007^ab^	0.20 ± 0.09^ab^	0.062 ± 0.004^c^	0.124 ± 0.003^bc^	0.24 ± 0.02^a^
StE	0.024 ± 0.001^b^	0.035 ± 0.001^a^	0.023 ± 0.001^b^	0.012 ± 0.003^c^	0.04 ± 0.01^a^	0.0162 ± 0.0003^bc^	0.0182 ± 0.0003^bc^	0.0104 ± 0.0003^c^
MG	1.47 ± 0.09^ab^	2.51 ± 1.25^a^	1.41 ± 0.44^ab^	1.83 ± 0.20^ab^	0.45 ± 0.28^b^	0.87 ± 0.24^b^	0.53 ± 0.03^b^	0.49 ± 0.15^b^
DG	10.91 ± 0.39^cd^	18.19 ± 0.31^a^	15.84 ± 0.32^b^	11.91 ± 0.10^c^	15.32 ± 0.98^b^	10.53 ± 0.27^d^	6.61 ± 0.05^e^	7.84 ± 0.36^e^
oxDG	0.067 ± 0.009^b^	0.074 ± 0.008^b^	0.20 ± 0.03^a^	0.003 ± 0.001^c^	0.07 ± 0.01^b^	0.028 ± 0.008^c^	0.014 ± 0.002^c^	0.0017 ± 0.0005^c^
scTG	0.162 ± 0.002^de^	0.182 ± 0.005^cd^	0.225 ± 0.008^b^	0.201 ± 0.002^c^	0.07 ± 0.01f.	0.159 ± 0.002^e^	0.053 ± 0.004f.	0.327 ± 0.008^a^
mcTG	0.519 ± 0.023^d^	0.79 ± 0.02^c^	1.39 ± 0.02^a^	0.58 ± 0.02^d^	1.08 ± 0.17^b^	0.28 ± 0.02^e^	0.214 ± 0.003^e^	0.193 ± 0.008^e^
lcTG	78.33 ± 1.06^abc^	72.38 ± 0.89^cd^	66.75 ± 0.67^d^	82.54 ± 0.22^ab^	53.58 ± 7.59^e^	83.63 ± 0.39^a^	64.98 ± 0.32^d^	75.46 ± 1.06^bc^
oxTG	3.58 ± 0.47^bc^	3.98 ± 0.13^b^	7.77 ± 0.49^a^	1.70 ± 0.10^e^	2.76 ± 0.35^cd^	2.19 ± 0.07^de^	2.29 ± 0.07^de^	1.96 ± 0.11^cde^
Total	100	100	100	100	100	100	100	100
**Fatty acids**								
8:0	0.110 ± 0.006^bc^	0.085 ± 0.003^de^	0.130 ± 0.006^b^	0.065 ± 0.004^e^	0.13 ± 0.01^b^	0.094 ± 0.001^cd^	0.134 ± 0.009^b^	0.18 ± 0.02^a^
10:0	0.083 ± 0.005	0.06 ± 0.01	0.08 ± 0.01	0.028 ± 0.004	0.118 ± 0.009	0.06 ± 0.01	0.12 ± 0.02	0.14 ± 0.02^a^
11:0	0.301 ± 0.008^c^	0.232 ± 0.008^d^	0.354 ± 0.008^c^	0.178 ± 0.008^d^	0.44 ± 0.04^b^	0.30 ± 0.02^c^	0.49 ± 0.03^ab^	0.54 ± 0.03^a^
12:0	0.060 ± 0.004^de^	0.051 ± 0.004^e^	0.074 ± 0.007^bd^	0.062 ± 0.003^de^	0.079 ± 0.007^b^	0.052 ± 0.002^de^	0.094 ± 0.006^ab^	0.11 ± 0.02^a^
14:0	0.05 ± 0.01^c^	0.073 ± 0.008^ab^	0.094 ± 0.004^a^	0.059 ± 0.005^c^	0.09 ± 0.02^ab^	0.062 ± 0.006^b^	0.074 ± 0.009^ab^	0.063 ± 0.007^b^
15:0	0.096 ± 0.004	0.034 ± 0.003	0.12 ± 0.03	0.032 ± 0.006	0.19 ± 0.03	0.070 ± 0.005	0.103 ± 0.004	*ND*
16:0	6.06 ± 0.05^c^	5.68 ± 0.05^c^	8.54 ± 0.02^a^	3.99 ± 0.05^d^	8.34 ± 0.91^a^	6.26 ± 0.05^c^	7.77 ± 0.04^ab^	7.29 ± 0.05^b^
18:0	1.73 ± 0.01^b^	1.37 ± 0.02^d^	2.43 ± 0.01^a^	1.57 ± 0.03^c^	0.79 ± 0.13^e^	1.84 ± 0.02^b^	1.36 ± 0.03^d^	1.84 ± 0.02^b^
23:0	0.167 ± 0.008^d^	0.191 ± 0.004^b^	0.09 ± 0.01f.	0.219 ± 0.009^ab^	0.13 ± 0.01^e^	0.197 ± 0.007^bc^	0.16 ± 0.01^de^	0.25 ± 0.01^a^
24:0	0.27 ± 0.01^c^	0.259 ± 0.007^c^	0.419 ± 0.009^a^	0.394 ± 0.004^a^	0.18 ± 0.03^d^	0.34 ± 0.01^b^	0.24 ± 0.01^c^	0.37 ± 0.03^ab^
14:1	*ND*	0.035 ± 0.003	0.02 ± 0.01	0.033 ± 0.005	0. 04 ± 0.02	*ND*	*ND*	*ND*
15:1	0.09 ± 0.01^de^	0.065 ± 0.002^ef^	0.097 ± 0.003^d^	0.046 ± 0.004^f^	0.11 ± 0.01^c^	0.09 ± 0.01^de^	0.14 ± 0.01^b^	0.161 ± 0.004^a^
16:1*n*9	0.156 ± 0.006^d^	0.321 ± 0.009^b^	0.280 ± 0.002^c^	0.355 ± 0.007^b^	0.58 ± 0.05^a^	0.175 ± 0.007^d^	0.187 ± 0.008^d^	0.164 ± 0.005^d^
16:1*n*7	0.637 ± 0.008^d^	0.735 ± 0.01^d^	1.383 ± 0.003^a^	1.218 ± 0.02^b^	1.16 ± 0.06^b^	1.06 ± 0.01^c^	0.05 ± 0.01^e^	0.10 ± 0.05^e^
16:1*n*5	0.319 ± 0.003	0.279 ± 0.004	1.31 ± 0.02	0.108 ± 0.008	0.53 ± 0.10	0.35 ± 0.01	0.67 ± 0.03	0.26 ± 0.02
18:1*n*9	2.91 ± 0.02^c^	2.421 ± 0.008^d^	5.66 ± 0.02^b^	2.10 ± 0.09^e^	1.77 ± 0.11^f^	2.59 ± 0.05^d^	2.12 ± 0.02^e^	13.05 ± 0.09^a^
18:1*n*7	6.29 ± 0.02^e^	5.74 ± 0.03^e^	12.67 ± 0.02^a^	10.62 ± 0.10^b^	7.88 ± 0.55^d^	9.04 ± 0.03^c^	1.01 ± 0.02^f^	0.753 ± 0.004^f^
20:1*n*9	*ND*	*ND*	0.141 ± 0.006	*ND*	*ND*	*ND*	0.128 ± 0.005	0.42 ± 0.02
22:1*n*9	0.13 ± 0.01^b^	0.137 ± 0.006^b^	0.183 ± 0.002^a^	*ND*	0.14 ± 0.03^b^	0.11 ± 0.01^cd^	0.17 ± 0.02^ab^	0.086 ± 0.008^d^
24:1*n*9	2.02 ± 0.02	2.23 ± 0.04	2.369 ± 0.007	0.311 ± 0.005	1.42 ± 0.27	1.62 ± 0.02	1.94 ± 0.02	0.67 ± 0.01
18:2*n*6	31.27 ± 0.04^d^	26.92 ± 0.07^e^	32.47 ± 0.04^d^	29.67 ± 0.11^de^	45.10 ± 3.91^a^	33.86 ± 0.08^c^	29.76 ± 0.10^de^	39.02 ± 0.11^b^
20:3*n*6	43.91 ± 0.03^b^	49.68 ± 0.22^a^	28.80 ± 0.07^d^	45.59 ± 0.16^ab^	27.80 ± 4.96^d^	38.15 ± 0.13^c^	49.40 ± 0.09^a^	29.77 ± 0.08^d^
22:2*n*9	1.30 ± 0.05^c^	1.01 ± 0.03^d^	1.59 ± 0.03^b^	0.71 ± 0.02^e^	1.74 ± 0.14^b^	1.25 ± 0.05^c^	2.00 ± 0.07^a^	2.11 ± 0.07^a^
22:6*n*3	2.05 ± 0.01^c^	2.38 ± 0.01^b^	0.71 ± 0.02f.	2.66 ± 0.05^a^	1.24 ± 0.13^e^	2.42 ± 0.03^b^	1.88 ± 0.01^d^	2.63 ± 0.05^a^
Total	100	100	100	100	100	100	100	100
SFA	8.92 ± 0.04^d^	8.04 ± 0.05^d^	12.33 ± 0.04^a^	6.60 ± 0.07^e^	10.49 ± 1.14^b^	9.28 ± 0.05^c^	10.55 ± 0.09^b^	10.80 ± 0.04^b^
MUFA	12.54 ± 0.04^e^	11.97 ± 0.10^e^	24.11 ± 0.05^a^	14.78 ± 0.10^c^	13.62 ± 0.70^d^	15.03 ± 0.05^b^	6.41 ± 0.03f.	15.66 ± 0.09^b^
*n*6-PUFA	75.18 ± 0.05^b^	76.60 ± 0.15^b^	61.27 ± 0.04^e^	75.25 ± 0.08^b^	72.90 ± 1.65^c^	72.02 ± 0.10^c^	79.16 ± 0.16^a^	68.80 ± 0.06^d^
*n*3-PUFA	2.05 ± 0.01^c^	2.38 ± 0.01^b^	0.71 ± 0.02f.	2.66 ± 0.05^a^	1.24 ± 0.13^e^	2.42 ± 0.03^b^	1.88 ± 0.02^d^	2.63 ± 0.049^a^
18:1*n*7/*n*9	2.17 ± 0.02^d^	2.37 ± 0.02^d^	2.237 ± 0.007^d^	5.08 ± 0.24^a^	4.46 ± 0.21^b^	3.49 ± 0.08^c^	0.475 ± 0.005^e^	0.058 ± 0.001f.
16:1*n*7/*n*9	4.11 ± 0.12^c^	2.26 ± 0.02^e^	4.94 ± 0.04^b^	3.40 ± 0.02^d^	2.04 ± 0.19^e^	6.12 ± 0.27^a^	0.26 ± 0.09f.	0.61 ± 0.29f.
16:1*n*5/*n*7	0.50 ± 0.007^b^	0.39 ± 0.006^b^	0.94 ± 0.01^b^	0.09 ± 0.006^b^	0.47 ± 0.10^b^	0.33 ± 0.007^b^	9.94 ± 3.01^a^	2.39 ± 0.74^b^

Values (nanomole percent by weight composition) represent means ± standard errors for four replicates.

*ND* not detected, *SFA* saturated fatty acids, *MUFA* monounsaturated fatty acids, *PUFA* polyunsaturated fatty acids, *n* position position of the first double bond counted from methyl end group of unsaturated fatty acid.

Sample codes AY: *Cantharellus betularum* (golden); CY: *C. camphoratus* (golden); Mac: average of Mac1–Mac5 from a mixture of golden and white NL chanterelles; NLW1–3: *C. enelensis* (Newfoundland, white 1–3); NLY: *C. enelensis* (Newfoundland, golden); QW: *C. enelensis* (Québec, white). Lipid acronyms: *Cer* ceramide, *CmE* campesterol ester, *DG* diacylglycerol, *HexCer* hexanoyl ceramide (cerebroside), *lcTG* long-chain triacylglycerol, *LPC* lysophosphatidylcholine, *LPE*: lysophosphatidylethanolamine, *mcTG* medium-chain triacylglycerol, *MG* Monoacylglycerol, *oxDG* oxidized diacylglycerol, *OxPC* oxidized phosphatidylcholine, *oxTG* oxidized triacylglycerol, *PA* phosphatidic acid, *PC* phosphatidylcholine, *PE* phosphatidylethanolamine, *PG* phosphatidylglycerol, *PI* phosphatidylinositol, *PS* phosphatidylserine, *scTG* short-chain triacylglycerol, *SM* sphingomyelin, *StE* stigmasterol ester.

Means in the same row accompanied by different superscripts are significantly different among chanterelles at α = 0.05.

Intact lipids were more discriminatory than the fatty acids in segregating the NL and QC white *C. enelensis* samples. White NL *C. enelensis* clustered in the upper right quadrant based on the levels of phosphatidic acid (PA), phosphatidylethanolamine (PE), ceramide (Cer), oxidized diacylglycerol (oxDG), oxidized triacylglycerol (OxTG), lysophosphatidylcholine (LPC), stigmasterol ester (StE), and medium-chain triacylglycerol (McTG), while QC white *C. enelensis* clustered in the lower right quadrant based on the level of lysophosphatidylethanolamine (LPE), phosphatidylserine (PS), phosphatidylglycerol (PG), phosphatidylcholine (PC), campesterol ester (CmE), monoacylglycerol (MG), long-chain triacylglycerol (lcTG), short-chain triacylglycerol (scTG), and Diacylglycerol (DG). The intact lipids were also effective in segregating the golden variants of *C. enelensis, C. betularum* and *C. camphoratus* in the two left quadrants of the observation and biplots (Fig. 3C,D). Sphingomyelin (SM), hexanoyl ceramide (cerebroside, HexCer) and oxidized phosphatidylcholine (OxPC) clustered in the upper left quadrant with the Mac samples (*C. enelensis*) and *C. betularum*. Polar lipid composition was dominated by PA, PC and PE in all the samples evaluated. Québec white *C. enelensis* had significantly higher PC, but lower PA and PE compared to NL white or golden *C. enelensis*, and golden variants of *C. betularum* and *C. camphoratus* (Table 2). The neutral lipid composition was dominated by HexCer, DG and lcTG. Golden *C. betularum* and *C. camphoratus* had significantly higher levels of HexCer and lower levels of DG (Table 2).

Principal component analysis demonstrated that the phenolic acids were very effective in segregating the golden variants of *C. enelensis, C. betularum* and *C. camphoratus* from each other (Fig. 3E). *Cantharellus betularum* clustered in the upper left quadrant based on the levels of cinnamic and homovanillic acids, *C. camphoratus* in the lower right quadrant with salicylic acids, while *C. enelensis* was in lower left quadrant with protocatechuic and benzoic acids (Fig. 3E,F). Phenolic acids were the only components that separated the Mac samples, which are a mixture of golden and white fruiting bodies of *C. enelensis* that were indistinguishable from each other visually once dried; Mac5 sample clustered between the golden *C. enelensis* (NLY) and *C. betularum* (AY), while the other Mac samples clustered with the white *C. enelensis* samples in the right quadrants. The phenolic composition of the samples was dominated by phenol, with the golden variant of NL *C. enelensi*s having lower levels of phenol and salicylic acid and higher levels of protocatechuic and homovanillic acids compared to the others (Table 3, Fig. 3).

**Table 3:** Phenolic acid compounds (nanomole percent ± standard error) observed in white and golden variants of Newfoundland chanterelles.

Chanterelles	Benzoic acid	Salicylic acid	Phenol	Cinnamic acid	Protocatechoic acid	Homovanillic acid
Mac1	5.84 ± 0.09	4.93 ± 0.14	81.79 ± 1.43	–	5.58 ± 0.06	3.60 ± 0.14
Mac2	1.23 ± 0.06	5.23 ± 0.11	85.03 ± 0.28	–	4.49 ± 0.13	4.01 ± 0.13
Mac3	0.63 ± 0.06	5.11 ± 0.07	82.80 ± 0.32	–	6.97 ± 0.24	4.49 ± 0.05
Mac4	1.32 ± 0.03	4.97 ± 0.09	86.12 ± 0.12	0.01 ± 0.00	5.25 ± 0.16	2.33 ± 0.18
Mac5	2.91 ± 0.10	4.20 ± 0.15	68.54 ± 0.57	0.02 ± 0.00	10.89 ± 0.35	14.03 ± 0.50
AY	2.58 ± 0.20	4.40 ± 0.14	73.74 ± 0.77	0.04 ± 0.00	9.62 ± 0.32	9.63 ± 0.59
QW	1.07 ± 0.08	5.03 ± 0.07	87.69 ± 1.31	0.01 ± 0.00	3.03 ± 0.01	5.28 ± 0.18
CY	2.67 ± 0.14	4.74 ± 0.02	81.54 ± 1.53	–	7.80 ± 0.26	6.04 ± 0.61
NLY	3.78 ± 0.27	1.72 ± 0.07	30.54 ± 1.16	–	46.10 ± 1.70	18.26 ± 0.44
NLW1	4.98 ± 0.12	4.01 ± 0.20	66.72 ± 0.67	–	22.97 ± 0.71	1.32 ± 0.14
NLW2	1.34 ± 0.01	5.31 ± 0.08	90.51 ± 0.17	–	2.60 ± 0.14	–
NLW3	6.56 ± 0.33	4.88 ± 0.16	79.71 ± 0.60	–	5.66 ± 0.40	3.56 ± 0.25

Values (nanomole percent by weight composition) represent means ± standard errors for four replicates. Sample codes AY: *Cantharellus betularum* (golden); CY: *C. camphoratus* (golden); Mac1–Mac5: individuals from a mixture of golden and white NL chanterelles; NLW1–3: *C. enelensis* (Newfoundland, white 1–3); NLY: *C. enelensis* (Newfoundland, golden); QW: *C. enelensis* (Québec, white).

Headspace SPME detected 125 volatile organic compounds from the mushroom samples, including aldehydes, acids and esters, alcohols, ketones, furan derivatives, terpenes, and unidentified compounds (Table 4), but combined analysis of all analytes did not yield consistent separation of white from golden samples of *C. enelensis* in the first two components of the PCA plot (Fig. S1). The individual PCA analysis of the volatiles belonging to these classes was performed to determine whether the chanterelle samples were differentiated based on key volatile markers from the same chemical class. The analyses demonstrated that white and golden chanterelle samples clustered separately based on aldehydes, ketones and terpenes. In particular, *C. betularum* was rich in substituted aldehydes such as 2-ethyl-2-hexenal, 2-propyl-2-heptenal, and 2-butyl-2-octenal, which were much less abundant in *C. camphoratus* and both white and golden *C. enelensis* (Fig. 3G,H, Table 4). The PCA biplot of the ketones detected separated all white samples of *C. enelensis*, in the positive quadrants of F2, from all golden samples of *C. betularum, C. camphoratus*, and *C. enelensis*, located in the negative quadrants. These latter are characterized by greater quantities of 4-nonanone (versus 3-nonanone and 3-nonen-2-one), 4-octanone (versus 3-octen-2-one and 2,5-octendione), and cyclopentanone (Fig. 3I,J, Table 4). Similarly, the PCA biplot of the terpenes detected separated all white samples of *C. enelensis* in the negative quadrant of F1, from all golden samples of *C. betularum, C. camphoratus*, and *C. enelensis* in the positive quadrants, the latter characterized by greater abundances of (*E*)-α-ionone, α-ionon-5,6-epoxy-cubenol, and *β*-cyclocitral (Fig. 3K,L, Table 4).

**Table 4:** Abundances of the volatile metabolites, expressed as area counts of the mass spectra base peak (bp) of each compound × 10^–6^, from the headspace of white (QW, NLW1-3) and golden (CY, NLY, AY) variants of Newfoundland chanterelles extracted by SPME and separated, identified and semi-quantified by GC/MS.

No	RT (min)	Compounds (MW)	Bp	QW	NLW1	NLW2	NLW3	CY	NLY	AY
**Aldehydes**										
3	1.8	3-Methyl-butanal (86)	44	15.1 ± 0.8^abc^	12.3 ± 2.02^bc^	21.4 ± 1.9^ab^	5.3 ± 0.2^c^	24.2 ± 7.3^a^	12.9 ± 0.5^bc^	17.7 ± 1.7^ab^
6	2.1	Pentanal (86)	44	150.5 ± 23.4	85.5 ± 2.85	60.9 ± 2.8	44.6 ± 1.9	56.0 ± 0.4	66.8 ± 5.7	53.2 ± 1.7
10	3.4	Hexanal (100)	56	1107.5 ± 148.8^a^	620.9 ± 38.9^bc^	471.9 ± 7.5^cd^	502.0 ± 2.9^bcd^	476.8 ± 84.5^cd^	737.7 ± 73.9^b^	315.9 ± 3.4^d^
20	6.7	Heptanal (114)	70	105.4 ± 18.4^a^	37.8 ± 4.6^bcd^	31.6 ± 0.9^cd^	56.6 ± 0.7^bc^	24.8 ± 8.1^d^	62.62 ± 8.30^b^	17.8 ± 0.8^d^
22	8.1	2-Ethyl-2-pentenal (112)	55	5.0 ± 1.1^b^	7.6 ± 0.6^b^	7.0 ± 2.2^b^	5.6 ± 1.3^b^	4.1 ± 0.4^b^	16.59 ± 2.89^b^	47.8 ± 9.3^a^
26	9.9	2-Ethylhexanal (128)	57	0.8 ± 0.2^c^	6.3 ± 0.1^b^	1.1 ± 0.6^c^	0.4 ± 0.1^c^	2.5 ± 1.0^bc^	1.92 ± 0.4^c^	18.0 ± 2.8^a^
27	10.2	(*Z*)-2-heptenal (112)	41	34.9 ± 5.5^b^	30.3 ± 3.7^b^	71.3 ± 5.4^a^	23.1 ± 0.4^b^	39.6 ± 9.8^b^	39.52 ± 3.0^b^	28.9 ± 2.1^b^
28	10.6	Benzaldehyde (106)	105	106.0 ± 17.1^bc^	57.6 ± 9.9^c^	181.0 ± 25.9^a^	58.9 ± 9.8^c^	59.4 ± 24.9^c^	68.5 ± 16.7^c^	150.9 ± 12.8^ab^
35	12.9	2-Ethyl-2-hexenal (126)	55	16.6 ± 4.5^d^	331.5 ± 104.9^bc^	112.2 ± 40.3^cd^	79.5 ± 10.2^cd^	28.1 ± 12.9^cd^	508.0 ± 134.2^b^	1420.7 ± 166.0^a^
49	16.5	(*E*)-2-octenal (126)	70	23.9 ± 4.0^b^	20.2 ± 1.50^b^	72.6 ± 13.77^a^	34.5 ± 0.3^b^	19.8 ± 4.3^b^	26.6 ± 1.4^b^	21.2 ± 2.0^b^
72	18.3	(*E*)-2-nonenal (140)	41	5.8 ± 0.5^e^	10.9 ± 0.5^bc^	19.1 ± 0.7^a^	11.9 ± 0.5^b^	9.01 ± 0.6^cd^	8.7 ± 0.8^d^	6.1 ± 0.1^e^
75	21.9	2-Propyl-2-heptenal (154)	55	43.0 ± 4.1^e^	462.1 ± 54.7^bc^	102.5 ± 23.4^de^	269.3 ± 20.5^cd^	67.1 ± 16.7^de^	509.2 ± 55.2^b^	1331.0 ± 144.8^a^
77	22.5	2-Propyl-2-heptenal (isomer) (154)	55	5.5 ± 0.4^c^	18.4 ± 1.9^b^	5.0 ± 1.4^c^	21.2 ± 2.2^b^	17.4 ± 3.5^b^	16.7 ± 1.9^b^	29.5 ± 3.2^a^
78	23.0	2-Propyl-2-heptenal (isomer) (154)	55	35.4 ± 3.5^e^	286.1 ± 30.3^bc^	68.7 ± 11.9^de^	173.3 ± 11.9^cd^	40.5 ± 8.8^e^	313.1 ± 29.1^b^	892.8 ± 90.5^a^
111	29.1	2-Butyl-2-octenal (182)	55	124.4 ± 6.5^de^	159.6 ± 7.0^c^	109.9 ± 6.9^e^	437.8 ± 7.8^a^	145.2 ± 7.0^cd^	246.1 ± 8.9^b^	463.4 ± 12.3^a^
**Ketones**										
4	2.0	2-Pentanone (86)	43	47.4 ± 3.9^ab^	48.1 ± 2.3^a^	53.6 ± 8.5^a^	9.3 ± 0.2^c^	13.7 ± 1.2^c^	35.6 ± 0.0^b^	41.2 ± 0.8^ab^
7	2.6	2-Hexanone (100)	43	50.2 ± 3.7^a^	14.8 ± 0.3^cd^	46.3 ± 5.5^a^	8.4 ± 1.0^d^	26.3 ± 3.1^b^	13.2 ± 0.1^cd^	20.8 ± 0.1^bc^
16	6.1	2-Heptanone (114)	43	73.5 ± 18.6^ab^	71.1 ± 17.5^ab^	58.1 ± 15.1^b^	107.7 ± 10.9^ab^	54.6 ± 24.7^b^	126.5 ± 30.1^a^	115.2 ± 15.7^ab^
24	8.6	3-Hepten-2-one (112)	55	8.4 ± 1.2^c^	39.2 ± 7.9^bc^	62.7 ± 14.7^ab^	82.4 ± 12.8^ab^	8.2 ± 3.1^c^	82.1 ± 21.3^ab^	100.7 ± 18.4^a^
29	11.3	4-Octanone (128)	57	4.0 ± 0.0^b^	10.8 ± 3.5^b^	6.0 ± 2.6^b^	9.7 ± 3.0^b^	0.1 ± 0.0^b^	31.4 ± 9.4^a^	37.9 ± 7.9^a^
31	11.7	1-Octen-3-one (126)	55	23.9 ± 1.2^cd^	31.7 ± 1.2^cd^	115.6 ± 17.4^a^	73.5 ± 9.6^b^	7.1 ± 0.7^d^	36.9 ± 2.3^c^	11.3 ± 0.6^d^
33	12.2	Methyl-5-hepten-2-one (isomer) (126)	43	107.8 ± 29.3^b^	52.3 ± 16.3^b^	67.6 ± 31.9^b^	479.7 ± 130.0^a^	51.3 ± 25.5^b^	121.2 ± 35.5^b^	45.1 ± 7.9^b^
42	15.4	3-Octen-2-one (126)	55	625.2 ± 114.5	299.7 ± 58.5	318.2 ± 77.5	974.8 ± 114.2	210.3 ± 87.4	489.6 ± 88.4	544.6 ± 103.1
44	15.8	2-Nonanone (142)	43	10.7 ± 1.6	13.8 ± 3.0	8.86 ± 2.30	24.9 ± 1.1	51.9 ± 24.7	24.7 ± 4.8	38.4 ± 5.9
54	17.2	4-Nonanone (142)	43	5.8 ± 1.0^d^	55.9 ± 8.1^bcd^	28.7 ± 10.8^cd^	70.4 ± 15.9^bc^	6.8 ± 3.4^d^	88. 7 ± 10.7^b^	149.7 ± 33.4^a^
55	17.6	2-Methyl-3,1methyethyl-cyclopentanone (isomer) (140)	55	9.7 ± 0.3^c^	64.1 ± 13.9^bc^	16.6 ± 5.9^c^	42.5 ± 5.8^bc^	8.2 ± 3.9^c^	85.3 ± 18.9^b^	250.8 ± 43.2^a^
57	17.9	3-Nonanone (142)	72	31.3 ± 2.8^b^	16.4 ± 0.3^cd^	10.5 ± 2.8^d^	58.8 ± 4.6^a^	12.6 ± 1.9^d^	18.6 ± 1.7^cd^	24.2 ± 3.8^bc^
63	19.1	2,5-Octanedione (142)	43	2.5 ± 0.2^de^	18.3 ± 1.2^b^	7.1 ± 0.2^cd^	25.3 ± 3.0^a^	1.6 ± 0.4^e^	7.6 ± 2.6^cd^	12.4 ± 0.7^c^
69	20.3	3-Nonen-2-one (140)	55	8.5 ± 0.8^e^	23.6 ± 1.5^cd^	18.9 ± 4.1^de^	62.3 ± 3.8^a^	17.3 ± 4.3^de^	32.6 ± 3.5^c^	45.7 ± 1.9^b^
74	21.8	5-Decanone (156)	43	10.0 ± 0.6^b^	20.6 ± 2.0^b^	17.6 ± 4.0^b^	50.4 ± 7.1^a^	8.4 ± 2.2^b^	41.6 ± 5.0^a^	38.7 ± 0.6^a^
95	25.3	Cyclodecanone (182)	98	6.9 ± 0.3f.	8.9 ± 0.4^ef^	11.7 ± 1.1^d^	29.5 ± 1.1^a^	9.3 ± 0.3^e^	14.8 ± 0.3^c^	23.4 ± 0.2^b^
102	25.6	2-Undecanone (170)	58	57.2 ± 2.9	208.3 ± 10.9	157.1 ± 8.6	398.8 ± 13.5	136.5 ± 12.8	289.4 ± 11.5	341.8 ± 16.3
106	26.4	5-Undecen-4-one (*)(204)	55	0.1 ± 0.1^g^	49.6 ± 2.9^e^	31.5 ± 2.9^f^	84.7 ± 1.2^c^	191.8 ± 0.3^b^	70.2 ± 1.0^d^	223.1 ± 7.4^a^
**Terpenes**										
52	17.0	(*Z*)-linalool oxide (170)	59	8.5 ± 0.8^cd^	12.8 ± 1.4^bc^	4.9 ± 0.7^d^	19.8 ± 0.1^a^	8.9 ± 2.6^cd^	12.0 ± 1.2^bc^	14.9 ± 1.7^b^
80	23.5	*β-*Cyclocitral (152)	81	8.5 ± 1.2^d^	11.1 ± 1.0^cd^	20.7 ± 1.2^bc^	11.7 ± 1.9^cd^	52.2 ± 7.5^a^	28.0 ± 1.5^b^	19.5 ± 0.6^bc^
99	25.9	γ-Diosphenol (168)	55	25.9 ± 1.5^de^	36.3 ± 2.5^d^	19.2 ± 2.1^e^	85.8 ± 3.1^b^	34.6 ± 4.2^d^	57.3 ± 3.8^c^	128.3 ± 9.4^a^
118	32.5	(*E*)-α-Ionone (192)	121	1.7 ± 0.2	0.3 ± 0.0	1.0 ± 0.1	0.4 ± 0.0	223.3 ± 5.1	122.3 ± 0.8	58.9 ± 1.8
119	32.6	α-Lonone epoxide (208)	121	1.3 ± 0.1^c^	0.7 ± 0.0^c^	2.9 ± 0.2^c^	1.5 ± 0.0^c^	222.2 ± 5.6^a^	213.1 ± 3.9^a^	147.2 ± 4.5^b^
121	33.0	3-Hydroxy-α-ionene (208)	165	0.2 ± 0.0	1.8 ± 0.1	1.7 ± 0.1	4.0 ± 0.1	0.8 ± 0.1	2.9 ± 0.1	6.8 ± 0.1
124	36.9	Di-epi-1,10-cubenol (222)	105	0.7 ± 0.1^e^	1.3 ± 0.2^de^	17.5 ± 0.8^a^	3.2 ± 0.7^b^	2.8 ± 0.2^bc^	1.8 ± 0.1^cde^	2.6 ± 0.2^bcd^
125	37.3	Cubenol (222)	161	0.1 ± 0.0^d^	0.3 ± 0.1^cd^	0.7 ± 0.1^b^	0.3 ± 0.0^d^	1.4 ± 0.1^a^	0.5 ± 0.0^c^	0.9 ± 0.1^b^

Values (means ± standard errors; n = 2) represent the abundances, expressed as area counts of their mass spectra base peak (Bp) divided by 10^6^.

Rows with different letters show significant differences between treatments at α = 0.05.

*Tentatively identified; *Bp* base peak, *MW* molecular weight, *RT* retention time. Sample codes AY: *Cantharellus betularum* (golden); CY: *C. camphoratus* (golden); NLW1–3: *C. enelensis* (Newfoundland, white 1–3); NLY: *C. enelensis* (Newfoundland, golden); QW: *C. enelensis* (Québec, white).

## Discussion

In Europe, occasional white chanterelles have been recognized as albino forms of *Cantharellus amethysteus, C. cibarius, C. ferruginascens,* and *C. romagnesianus*, and the varieties named for their white colouration, *C. cibarius* var. *inodorus* and *C. cibarius* var. *gallaecicus*, have been reduced to synonymy of *C. cibarius* and *C. romagnesianus*, respectively^14^. The white chanterelles of Newfoundland and Québec are clearly conspecific with *C. enelensis* but, if precision is required, they may be referred to as *C. enelensis* f. *acolodorus*. The presence of typical and albino forms stands in contrast to some other species of chanterelles that are normally pallid, such as *C. subalbidus, C. pallens*, and *C. phasmatis*^14,21,22^, although in the latter two species, some specimens are particularly pale, as in the specimen of *C. phasmatis* sent to us from Minnesota or white individuals of *C. pallens* reported by Olariaga et al.^14^.

*Neurospora crassa* (Ascomycota) forms rapidly growing pinkish-orange cultures, with white variants that have been studied extensively due to the ease of culture of this species and the early availability of its genome sequence^19^. In albino variants of this species, a mutation of one of three genes, *Al-1, Al-2,* or *Al-3*, that encode for phytoene desaturase^23^, phytoene synthase^24^, and geranylgeranyl pyrophosphate synthetase^25^, respectively, causes the lack of carotenoid pigments through the loss of function of one of these enzymes required in the carotenoid biosynthetic pathway. We were able to detect sequence variants in the *Al-1* and *Al-2* genes of the white variant of the NL chanterelles but, because we do not have it in culture, we were unable to follow up with functional analyses.

In albino and wild-type variants studied in lab culture, the presence of carotenoid pigments may exhibit a benefit under certain environmental or physiological stress conditions, such as oxidative stress or high light exposure^26^. Under oxidative stress, free radicals react with the structural polyene chain of carotenoids, deflecting potential damage^27^. Beta-carotene has been shown to protect the photosensitized oxidation of phospholipid bilayers^28^, which has been observed in other fungi, including the ascomycete *Arthrobotrys ferox*^29^. In *N. crassa* under high light exposure, albino mutants have lower respiration rates of hyphal suspensions^30^. Given that the NL golden chanterelles, *C. enelensis*, are much more common than the white mutants, it is likely that they possess some ecophysiological advantage over the albinos, possibly conferred by their carotenoid pigments.

The white and golden variants of *C. enelensis* differ in far more than just carotenoid pigmentation, or the lack of it. Their chemical composition differs in lipids and fatty acids, phenolic acids, and multiple classes of volatile compounds, and these differences may affect their palatability to both human and invertebrate consumers. Among the lipids, the fatty acid composition of NL white and golden *C. enelensis* was dominated by C18:2n6, consistent with the composition of other species of edible mushrooms reported in the literature^31^. The ratio of C18:1n7/C18:1n9 fatty acids appears to be a particularly useful chemotaxonomy biomarker for differentiating C. *betularum, C. camphoratus*, and *C. enelensis* (Table 2, Fig. 3A,B), as well as distinguishing the QC and NL white mutants (Table 2). The C18:1n7/C18:1n9 ratios have similarly been shown to be very effective in the chemotaxonomic classification of 12 *Brassica* species^32^. The intact lipids reported in this paper represented both membrane and storage lipids. From a chemotaxonomy perspective, *C. betularum, C. camphoratus*, and golden individuals of *C. enelensis* appear to have a similar composition of intact membrane and storage lipids, placing them together in a PCA biplot, separated from three out of four samples of the white mutants of *C. enelensis,* which had more variety of lipids, from phosphatidic acid (PA) to lysophosphatidylethanolamine (LPE) (Fig. 3C,D). Hexanoyl ceramide (HexCER) is a sterol present in the fungal membrane^33,34^. Golden variants of *C. enelensis* (and one white sample, NLW2), plus *C. betularum* and *C. camphoratus* have similar levels of HexCER, whereas the other three samples of white *C. enelensis* have less. From a compositional perspective, phosphatidylcholine (PC) was the predominant membrane lipid and various forms of triacylglycerols are the major storage lipids of *C. betularum, C. enelensis* and *C. camphoratus,* consistent with other reports demonstrating these as the major membrane and storage lipids in edible mushrooms^31,35^.

Phenolic compounds are important in the detection and perception of organisms as well as in their response to biotic and abiotic stressors in their environment. As such, they have been a common choice of secondary compounds used as biomarkers in chemotaxonomic classification of plants, lichens and increasingly in non-lichenized fungi^36,37^. We found the phenolic acids subclass of phenolic compounds to be effective in differentiating golden *C. enelensis* (with more homovanillic and protocatechuic acids) from their white mutants (with less, and with more phenol and salicylic acid), as well as from the golden species *C. betularum* (with more cinnamic acid) and *C. camphoratus* (with less cinnamic acid and more benzoic acid). These results suggest that phenolic acids may be useful chemotaxonomic markers to differentiate different species of chanterelles in commerce. To the best of our knowledge this is the first study demonstrating the application of phenolic acids as chemotaxonomic markers in chanterelles.

Chanterelles are known to have very distinctive colours, flavours and fruity aromas that vary between species, although their perception also varies with their human assessors. In plants, fungi, and the fruit and fruiting bodies they produce, these characteristics are determined in part by the composition of aromatic or aliphatic volatile compounds present in individuals, often as a result of complex mixtures^38,39^. In this study, we observed over 100 volatile compounds in both golden and white chanterelles. Among these compounds, the aldehydes, ketones and terpenes appear to be the most effective as chemotaxonomic biomarkers to differentiate species and colour variants of chanterelles. The terpenes, particularly *α*-ionone, cubenol and *β*-cyclocitral, characterize and differentiate the golden chanterelles *C. enelensis, C. betularum,* and *C. camphoratus,* whereas all samples of *C. enelensis* f. *acolodorus* were characterized by a lower concentration of each of these compounds (Fig. 3K,L). A reduction of ionones in the albino mutant is unsurprising since these and related “rose ketones” are derived from the breakdown of carotenoids^40^; this absence may partly explain the perceived lack of an “apricot note” in the odour of the albino mushrooms^11^. Among the ketones, principal component 1 (PC1, roughly parallel to the ratio of hexanone:heptanone and undecanone) separates the three golden chanterelle species included, and PC2 separates white from all golden chanterelles (Fig. 3I,J). In contrast, two white mutant samples (QW and NLW2) and *C. betularum* are separated by the aldehydes hexanal, 2-octenal and ethyl-pentenal, respectively, leaving golden *C. enelensis, C. camphoratus*, and two other white samples of *C. enelensis* in a central cluster (Fig. 3G,H). Our work suggests that volatile aldehydes, ketones and terpenes can be used as chemotaxonomic markers to separate chanterelles based on species and colour. This knowledge could be useful to distinguish white from golden chanterelles after drying, which is often used to prolong shelf life or for convenience during food formulation, during which they become the same dull brownish orange colour.

Collectively, the output of the chemical analyses presented in this paper demonstrates for the first time the applications of metabolomics to separate chanterelles based on species, colour, aroma, and geography of production. The fatty acids, membrane and storage lipids, phenolic acids, volatile terpenes, aldehydes and ketones are presented as chemotaxonomic biomarkers that are useful in differentiating the recently discovered white mutant of *C. enelensis* from its golden relatives. In addition to its chemotaxonomic potential, this work raises questions as to the functional significance of these compounds in nature.

## Materials and methods

All fungal specimens were collected on Crown Lands for which no permission is required. Specimens of fresh, field-collected mushroom fruiting bodies were either air-dried at a temperature of 30–35 °C or frozen at − 80 °C until processing, and air-dried voucher specimens have been deposited at the Dr. Laurie L. Consaul Herbarium, London, Canada (UWO) and the National Mycological Herbarium of Canada, Ottawa (DAOM) (Table 1).

### DNA extraction, PCR amplification and sequencing

Genomic DNA was extracted from air-dried specimens following Thorn et al.^9^. Primers ITS1 and ITS6R were used to amplify the ITS region, LS1 and LR3 to amplify ~ 650 bases of the 5’-LSU region and Canth-ef1a983-F and Canth-ef1a-1567-R to amplify *Tef-1*^9,41–44^. The PCR products were checked using gel electrophoresis and successful products were cleaned using Bio Basic EZ-10 Spin Column PCR Products Purification Kit. Cleaned PCR products were submitted to the sequencing facility of Robarts Institute (University of Western Ontario) to obtain sequences through Sanger sequencing with amplification primers, and internal sequencing primers CanthITS1_Internal-R, 5.8S-R-Canth, and ITS86R-Canth for the ITS region^9^. New sequences produced for this study were deposited in GenBank as accessions MN181445–MN181461 and MN206911–MN206945.

### Phylogenetic analyses

Sequences of the ITS, LSU and *Tef-1* regions were cleaned and assembled with SeqEd v.1.03, then, together with sequences of related species downloaded from GenBank, each region was aligned separately with MAFFT v.7^45^ under the G-INS-i strategy, with “leave gappy regions” selected. The draft *Cantharellus cibarius* genome (QOWL00000000.1)^18^ was searched by BLASTn for ribosomal and *Tef-1* sequences. The full-length match to our *Tef-1* sequences was found in a single scaffold (QOWL01010594_RC), but no ITS sequences and only partial LSU sequences were found (in QOWL01010930_RC, QOWL01012415, QOWL01007530_RC, and QOWL01005980_RC). Alignments were imported into MEGA X^46,47^, trimmed and concatenated into a single ITS-LSU-*Tef-1* dataset, then optimized manually. Phylogenetic trees were constructed with maximum likelihood (ML), with 1000 bootstrapping replicates in MEGA X. Analyses were repeated with Bayesian inference using MrBayes 3.2.6 with 4 chains and 5 million generations, discarding the first 25% of trees, when the average standard deviation of split frequencies had stabilized below 0.01^48^. Tree topologies were compared, and Bayesian prior probabilities transferred to the ML bootstrap tree in Adobe Acrobat.

### Genetic analysis of carotenoid synthesis genes

In order to design primers to amplify portions of the *Al-1* and *Al-2* genes in white and golden chanterelles, these genes were first located in three published *Cantharellus* genome sequences^18^ using tBLASTn^49^ to query the genomes with protein sequences from *N. crassa* (PRJNA132; *Al-1* XM_959620.2 and *Al-2* XM_960632.3)^19^. Candidate gene sequences from *Cantharellus appalachiensis* (QLPK00000000.1; *Al-1* Scaffold 4647: QLPK01003932.1 and *Al-2* Scaffold 1419: QLPK01001208.1), *C. cibarius* (QOWL00000000.1; *Al-1* Scaffold 15338: QOWL01009792.1 and *Al-2* Scaffold 1560: QOWL01001169.1), and *C. cinnabarinus* (QLPJ00000000.1; *Al-1* Scaffold 1057: QLPJ01000880.1 and *Al-2* Scaffold 2474: QLPJ01001998.1) were annotated in Geneious using a discontiguous megablast against GenBank to search for homologous motifs^50^. Based on these alignments, putative ORFs were annotated and aligned, and an overlapping set of PCR primers for *AL-1* and *AL-2* were designed in Geneious (Table 5). Designed primers were tested for specificity using the BLAST algorithm against the three *Cantharellus* genomes^18^. PCR amplified products were assessed for quality, cleaned, sent for sequencing, and assembled as above. Assembled sequences of white and gold samples were compared, along with their putative amino acid products determined using ExPASy^51^, guided by the translations of the *Neurospora crassa Al-1* (XM_959620.2) and *Al-2* (XM_960632.3) genes. Partial sequences of the *Al-1* and *Al-2* genes of gold and white variants have been deposited in GenBank as MW442833–MW442836.

**Table 5:** PCR primers designed to amplify portions of the phytoene desaturase gene (*Al-1*) and phytoene synthase gene (*Al-2*) from *Cantharellus* species, based on genomic sequences from *Cantharellus appalachiensis*, *C. cibarius*, and *C. cinnabarinus*^18^, listed in “Materials and methods” section.

Gene	Primer name	Sequence	Product size (bp)
Phytoene desaturase (*Al-1*)	Al-1-F1	CACCGARAAGASTCACAGAARCCC	838
Phytoene desaturase (*Al-1*)	Al-1-R1	TGTSGTGTTGGTGCCTGTTGG	
Phytoene desaturase (*Al-1*)	Al-1-F2	GCACCACGRTCGAGGTTGAAC	996
Phytoene desaturase (*Al-1*)	Al-1-R2	CGTTYACATTCGCCTCSATGTAC	
Phytoene desaturase (*Al-1*)	Al-1-F3	TGGCAAAACCWCCGATAGGGTACC	1169
Phytoene desaturase (*Al-1*)	Al-1-R3	AGTCGCCATGATATCTGCGG	
Phytoene synthase (*Al-2*)	Al-2-F1	GTAACGAGGGTAGACCAGGC	1071
Phytoene synthase (*Al-2*)	Al-2-R1	CCTAGGTATGCCTTTTGCCG	
Phytoene synthase (*Al-2*)	Al-2-F2	AAATGCGAGCCTTCCTGTCC	919
Phytoene synthase (*Al-2*)	Al-2-R2	GAACAGGTAGCGGTGCATGG	
Phytoene synthase (*Al-2*)	Al-2-F3	ACGACGCACTCKAYGTCGAGATG	862
Phytoene synthase (*Al-2*)	Al-2-R3	RGATTACGATTTGGTGTASGTGACATG	

### Pigment analysis

Field-collected mushroom fruiting bodies were weighed while fresh, wrapped in aluminum foil, and frozen at − 80 °C for pigment analyses, and other samples were weighed fresh and then dried to obtain a conversion for fresh to dry weight. Pigments were extracted with ice-cold 100% acetone at 4 °C and dim light. The supernatant was filtered through a 0.22 µm syringe filter and samples were stored at − 80 °C until analysed. Pigments were separated and quantified by high-performance liquid chromatography (HPLC) as described previously^20^, with some modifications. The system consisted of a Beckman System Gold programmable solvent module 126, diode array detector module 168 (Beckman Instruments, San Ramon, California, USA), CSC-Spherisorb ODS-1 reverse-phase column (5 mm particle size, 25 × 0.46 cm I.D.) with an Upchurch Perisorb A guard column (both columns from Chromatographic Specialties Inc., Concord, Ontario, Canada). Samples were injected using a Beckman 210A sample injection valve with a 20 μL sample loop. Pigments were eluted isocratically for 6 min with a solvent system of acetonitrile:methanol:0.1 M Tris–HCl (pH 8.0), (72:8:3.5, v/v/v), followed by a 2 min linear gradient to 100% methanol:hexane (75:25, v/v) which continued isocratically for 4 min. Total run time was 12 min. Flow rate was 2 mL min^−1^. Absorbance was detected at 440 nm and peak areas were integrated by Beckman System Gold software. Retention times and response factors of Chl *a*, Chl *b*, lutein and ß-carotene were determined by injection of known amounts of pure standards purchased from Sigma (St. Louis, MO, USA). The retention times of zeaxanthin, antheraxanthin, violaxanthin and neoxanthin were determined by using pigments purified by thin-layer chromatography as described by Diaz et al.^52^.

### Extraction and analysis of chanterelle lipids

Samples of each chanterelle species were homogenized to fine powder in a cryomill (Reitch, Germany) and 100 mg of the homogenized powder mixed with 1 mL methanol (MeOH), 1 mL chloroform (CHCl_3_) and 0.8 mL water following Pham et al.^53^. The sample mixture was thoroughly vortexed, then centrifuged (Sorvall Legend XT/XF centrifuge; ThermoFisher Scientific, Mississauga, Ontario) at 2500 rpm for 15 min. The organic layer was transferred to new vials, dried under nitrogen and then reconstituted in 1 mL chloroform:methanol (1:1 v/v). Aliquots were then used for either gas chromatography with mass spectrometric and flame ionization detection (GC–MS/FID) or ultra-high-performance liquid chromatography with heated electrospray ionization high resolution accurate mass tandem mass spectrometric analysis (UHPLC-HESI-HRAM/MS–MS) for fatty acids and intact lipids analysis, respectively.

For GC–MS/FID analysis, chanterelle fatty acids were converted to fatty acid methyl esters (FAMEs) as follows: To 300 µL aliquot of the lipid extract, 50 μL of C18:0 alkane (1 mg mL^−1^ in chloroform: methanol 1:1 v/v) was added as internal standards and the samples dried under nitrogen and the fatty acids esterified by adding 400 µL methanolic HCl (1.5 N). The samples were then incubated in a pre-heated oven at 60 °C for 30 min. After incubation, 0.8 mL of distilled water was added to the cooled samples and the FAMEs extracted with 3 aliquots each of 500 μL of hexane. The fractions were combined, dried under N_2_, re-suspended in 50 μL hexane, and the FAMEs analyzed using a Trace 1300 gas chromatograph coupled to a Flame Ionization Detector and TSQ 8000 mass spectrometer (Thermo Fisher Scientific). The FAMEs were separated on a BPX70 high-resolution column (10 m × 0.1 mm ID × 0.2 μm, Canadian Life Science, Peterborough, Ontario) using helium as the carrier gas at a flow rate of 1 mL min^−1^. One μL of each sample was injected in split mode (1:15) using a Tri-plus auto-sampler (Thermo Fisher Scientific). The operation conditions were as follows: initial oven temperature set at 50 °C for 0.75 min, increased to 155 °C at 4 °C min^−1^, ramped to 210 °C at 6 °C min^−1^, then 240 °C at 15 °C min^−1^ and final temperature held for 2 min. Methylated fatty acids were determined by comparison with retention times and mass spectra obtained from commercial standards (Supelco 37 component mix, Supelco PUFA No. 3, Sigma Aldrich, Oakville, Ontario) and the NIST database (Thermo Fisher Scientific). Standard curves were employed to determine the amount of individual fatty acids, and values are presented as nmole%.

For the UHPLC-HESI-HRAM/MS–MS analysis, a Q-Exactive Orbitrap mass spectrometer (Thermo Fisher Scientific) coupled to an automated Dionex UltiMate 3000 UHPLC system was used to analyze the intact chanterelle lipids according to our previously published method^53^. Briefly, the intact lipids were resolved using an Accucore C30 column (150 mm × 2 mm I.D., particle size: 2.6 µm, pore diameter: 150 Å) and the following solvent systems: (i) Solvent A consisted of acetonitrile:water (60:40 v/v) containing 10 mM ammonium formate and 0.1% formic acid and (ii) Solvent B consisted of isopropanol:acetonitrile:water (90:10:1 v/v/v) with 10 mM ammonium formate and 0.1% formic acid. The conditions used for separation were 30 °C (column oven temperature), flow rate of 0.2 mL min^−1^, and 10 µL of sample injected. The gradient system used was as follow: solvent B increased to 30% in 3 min; 43% in 5 min, 50% in 1 min, 90% in 9 min, 99% in 8 min, and finally maintained at 99% for 4 min. The column was re-equilibrated for 5 min before each new injection. Full scans and tandem MS acquisitions were performed in both negative and positive modes using the following parameters: sheath gas: 40, auxiliary gas: 2, ion spray voltage: 3.2 kV, capillary temperature: 300 °C; S-lens RF: 30 V; mass range: 200–2000 m/z; full scan at 70,000 m/z resolution; top-20 data-dependent MS/MS resolution at 35,000 m/z, collision energy of 35 (arbitrary unit); injection time of 35 min for C30RP chromatography; isolation window: 1 m/z; automatic gain control target: 1e5 with dynamic exclusion setting of 5.0 s. The instrument was externally calibrated to 1 ppm using electrospray ionization (ESI); negative and positive calibration solutions (Thermo Fisher Scientific) were used to calibrate the instrument at 1 ppm. Tune parameters were optimized using PC 18:1(9Z)/18:1(9Z), Cer d18:1/18:1(9Z), PG 18:1(9Z)/18:1(9Z), sulfoquinovosyl diacylglycerols [SQDG] 18:3(9Z,12Z,15Z)/16:0, monogalactosyl diglyceride [MGDG] 18:3(9Z,12Z,15Z)/16:3(7Z,10Z,13Z), and digalactosyldiacylglycerol [DGDG] 18:3(9Z,12Z,15Z)/18:3(9Z,12Z,15Z) lipid standards (Avanti Polar Lipids, Alabaster, AL, USA) in both negative and positive ion modes. The data were processed using either X-Calibur 4.0 (Thermo Fisher Scientific) or LipidSearch version 4.1 (Mitsui Knowledge Industry, Tokyo, Japan) software packages.

### Phenolics analysis by GC–MS

Reagent grade phenolic acid standards including benzoic acids, p-hydroxybenzoic acid, vanillic acid, gallic acid, 3,4-dihydroxybenzoic acid, syringic acid, gentisic acid, veratric acid, salicylic acid, cinnamic acid, o-coumaric acid, m-coumaric acid, p-coumaric acid, ferulic acid, sinapic acid, caffeic acid, sodium hydroxide, N,O-Bis(trimethylsilyl)trifluoroacetamide (BSTFA-TCMS) were purchased from Sigma Aldrich. Methanol, ethyl acetate, and hydrochloric acid (36% w/v) were purchased from VWR (Mississauga, Ontario, Canada). For alkaline hydrolysis of powdered chanterelles, 100 µL of aqueous 3,4-dihydroxybenzoic acid solution (0.2 mg mL^−1^) was added to a mixture containing 4 g of sample in 8 mL 1 M sodium hydroxide. The resultant mixture was incubated in the dark for 24 h at 25 °C on an orbital shaker (50 rpm). The pH of the reaction mixture was adjusted to 2.0–2.5 using concentrated HCl then vortexed. The organic components were extracted four times with 4 mL methanol: ethyl acetate (1:3 ratio) into pre-weighed vials. The solvent was evaporated under nitrogen at 35 °C to determine the crude extraction yield. The extracts were resuspended in 1 mL ethyl acetate, vortexed, then 300 µL of extract transferred into a pre-weighed vial, dried under nitrogen, and 50 µL of BSTFA-TCMS and 50 µL of pyridine added. The resultant mixture was incubated at 70 °C in darkness for 30 min then transferred to GC vials for GC–MS analysis. Standard solutions were derivatized in a similar manner.

A Thermo Scientific Trace 1300 gas chromatograph coupled to a Triple Quad mass spectrometer (Thermo Fisher Scientific) was used for the analysis and the compounds resolved on a ZB-5MS non-polar stationary phase column (30 m × 0.25 mm I.D., 0.25 μm film thickness, Phenomenex, Torrance, CA, USA) with helium as the carrier gas (flow rate of 0.6 mL min^−1^). One microliter of the standard or sample was injected in basic mode (15:0) using a Tri-plus auto-sampler. The oven temperature program was as follows: the initial oven temperature was 70 °C (held for 1 min), was increased at 12 °C min^−1^ to 220 °C (held for 3 min), 15 °C min^−1^ to reach 250 °C and held for 1 min. Identification of the phenolic acids (as trimethylsilyl ether, TMS) was based on the comparison of their retention times and mass spectra with that of the NIST library and commercial standards, with quantities calculated and expressed as nmole%.

#### Analysis of the volatile profile of chanterelles by SPME-GC/MS

Volatile metabolites were extracted and analysed by Solid-Phase Microextraction and Gas Chromatography/Mass Spectrometry (SPME-GC/MS) following Vidal et al.^54^. Briefly, 100 mg of sample powder obtained after cryo homogenization was placed in 10 mL headspace glass vials and kept at 50 °C for 5 min (sample equilibration) before volatile metabolites extraction and analysis began. A divinylbenzene/carboxen/polydimethylsyloxane (DVB/CAR/ PDMS) coated fibre (1 cm long, 50/30 μm film thickness; Supelco, Sigma-Aldrich), was inserted into the headspace of the sample vial and held there for 60 min^55,56^. Chanterelle volatile composition was analyzed using a Trace 1300 gas chromatograph coupled to a TSQ 8000 Triple Quadrupole mass spectrometer (Thermo Fisher Scientific). The extracted volatile compounds were separated using a ZB-5MS non-polar stationary phase column (30 m × 0.25 mm I.D., 0.25 μm film thickness; Phenomenex) with He used as the carrier at a flow rate of 1 mL min^−1^. After extraction the fibre was desorbed for 10 min in the injection port and the instrument operated as follows: splitless mode with a purge time of 5 min, initial oven temperature set at 50 °C (5 min hold) and increased to 290 °C at 4 °C min^−1^ (2 min hold). Ion source and quadrupole mass analyzer temperatures were set at 230 and 150 °C respectively, injector and detector temperatures held at 250 and 290 °C respectively, mass spectra ionization energy set at 70 eV, and data acquisition done in scan mode. After each sample desorption, the fiber was cleaned for 10 min at 250 °C in the conditioning station. Volatile compounds were identified by matching the obtained mass spectra with those of available standards, and mass spectra from commercial libraries NIST/EPA/NIH (version 2.2, Thermo Fisher Scientific) or the scientific literature^55,56^. Volatile compounds in the chanterelle samples were semi-quantified based on the area counts × 10^−6^ of the base peak. Compounds with lower abundances than 10^−6^ area counts were considered as traces. Although the chromatographic response factor of each compound is different, the area counts determined are useful for comparison of the relative abundance of each compound in the different samples analysed^55,56^.

### Statistics and reproducibility

Results of analyses of lipids and phenolics are presented as means and standard errors of 4 replicates and those of head-space analyses of volatiles are based on 2 replicates (Tables 2, 3, 4). The values of all dependent variables were tested for normal distribution and homoscedasticity by Shapiro–Wilk and Levene tests, respectively. For variables with homogeneous variance, parametric one-way analysis of variance (ANOVA) was used to determine if there were significant differences between chanterelle samples. Where significance was detected, the means were compared with Fisher's Least Significant Difference (LSD), α = 0.05. Where the assumption of normality was not met (pentanal, 3-octen-2-one, 2-nonanone, 2-undecanone, (*E*)-α-Ionone, 3-hydroxy-α-ionene, all phenolic acids, and some minor fatty acids 10:0, 15:0, 14:1, 16:1*n*5, 20:1*n*9 and 24:1*n*9), no significance was detected after the data were treated with a non-parametric Kruskal–Wallis test and significance (p < 0.05) was adjusted using a post-hoc Bonferroni correction, in some cases despite clear numerical differences and separation in the first two factors of principal component analysis (PCA). PCA was conducted using XLSTAT Premium version (Addinsoft, Paris, France) to discern similarities or differences between the variants. Figures were prepared using XLSTAT Premium version and SigmaPlot 13.0 software programs (Systat Software Inc., San Jose, CA).

## Supplementary Information

Supplementary Information.

## Data Availability

All newly determined DNA sequences have been deposited in GenBank, including ribosomal ITS and LSU, *Tef1*, *Al-1* and *Al-2*.
