# Supplementary material for: Identification and analyses of the chemical composition of a naturally occurring albino mutant chanterelle
Source: Sci Rep. 2021 Oct 18;11:20590. doi: 10.1038/s41598-021-99787-8 (PMC8523663; doi:10.1038/s41598-021-99787-8)
Supplement: Supplementary file 1 — Supplementary Information. [file 41598_2021_99787_MOESM1_ESM.pdf]

Identification and analyses of the chemical composition of a naturally occurring albino mutant chanterelle

R. Greg Thorn[1*], Alicia Banwell[1], Thu Huong Pham[2], Natalia P. Vidal[2,a], Charles Felix Manful[2], Muhammad Nadeem[2], Alexander G. Ivanov[1,3], Beth Szyszka Mroz[1], Michael B. Bonneville[1], Norman Peter Andrew Hüner[1], Michele D. Piercey-Normore[2], and Raymond Thomas[2].

[1]Department of Biology, University of Western Ontario, 1151 Richmond St. N., London, ON N6A 5B7, Canada

[2]School of Science and the Environment, Grenfell Campus, Memorial University, 20 University Drive, Corner Brook, NL A2H 5G4, Canada

[3]Institute of Biophysics and Biomedical Engineering, Bulgarian Academy of Sciences, Acad. G. Bonchev str. Bl. 21, 1113 Sofia, Bulgaria

[a] Current Affiliation: Department of Food Science, iFOOD Multidisciplinary Center, Aarhus University, Agro Food Park 48, Aarhus N 8200, Denmark

[*]Corresponding author: rgthorn@uwo.ca

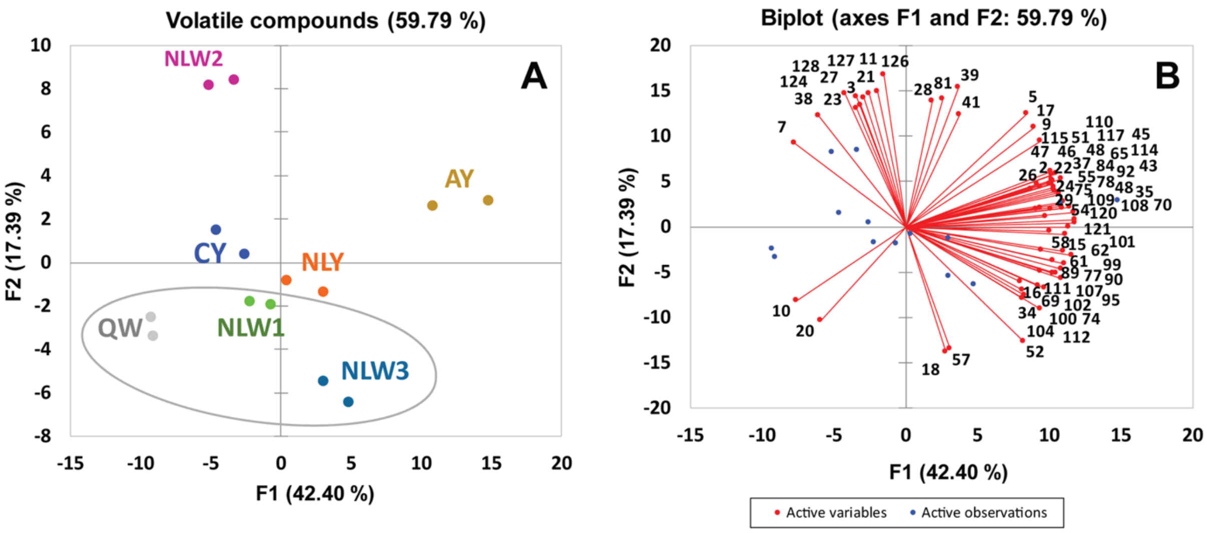

S1 Fig. Principal components analysis (PCA) of volatile compounds detected by headspace solid phase microextraction tandem mass spectrometry (HS-SPME-MS/MS) in white and golden variants of Newfoundland chanterelles. A, observations (sample clustering; ellipse highlighting the position of three of four samples of white chanterelles); and B, biplots showing loadings of chemical variables. Sample codes AY: *Cantharellus betularum* (golden); CY: *C. camphoratus* (golden); NLW1–3: *C. enelensis* (Newfoundland, white 1–3); NLY: *C. enelensis* (Newfoundland, golden); QW: *C. enelensis* (Québec, white). Numbers in panel B represent volatile compounds as listed in Table 4.

Supplemental Data Files:

a) Aligned fasta file of *Cantharellus* Al-1 genes, from GenBank
b) Aligned fasta file of *Cantharellus* Al-2 genes, from GenBank
c) Aligned fasta file of *Cantharellus* Al-1 genes: G=golden, W=white
d) Aligned fasta file of *Cantharellus* Al-2 genes: G=golden, W=white
e) Fasta file of deduced amino acid sequences from *Cantharellus* Al-1 genes: G=golden, W=white
f) Fasta file of deduced amino acid sequences from *Cantharellus* Al-2 genes: G=golden, W=white

Aligned fasta file of *Cantharellus* Al-1 genes, from GenBank
>C._cinnabarinus_Al-1_scaffold_1057_len=12158
GGGTAGCTTTTACAATCTGTCAGAGTCCGCGCGTCAGTTTGCACGTAAGAATCTCCC
CAGGAAACTAGTCATTGTAAAAGCTGGGGGGCCAACCCAGCTGGCAATAAGGCCTT
ATATACATGAGTTCCGCAACTCATAAGAGCACAGCTACATTGGGAACACATGGTAG
CCGATAGAGAAGCGACATGAAATAGGAGAATAGATCATGTGGATAAGGGGGCGAT
AACGGGTGAGGGATGACGTGATAGTGGAAATAGTGCATTGAATACAGCCTGCGTTC
TAACTACGGCAAATCACATGCAGATATCGGCTTACAGGTACTCAACCTGTCCGGATA
GGGGTCCGGGCCGGCCAGATAACCGTCCGTGCCCTGACAGTTGGAAGAGGACATGG
GCTGGGTTTTGTGATAATATGATATGGTTTCCTGTAGTTCCTCATCCAGAATTTGGC
AGTACAGGTCCCCATCCATCTTCCCATCAATCTTGCATGCCATTCCAACCCCATCCCA
GGTCATACAACCCCATACCATGATGGAATTTCCACCAAACTTTTTTGTGCCTTGCACC
AACCTGTCAATGAGAGGCTCCCCCACCCACTTCCACACCCATTTCCTCCCATCTGAG
CCCAGACGATTCACCTTAGTCTCATCAGACCATACAACTCTCTCCCAGTTCTCTAGA
GTCCACTCCTTGTGGCTGAGGGCAAAATCCATCCTGTCCCTCTTGTGACACCTGGTG
AGCATAGGCCTTTTTTTCTTCATCACTGCTTTCCAACCAGCTTTTTTGAGCCCTTTACA
TGAAGTCTAGGGATGTGTCATTGATGTTTCGGAGTATTCGGGTGACTTGGGATGAGT
TGTCAGCCTTCCCAGAGTTGGTGAGACAACAAGCATGGTGGATATCAGTGTCAGAA
AGCTTGGATGGACGACCACCACTGGGCTTGGGAACATCAGAATGGTGTTTAGATCAT
AGGGGAGTGACAGTGGAGTAATGGTGGCCAGTAAGAACTGAGATTTGGCAAGCACT
ATGGCCAGAATCAATGAGGGAAAGGATTTGTTCAACTTTTGAAGTGGCTAAAGGAT
GCATTTCGGATTTGTGGCCTTATCATGTGAAGTCCTTCGGTATTTATGCTTTATTAAT
GTCATTTTGTTTACTAGTGTGTACAGTAAACAATATCACATGACATGTCACATGATA
TTCCTATTTTCTGTTCAGCCTGGGGGTACTCCAATAAACCGTTGCAATGACCTGGGTA
GTTTGTAAATTACATAACATTACCAAAATTAAAAATATTGCTTACATAATTGGCAGA
TCTTTAGATGAGATACTTTCCCATGTTTTAAATATTTTTTCGAAATTTTCAAATACAT
TTTCATTGTGCTATAAGGTCATGAGTTGAAAAGTGAAAAACATTGCAACGGTTTATT
GGAGAACTGTAAGTACAACGAGAAATAAACAGGAATAAAGTAATAAACACTGCTCC
ACCATCCATTCCTTACAGACCCAAAGTTCTTAGCTCTGTGAGCTCTGAGAGCTCTCAT
ACCTCCTGGTTGAGTTTGCTGATGTAGGTCATGTCAGGGTCTAGGGTCCTACATTCCC
CCAAACTGGACATGCAGTGCCCATCAAGAATCCCGCTACAGCAAACAAGCAAGGAA
ACAAGGCTAACTATTGGCATAGTGGAGGTTTTCAAACTATTTTCAATGATAATATAC
ATTTGCAAATACATTTGAATCGAAAATTTTGACATGTGCAAAATAGGTTTAGTCCCT
CTGACCAGAGACTATCATACTAACATGGGGTTCAGCATTTCCCAAATGACCAGCCAA
ACCGAGTTCCCTGATGTCGTGAGACTAAGAATTATTCTCACAGGGGAGAAGAGATC
ATATGTCATTAATTCAGTGAATTGATAAGGCAGGTTCATGAAACAGTTCCGGATGTG

TGGCGAAAATCTGTGCAGGTGAATATGCTGAATCTCATTCTCAAATTGTGACAAGCA
AGAAAACTCTGGCATGAACTATTTTACAACGCATCGCGATATGCTTCATCAGGGCGG
AAATGAGAAGCATTTCTTTTGACAGACAAACAGATACAGGTTATTCATGTTCATCGT
AGTTGTCCCAAACAGCAACCTGGCCCGCCGACACCCATTGGATGTCCCCAAGAGCTT
TGGTTTCAAGAGCTAGAGAAGCAAGTGAGTACTACCGCACTGTCATGTCTTGTAGTG
GAGCTAAGACTCACGAAAGAACTAGTGAAAATGATGAGGGTGGGCGGAGAGCAAT
GTCATCAAACTTCACGACTACTGAGCGAGCAAAGGAAACACGTACAACGGCCGTCA
TGTCTTTACTTCCTTCGGGAGGGCAAAATAGGATTCGATCGTTGTTATAATCGACGA
CTGATTTAGTGTCCCTCTCGATCTTAATAATGTCGTTAGTGGACAAATTTCCCCAGAC
GGCTGTGGGGACACACTATTAACGCTGTTTCGTTCTATTCTACGAAAGACAAATGCT
CACAAATGTTTCTTCCGGTGGCGTACCCAGCAGGGAGGTCGAACGAAACTTGTTTAT
CGATGGAAGCGATGGTGATGTTGACTTCGCGTTCATTAGATCTCTTTTCGACAAATG
CAGTGCTCGTTGAGAGTTCGAGGGAATCCCAGGACCCCCCTTCGTGCTCCGTACCGT
TCACGAGCTGTTTGACAGTGCGCGCCTGGGTATTATTTACTCAGGCATGCGTCTAAA
ATACACGCAGACAGCCACCTACCGGATCTCTGCCCACAAACTGGAGGTAAAAGAGT
CATCAATATCATAAGAAAGAAGAGAGCCCAACGGGGAAAAGTACCTCAGCATAAA
AAGTGCCCTGATCCCGACGGTCGACAAAGAACTGAACGATGACAATTGCGTCCGCG
TCCTGCACAGACCAATCGCTGTGGATCACATTGTACCTCGCCTTCCCATTGATATCG
GCAATTTCATTGTAAAACAAGGTACCGACGAAATCTAGAACCCAAGCACGTCATGA
ATCTCAGTATCGAGTAGACCAACATGTCATTACCTCTGCCGTAGGAAACGTTGTCGA
CGAACTCCCTCAAGATCTCAAGGGCTTCGATGCGCACGCCCTTCTCATTGGCATCGC
TAGAGTAGAACCTGGCAGAGGCGCGAGCCCACCGGGGGTACATGTGGAAGCGCTCA
TTGTAGGCAGCCTTGTAGCTCTGTTTGGTGGTGGTAACAGTATAATCAGGCATTGAG
CTTTGTTGTTTTGGTGTTAGAGTGTGAGTAGCAGTGCAGGTGTGGTTCGGGCCTGGG
GTCTCTACGATGAATCCATGAGCTATTTATACACCGATCGGGTCAATAGAGGACCCA
TACCATGAGAGATCACCGGGAACAGCTATTAATTGAGTTACTTCATGGTTTTCATTA
CTTACTGCCACACCCAGACATGTCGTCTTTCTTTGGCTGCTTGTTTCCGCCGTCGACA
TCCTGCTTGCGTAGTAACAATCAGCGTACACCATATCCTAGGATTTCCGCTCGAAAT
TATTCTTCTAAACTAGCTGCGCTGTGAGCGCCTCAATCTTGCAGGGGTCATGAAGGC
GCCCATGTCCTTCGGTAAGGAAGACCCCATTCATTTACAATATATGGCGCGCAGTGG
TTGATCCCCCTTTTCTCTTTCAGTTCCTGCTGGCCGGTCCTTGTTCTTACACCATCACT
GTCTAGCACGCACGGTGGTTGGGATGACTTCAATTCAAATTATTGAATTGAACCCAA
CTAGGTAGGGACTCGAGCCAATACAACAGTGGTTTTCAGCATTTTAGAGCAAAAAC
GACGTACCGGATTTTGCCAGTATCCCACAAACATTTCCCCCAAGACCCCACGTTTGG
CAAACCTCCAGGACCATGATATTAATTCTACACTCACAGACTTCTCGCGGCGTTACA
TAAATACCCCTATTACGCACATATTATTCCTGATGGAAATGTGGCGCAGGGGGCACT
CTTGTCACCCACGTTGTTTCAACGGCTACTGCAGAGGCAAAATAGCATGCACGAGTC
GTATCCAGTCAATACTCGGGCAGAGTTCTAGAGGCTGGTCAATATGGTAACACTGCT
AGTACGCGTCAGAAGGGCCAGATGACGAAAGAACGTGGAGACGAGGGAAGCAAGG
CACATATCGGACGAACTCTCGCAGATTGAGAATTACACAGATACATAAGATCTGGA
GCTGTTGTCCCATCCAACAGCGTCAGTCTCAGATGAAATATCGTTACTCGTTAGTAA
TCAGGGCGGCAGCTGATACGTGCGGCTGCAGCCACACAGCTGCAGCCGCCCAACTG
GTCACGCGTAACTGGACACGCGTAGCATGCGTGATAGCATATCTGTGTTCCTTTCAA
TGCCGCGCGGGTATGAAAACGAGGGGCAATCATCGCCAAAGAAGGGTGGTTGAGCA
TGAGTACAACTTGAAGCCTCAGGTATTCTCAGGGAGACGGGATGGAATTGTCCAGC
GTCGTTAGAAGGGCCAGATGGCACAAGAACGTGAGGACGAGGAGACCGCGGCACA

TTTCGGAGGGATGCTTGCAGAGTTGAGAATTACACAGATGCAATTTCCATGCCTGAG
ATCTGGGGCTGTTGTCCCATCTGACAGTGTCAGTCTCCGATGAAAGTTCTCATTACTC
GTTGGTAATCGGGCAGCAGCTGATGAGACCTGTATTAACATAGGATGAATGGGAAT
ATTTCCGCGTAAAATATAAAGAATGTCCTAGGCCTGGGCTTATCGTCCACGACTGCT
TCAGGCGCCCAAACGAACAGCATGTGTGATAGCATGCTTGTCTTCCTTTCAATGGCG
TGGGGGGATAAAAACGAACTAGGGTTATTTATATGACGCCGCAATAAGTCTGTTAG
CTTGGCCATCGACGAGAAAGGGCGGTTGGGCATGAGTGTAGAATTAATATCATGGT
CCTGGATTACAGGAGATTTGCCAAACGTGCACCTGGAATACGGTCATGAGGTCTTGG
GGGAAATTTCTATGAGACACTGGAACAATTCGGTAAGTTGGGACGTCCTTTTTGCTG
GAAACCGGGCACTGTTGTATTAGCTCGTTACCTTTGAAAATAAAAAAGGAAGTTCCC
ACGGGTTGCCTTGAGATGATTTCAATTCAATAATTTGGTTTGAAATCATCCCAACCA
CCACAATCGAGCCGATGCTCAGCAATAGACAGTGGAACAGCCCGGCATAAACTGAA
GGAGAAAAGGGGGGATCAACCACCGCGCGCCATATATTGTAAATGAATGGGTTCTT
CCTTACCGAAGGACATGGGCGCCTTCGCGACCCCTGCAAGATTGAGGCGCTCACAG
CGCAGCCAGTTTAGAAGAATAATTTCGAGCGGAAATCCTAGGATATGGTGTACGCT
GATTGTTACTACGCAAGTAGGACGCCGATGGCGGAAGCAAAAGAAAGTCTACAGGG
TATGTTCCTGGTTATCTCTCATGGTATGGGTCCCCTATTCACCCAATCGGCGTATAAA
TAACTCATGGATTGATCCTAGAGAGGCTCGAACCACCTGCACCGCTACTCACATTCT
AACACCAAAACAACAAAGCTCAATGTGTGATTATCCTGTTACCACCACCAAACAGG
GCTACAAGGCGGCATACGAGGAACGACTCGACAAATATGGCTCGTCGACTGGCGCC
TTTGCTCTGTCCTTTGGCAATGCCGATGGCCACCAAGAGAAAGGCGTGCGCATCAAA
GCCCCTGAGATCTTGAAGGAGTTCGTCGGCGTC--GACGGCCTTCC-CTTCGGAA--
AAGGTAGTGTTGGTCTGCTTGATACTGAAAATTAA-
TGACGTGCTTGAGTTCTAGCTCCCGTCGGTACCTTGTTCTACCGCAACCTTGACGA-
TATCA--ATGGGAAGACGAGGTACAATGTGATCTACAGC----
GATTCCTCTGTGGAGAGCACGGACGCA---GCTGTCATCG-
TTCAGTTCTTTGGCAATTTTAAGAGTCAGGGCAACGTTAAGAGTCAGGGCAATTTTT
ATGCTGAGGTGCTTTTTTCTGTTGGACTCTCTTCTCTCTTATGATATTGA------------------
TGGCTCTCTTT--GCCTTCAGT-----TTGT-------------------CGG---CAGAGATCCGGT---------
AGGTGGCTGTCTGCATGTGTTTTAGACGCATGCCTGAGTGA-
ATAATACCCAGGCGCGCACTGTCAGACAGCTCGTGGACGGTACGAAGCACAAAGGA
GGGTCCTGGGATTCCCTCAAACTCACAACGGCCACTGCACTTGTCGAAAAGAAATCT
TCGCAACGCGCAATCA--AAATCAGCAGCGATTCCATCCGTAA---
ATACGCCGAGTTCCCCCTCCCTAGTGGGGTTGCCAAGAACAAAAACATTTGTGAGCA
TTTGTCTTTCGTAGAATAGAACGAAACATCGTTTA--TAG----TGTGT-------------
CCCCACAG-----ACGTC---TGGGGCAATCTGTTTACCAACCACATCAA----CAAGA-------
TCATGAAGGGCACTG--AATCAGTCGTCGATTATA-
ACAACGATCGAATCCTATTTTACCCTGACGGGAGGGGTGGGGGCCTGGCGGCCGTT
GTACGTGTTTCC--TTTGCCCACCCAGTAGTC--GTGAAGTCTGA--TGACA------
TTGCTCTCCGCCCACCCTCATCATTTTCACCA-----GTTCATTCGTGGGTCTTATC-----
TCCACTGCAAAACTTGATGCCGTACTCACTCCCTTCTCTAGCTATTGAAGCCAAAGC
TTCAGACGCGAAAGCTCTTGGAGTGGATGAAAAGTTG--
ATTCGTGGGTCTTAGCTCCACTACAAAACTTGATGCC---
GTACTCACTCCCTACTCTAGCT--ATTGAAGCCAAAGCTTCAGACGCTGGA----
GGCCTC--CGATTGGTGGCTGCTTGGCGCGACCAAGATAAACATGATGAT-----
GATTAACCTGTGTCTGTTTGTCTGTCAAAGGAG---CACTTCTCATCTCCGTCCT---------

---GATG-------------AAGGATATCACGA---
TGCATTGTAAAATAGTTCATGCCAGAGTTTTCTTGCCTGTTC-
ACAATTTGAGAATGAGATTCAG-CATATTCACCCCAACAGAT----
TTTCGCCACACATCTGGAACTGTTTCGCAAACCTGCCTTATCAAT-------
TCGGCGATGTGAATAGATGGACTATATGATCTTTTCTCCCCTGCGAGCCAC-
TGAGCAGATGGG-----ATCTCCACTG-
TTGGACATGTATTCATACCGACACCTTTATCATAAGAGTC------
TATCCATCGTTCGTCCGT---CCGCCTCTCCGG-TCATGGGTCTGAG-ACTCAGAGGCTC-
---TTGCTTGGATTCGAGTGTGTCGTCCAGT----
CAAGAGCAGAATGTCCCCTAGATACCAATCCACATATCCACTCACGGAACTTCCTGG
AGTGCCTCCTCAAGTGCATCCGCATCTGTTTCGCTCCAGCCAGGTCGAAAGTAGCCC
CATGGCGCGGTACTAATGTAGCTGGTATCAATCCCGTCAAACTTGATAACTGGACCA
AGGCATGTGTACCTCACACCCTTGCCTCCCCCTGCCTGGCGTGGGTGTCCTGTTACCC
GAGCCCTACCTTCGAACGGCGCATGCTCAATGACGTTATAGAATCCCGGCTGGTTGT
ACAACGGATCCTGCATTAACTTCACTTGAAGCGTCTTGAAGAGCATAGGCCCCAAAC
CAGTATTGTACCTGAAACCATGCAATGCATGCTTTTCCCGCACGTACGAAATCGGAT
ATTGACCCTGACGTCTCAACCAAGCGTCAAACGCTGTGAGTGAAATTGGAGAAAAG
AACGAGTTG---
CCCTTGACCCCAAATTTCCCAGGTTCGGCACGTCTCACGAGACCCAAAACCGCCATG
ACTGCACATAAGGTGCCTGTGCAATAGTCGGATATCGGCAAGCATGGAATGACTGG
TTCGTCCAATCCAGATGCTACACCTGTCTCCCATGCGACGCCGCTCAGCCCATCAGC
GATCTGCTGGTATCCTGGTCGGAGACTCCATGGAGCATTCTCTGCCTCACCACCATA
GCACGCTTCGTCCACGATGATATAAGGTTTTCCTCTTTTGTTTCCAAGCTCGGCAAGC
CGCTCATGCGAGAGACCAAGCCTGTTCATGGCCCCAGGTCTATACCCTGTTATTTGT
TT------GTCGTCGGCATTTCAGTTTTCAACCTCA--
TTGCATATGGTTGCATACCTTGCAAGAATATGTCTGCAGATGCCAGCAAATGACCCA
TTTGCTCTTGACCTTTCTTCGAGCGTAGATCAATGTGGACGGGGCGTTTATTTAGGTT
ATAATCAGGCTA-------GTCGATGTACAAGTTAGATACAGCTAAACCAACAGAG-
GCGCCACAAACTCACTTGGAAAATTGAGAAGTCGGGCAGGTCTGGTCCTATGACCCT
CACCACATCAGCACCTAACTCTGCGAGCAAGCGTCCGCCGACGCTGCCTGCTATGAC
CCGGGTGAGTTCCACAACGCGGATGCCCTCTAGAGGTCTAGAATTCGTTGATGGTAG
TCCCGTTAAGGGAGAAGGTCCAGGGTTGGAGCTCTCTATATCAATGAATCTCGCAAG
CGGAAGAGAGTTGATGATCTTTCCCTGATGAAATATGAGTT-
TATAAACGCAAGTGATTGTTTAGAAACACTGGGCCCTTACGTGGTCGGAATTGTCAA
ACTCCGCCGGCTGCAGTACAATCTCGCCGACTATACCATTAGAATGACACAGCATTC
TAAGTTCCTCAGCAGTATACTTCAAGCATTCTTTCCCGATGACCTCCCTCGCTTCCTC
GATGTCTTTGACGACAAGGTTAGGATCGAGCCCGATGAGTTTAAGCATACTCGATGC
ATTCAGGGAGCAATGCGTGTGGAAGAAACGCCCGTCCTTAGTCTTGTAAACCTAGGT
-TCCCTTCGA-TCCCATGTTCGCGGTACAACATAT-----------
TACAACTTACGTTCATAATTTCTTGACGAAGCAAAGTGGTCTCTTCTTCATTTACATT
CGGTATGAGGCTCAGGTAGGAAGGTGAGGAGACAGGATGCCCATCCAAGGTGCTCA
TAGCAAAACCAGCAAGATACAAAGAGGCATGATCCGTGTTGATGAAGATTTGGGGC
ACTTTGGTAGCTCCGCGAAGCTGAAGAATGCGAGCTGCCGCGATGGCTAGTAGACC
ATGCAGAGCCGAAGATGCGGCTTGCTGACAACATTTGAAGTGAGAAATGCTGTTTTT
CCAGTCCAAGCTCGGATTGTAAACGGAGGGACTAACGCTTTTATGGGGCAACGGAA
GGAAGGGTTCGCCTCCATCGAAAGAGACGTTCGATCTAAGATCCGCTAGTTCCTTTG

GCAGCCCGAGTTCGTGAACGAGGTTATCCAGTATCTTTGCGGCATCGATATGGACAG
CAGGGACACCGTTATCAGCGATATTCATGAGAAATGTAGCTTGCTTCTCCCAGCGCT
AGTTTCAAATGCTGCATCGTGTGANNNNNNNNNNNNNNNNNNNNNNNNNNNNNNNN
NNNNNNNNNNNNNNNNNNNNNNNNNNNNNNNNNNNNNNNNNNNNNNNNNNNNNNNN
NNNNNNNNNNNNNNNNNNNNNNNNNNNNNNNNNNNNNNNNNNNNNNNNNNNNNNNN
NNNNNNNNNNNNNNNNNNNNNNNNNNNNNNNNNNNNNNNNNNNNNNNNGCAGCCCAC
TTAGAAGTCACTCTGGAGCCGTCTTAATGCGCCGTGGTACTAGGTGGTACTACAAAT
GTAAAATATCGTTAAGT-TTTAAATCAGGACCGCACCCCCAGTCTG-
CATATACAACCCCACTGACATTATGAACCTGAATTGAACGCCANNNNNNNNNNNNN
NNNNNNNNNNNNNNNNNNNNNNNNNNNNNNCCCGTCTTTCAAAAAAAAATGCGCGT
TGAATAATCAGGACACCATTTGTTATTCTTGATTAAATAAGCGAGACCGAAGGAGCT
CGAGAATCATTATCTCGGAGGTACCTGACGTCGATCCGATCCGAAGCCCGGCGTCA
ATAAAGTTTACATGCCACCCAAAGCTTGAAACTGGGCAGGCAAACGGCGTCGCGTA
TGAAGAAATGCCTTGCGCTTCTTGATCCCTTCTTTGATGGACCTCGTGCCTACCAGAG
GGGTTTTTTTTTTTCCGATGCGGCGACGGAAGAATCAACTTGATACTTTAAGACTG
AT-
TACCAACGTCATGCTTAAAAGGCAATCGATCGTAAGTCCTACGTGATTGCGCGTAAC
CTCAATAAATAAAAAATTTCCTCGCGCAACCGCGATATCTACGTAAAT----
ACAGTAC---AAACTCCCAACATGAGCTCATACT--TAGT----
AACAAAGGGCCTTCACACGACTCCCCCTTCCTAATGGTA--
TTTTAAAGGTAGCGCCATCCCTTGTACACACCGAAAAGAGTCACAGAAACCCAAAA
GAATATTATCGAGACGATTATAAGCCAGATTAGTAGCCTAAAAAGCTCTACGAACG
CCGTGCGCTTCGATTTAGG--CCCGGATACTACT-
TTGTTGTGCCCTACGGCATGACCATTCCCTTGAAGGGATTTGAATCCTGAGGTC---
TCATTAGGGGGAAGATGATTCGATAGGATTTGTTCCGCGGTTATCTTGGCTCCAGCG
AGGACGACAGGTACGCCAGTGCCT--TGGAAA----
AAAAAAAAAAGAGTAAAGATCATGAGTTAGCTGGGAGGGAAA--G-------GTG-------
TG--------
TTGTATTTACCTGGATGAGTGCTAGCTCCGACAAAGTACATATTCTTGAACTGAGAA
TGCTGCGTGCAAGGACGGAAACAGAGTGTATTGCT-AGCA-------
GAATGAATGGGTTTG---
AGCAGGGCTGCTGGTGCTGCCGATGTTGCAAGATGACTCACAAAAGGTCGAGTGAG
AGTCCAAGAGCAGCACCACGATCGAGGTTGAACTTATTTTTCCCTAAA-A--------
ACGACGGTGTG---------AGTGTATCTCAGCTATACC------------ACAAGTTAA---
CTTATTACTTACATACGATTGGGTTATTCACTATCTCATGTTTAATCCATCCATCCTT
ACTAAGATCGATCCCCATCCTAGTTTTGATAGTTTCCAAAACTTGCATTCTTGCACGG
TTCACCAGTGCATTCACCCTATTTCCTTCCTCAGAGTCCACCTCCCTCTGCAGAAGAT
GTCCAACAGGCACCAACACCACAACGGTATCGCAACCACCAGGTGCAGCAGTGTCA
TCGAGCATACTAGGCACATTTATATAAAAACTTGGATCGGTTGGAAGTGTATGCTCT
TTGAAGATTTGATCGAAAGATCGCTTGTAAGCGTCTGCGAGGAAAATGTTGTGGGCG
GAGAAGTAGGAGTAGTCGTCATTCTTCCCCTCGGCAGGCGAAGAAAAATTTGATGCT
GCTGGTGCGTGTTTAGGACGGCGTGGGAGAGGTCGGGAAAGAGACCAGTAGAAGG--
AAATGGTTGAGCACGATGCAGTTCTATTTAACACGTTCCTCCTAGAAGAGGAAAGG
GGATTGAGATCATCGAGGAAACCGAAGATCTTGCCGAGGGACGATAACCGGGGAGA
TGGGAGAAGTTTATCATAAGCGTAAACGAGATCTGCATTACAGACCACAATATCTGC
TTCCAAAGATTCGCCGGACGCTGTAACAACGCCTGATACTCTCTTTCCGTCATCAGA

GAATAACAGTGACTTGACGGGGGATGACAGGCGGATGGTAGCCCCGAATCTTTGAG
CGATCTCAGCCAGTTTGATGGGAACGATGGCAAAACCACCGATAGGGTACCATATT
CCTCGGGCGAATTCGGTGTATTGCAATAATGAATAAGTGGCTGGTGCATCGAATGGT
GATATTCCGGTGTACATGGAGGCGAATGTGAACGTTTTGCGCATTTGAGGCGAAGCG
AAATATTTGCAGGCACGGGAATACTTTATAGCACAACCAGAAAAGGTAAGTAAAGC
GGGATATCGGGTGGGGATCAACTCCAATGGTGTGACC-
TACCAGAGATTGGAAAGGGTGAAGGTTGAGGAGATTGCTTAGGACCTCTGGGCGGA
GTAAAGACAGGATGGAGGTGTAGTTTTGATGCATTACGTGTTCCAGAGAAACTTCGT
AGTGACGATGGGACTCCTTTAGAAAGTCGAGGAATCTGAAGATAAAGTTAGACAAG
TG---
AGCTCTCGATCGAATCACTCGGGTTCAATAGTATACCTCTCAAAGCCATCAACACCT
TCCCACTTCTCGATTTGGTCCTTCATCAAGGACATGTCCGTGGACAGGGACACACTA
TCTCCACTGTGGA---------------AAT-GGAGGGTGTAATTAGGTTCGCATTTTAC-----------
----

>C._cibarius_Al-1_scaffold15338_len=1644

CACCAGGTGCAGCAGTGTCATCAAGCATACTAGGCACATTTATATAAAAACTTGGAT
CGGTTGGAAGTGTATGCTCTTTGAAGATTTGATCGAAAGATCGCTTGTAAGCGTCTG
CGAGGAAAATGTTGTGGGCGGAGAAGTAGGAGTAGTCGTCATTCTTCCCCTCGGCA
GGCGAAGAAAAATTTGATGCTGCTGGGGCGTGTTTAGGACGGCGTGGGAGAGGTCG
GGAAAGAGACCAGTAGAAGNNNNNNNNNNNNNNNNNNNNNNNNNNNNNNNNNNNNN

NNNNNNNNNNNNNNNNNNNNNNNNNNNNNNNNNNNNNNNNNNNNNNNNNNNNNNNNN
NNNNNNNNNNNNNNNNNNNNNNNNNNNNNNNNNNNNNNNNNNNNNNNNNNNNNNNNN
NNNNNNNNNNNNNNNNNNNNNNNNNNNNNNNNNNNNNNNNNNNNNNNNNNNNNNNNN
NNNNNNNNNNNNNNNNNNNNNNNNNNNNNNNNNNNNNNNNNNNNNNNNNNNNNNNNN
GATGACAGACGGATGGTAGCCCCGAATCTTTGAGCGATCTCGGCCAGTTTGATGGG
AACGATGGCAAAACCACCGATAGGGTACCATATCCCTCGGGCGAATTCGGTGTATT
GCAATAATGAATAAGTGGCTGGTGCATCGAATGGTGATATTCCGGTGTACATGGAG
GCGAATGTGAACGTTTTGCGCATTTGAGGCGAAGTGAAATATTTGCAGGCACGGGA
ATACTTTATAGCACAACCAGAAAAGGTAAGTAAAGCGGGATATCGGGTGGGGATCA
ACTCCAATGGTGTGACC-
TACCAGAGATTGGAAAGGGTGAAGGTTGAGGAGATTGCTTAGGACCTCTGGGCGGA
GTAAAGACAGGATGGAGGTGTAGTTCTGATGCATTACGTGTTCCAGAGAAACTTCGT
AGTGACGATGGGACTCCTTTAGAAAGTCGAGGAATCTGAAGATAAAGTTAGACAGG
TA---
AGCTCTCGATCGAATCACTCGGGTTCAATAGTATACCTCTCAAAGCCATCGACACCT
TCCCACTTCTCGATTTGGTCCTTCATCGAGGACATGTCCGTGGACAGGGACACACTA
TCTCCACTGTGGANNNNNNNNNNNNNNNNNNNNNNNNNNNNNNNNNNNNNNNNNNNN
NNNNNNNNNNNNNNNNNNNNNNNNNNNNNNNNNNNNNNNNNNNNNNNNNNNNNNNNN
NNNNNNNNNNNNNNNNNNNNNNNNNNNNNNNNNNNNNNNNNNNNNNNNNNNNNNNNN
NNNNNNNNNNNNNNNNNNNNNNNNNNNNNNNNNNNNNNNNNNNNNNNNNNNNNNNNN
NNNNNNNNNNNNNNNNNNNNNNNNNNNNNNNNNNNNNNNNNNNNNNGAATGAGAGAG
CATCGCCCGCCTATAAAATCATTCTTCTCGAGCAGAGTGACTTTGAGACCAGCTTTA
GCGAGTCGTGCGGCAAGGGCAACACCTCCTACACCGGCGCCTGTCGTGACGTGTAT
GCTATTATATGAGGACTCGGGCGAGCGCACAAAATGGCACCACTTGAACTGACCAA
TGATTATGGCACTTTTAGCGCCCTTGCCGTCGCCCA----
TATACGAAGCGCTTATGTGTACGGGCGAAGAGTTTTCCAAGTGATCAAGAAAAGCT
GGGGATAGATAAGCAAAC----TAGGGGGAGGAGAACAC--

>C._appalachiensis_Al-1_scaffold4647_len=8950bp

ATATGGGGGCCCCGGAAGTGATTCCGCTATTGAATCAAATTTGTATATTATTACTT
AATGCCACTATTGAATTCAATTTGTAGAGAACATTCCGCAATTGAATCTGATTTGTA
TTGCTAGTTACAGTAGTGACAGCACCGCAAGTGTAGGCAGATTATTGATGACAACTC
CAGATTTTTCATGACAATTCACTATCCCAAAGACTCGCATCCAAAAAAGTATTTTGC
ATTTTTCTCAATAATGTACTAGTCAATTTGGTACCTAATTATATATTTTGGAAGCTAT
TGCATGTGGCTATCCAATGATGTAAAGTGTGTTGACACCATGGGAAACAGCCCTGAC
ACCCCTTTGGACAGTGGTGAGCATTGTGCACAGACACCGAGTTGGACAGCCACAGC
CACCCGGTATCCCAAAAAGACTCGTGTCCAAAAAAGTATTTTGCGTTTTTCTCAATA
ATTTACTGGTCAATTTAGTAGCTAATGATATATTTCGGAAGCTATTCAAATTGGTGC
AAAGTATGTTGACACCGTGGGAAACAGTCCGGACACCCCTCTTGACAGCGGCCAAC
AATGTGCACAGACAATCATTTATACAATACACATAATAATCATAATATTAATAACTG
GTGGACGCTGCCTGGCGCGGCAGNNNNNNNNNNNNNNACGTGTAACCGTTATGTAA
AATAACAAAAGAAGGAACTACCCGATAGCTAGGCGATTTGTAAGCGATTCAAAACC
CTAAAGGAGAAAACTCAATAGAGGGAATTTCATTACTTTCAGCGTCAGTTCCCACGA
ATTTCAGACCTGCAGTGACCTGACATGCAGGTAATTAATATATATAACAATATAATG
CGGACAACTCCGCTACAATTCAGTGGGGGGCCCCCAAAATGAATAGGGGGCCCCCA
ATGGCCTGTTGGGTAGGCTTGGGAACGCAAAATGGACACCTTATTGGGTCCCACCCC
CGGCTGAGCAAGGTGCATATTTGCCTTTACTCGGAAGTTTTATCCTCCTCCCTCAGCA
TCTCTATATCGCGTGCTCCTCCCCTGCTCCACCTTTGTACGGTTCGTCCTTA-----
GATAGTTCTAGTAACTAGTAGATAGATGGAATTCAATAACGTACAGACTAGTACTAA
TCTTGCCACACATCTTATGACTCCGTCTTATCGGTTCAACGATGTCAATCTTGCACCA
GCACTACCAACTCTTCAACCATTCGTAATCTCTGAAAGTCTTCGATCCATTCAGTATC
ATGTTCCGATTCTTCTGAGCAAGCGAGACGATTAAAGGACACAGACATCTTATTGCT
TACCTCGCAGACGCGCGCGATGGTGTTCGGGAGTTATTTATTTCCCGGTTATCGCGT
GCAATAACCCACTTTCTATAACTTACTTCCGAGATCGACATTTTACTGCTGCTCCTCG
GCCGTCCTCGAGCAATCTGGTGGGTTCCTGTGCCCCGGCATGACCTTTTCCAAGAAG
TTACATAACCAGTGACGATGCCTGCGGGAAGCCCGGCAGGTATCTCCGATGAGTGCT
GACCATCTGTCAACATGCTTAGAAAGGCTCACATTTCGACGACTTGTCCAAATTGTA
ATTATTATTGATTTCTCGGAAGGGGCATGAAATACACGATATTCTCCTGTGTAGTCA
CTGTCCGCAGGAGGGGTATCCGCAGTCAGAGCCACAGGTCTGCTGGCCCTCTCGCTC
AAATTCAATTTCTGGATCCGCAGAATGACCTATGGACACATCTCCCCCGTTCCACTC
ATGATATCCCTCGGAGTGCCTCTTCGAGCATATCCGCATCCATTTCGCTCCAACCAG
GTCGAAAGTAACCCCATGGTGCGGTACTAGTATACCTAGTATCGATCCCATCAAACT
TGATAACTGGACCAAGGCATGTGTACTTCACACCCTTGCCTTCCCCTGCCCGACGTG
GTTGTCCAGTTACTCGAGCCCTACCTTCGAAAGGTGCGTGTTCAATAACGTTATAGA
ACCCTGGCTGGTTGTACGATGGATCCTCGGTTAACTTCACTTGAAGCGTCTTGAAAA
GCATAGGCCCCAAACCCGTGTTGTATCTGAAACCGTGCAATGCATGCTTTTCCCGCA
CGTACGAAATCGGATATTGACCTTGACGTCTCAACCAAGCGTCGAACGCTGTGAGTG
CAACTGGAGAAAAGAACGAGTTGCCACCATTGACCCCAAATTTCCCAGGTTCAGCA
CGTCTCACGAGACCCAAAACCGCCATGACTGCACATAAGGTGCCTGTGGAATAATC
GGATATTGGCAAGCATGGAATGACCGGCTCGTCCAATCCACATGCTGTACCAGTCTC
CCATGCGACGCCGCTCAGCCCATCGGCGATCTGTTGGTATCCTGGTCGGAGACTCCA

AGGCGCGTTCTCCATCTCGCCACCGTAGCATGCCTCGTCCACGATGATATAGGGTTT
GCCTCTTTTGTCACCAAGCTTGGCGAGTCGTTCGTGTGAAAGACCAAGTTTATCCAT
AGCACCAGGCCTGTACCCTATTTTTATTTTTATTTAATCGTCACCGTTTCGGTTTTCA
ACCTTAAGTATGTTAGATTTGCGTACCTTGCAAGAATATATCTGCAGTAGACAGCAA
GTGGCTCATTTGTTCCTGACCTTTCTTTGAGCGTAGGTCAATGTGGACAGGACGTTTA
TTTAGGTTATAATCAGGCTAAGTTGGCGTATTTTTTTTAGTTGAATACACC-
AAACCAACAGAGGGCACCATAGACTGACTTGGAGAGTTGACAAGTCTGGCAGGTGA
GGCCCTATAACCCTCACCACATCAGCGCCTAATTCTGCGAGCAAGCGTCCGCCAACG
CTGCCTGCTATGATCCGGGTGAGTTCCACGACACGGACGCCCTCTAAAGGTCTGGAA
GTCATCGATGGCAATTCTGGTAGGGGAGAAGGTTCGGGGTTTGAACCCTCTATATCA
ACGAATCTCGCAAGCGGAAGAGAGTTGATAATCTTTCCCTGATGAGATAAGAGTTAT
AAACTCCCAAGTATTCGCTTAGAAACACT-
ACCTCTTACATGGTCGGAATTGTCAAACTCCACTGGCTGGAGTATAATATCGCCGAC
GATGCTGTTAGAATGACACAGCACCCTAAGTTCCTCGGCCGTATACTTCAAGCACTC
TTTCCCGATGACCTGCCTCGCTTCCTCGATATCTTTCACAATGAGGTCAGAGTCGAGC
CCTATGAGTTTAAGCGTACTCGATGCATTCATGGAGCTATGCATGTGGAAGAAACGT
CCATCTTGAGTTTTGTACACCTAGGTTTGCGTTCGACCCCCATGTTCAC---
ACAACATATACAAACCTAAGCACGACTCACATTCATAGTTTCTTGACGAAGCAAAGT
GGTTGGTTCTTCGTTTACATCAGGTACGAGGTTCAGGTAGGAAGGAGAGGAGATGG
GATGCCCATCCAAAGTGCTCAGAGCAACACCAGCAAGGTACAGGGAGGCATGATCG
GTGTTGATGAAGATTGGGGGTGCTTTG---
GCACCACGAAGTTGAAGAACGCGAGCTGCCGCGATTCCGAGTAAACCATGCAAGGC
CGAAGAGGCCGCTTGCTAACAAAGGTCCAAAGTAAGAAAATTCTGTCTCCAGTGTC
AGGTTGTTTTTTAAAACCGAGAGCTAACACATTTATGAGGCAACGGAAGGAAGGGT
GCACCTCCATCGAAAGAGATGTTTGATCTAAGATCGGCTACTTCCTTCGGCAGGTCG
AGTTCGTGAATGAGGTTATCCAGTATTTTTGCGGCATCGACATGAACAGGAAGGACA
TCGATATGAGCGATGTCCATG----------
CTTGCTTTCCCTAGCGTTTGTCTCAAATGCTG----GGGAGATC-------------------------------
----
GATCATCCCACCGTGTGAACTGAAGAGTCAAGTCCATTGAGGATTCATTGACGCTGT
CCGTATTACAATTGGCTATAGAGCCATGCATACAGTAATGCAGTTCTAGCATGTAGT
CCATTCTTCTGTCCAGCTGTTGGGCTGTTGGGCTGTTGGTAGTCCCTACTCATGCG-
CTTCA-GGGCTGAGATA-----
AGGGTCGCCAAGTGTCGCCAGTGCCTGGGTGTAGGAAACAGTATCGCCGAAGAACT
GGGTCCCATATGCCCCCTGAGACCCCTGCTTCCAGCATTTCAC-----------------------------
------------CGATCCTCTCTTGGACAA--------GCTTAGAATACT-
ATAGCTCTACTCTTTCAAGTTGACCAAGT--GCCAAGCCACA----------
AATCAATCAATCGGAAATACC---CGTCTTTCAGATTGCA---
CTTGCTAACCGGGAATCTCGATCCGACGTAAAGCC---
ACCGAAGCCGGTACCCACATTTACATATGCAAACAT-------
GTATGAAAAGATCTTCTTTGATGCACCT----------------
TTCGATGTGCTTTCGAAGAGGAATCAGTTGAATT------
TTTTTTTAGGACTATTGTGGTAAC---------ACGATGCCATGCATGG--
CTTACTACGGGCTTGCCC--ATCGTGCGTCTATATACAACG--
CTCCAAAATCTCCGACATCCACGTACATAGAAACAGTGCGCAAAACCCTCATCATG
AGCTCATACTACTACTGCTGATATTAAGGCCTTC-----

ACGGCTGTTAACGAATAGAACCTCCCACAAAGAGAGCCCGCCCTCATACACACCGA
GAAGACTCACAGAAGCCCAAAAGAACA---TCGAAACGATCATCAGCCAGAT---
CACCTTTAAAGGCTCGACTCCTCTGGACGTTTTCGGCTTGGGTACCAGTTTGTTATTG
TTGTTGTACCTCGCAGCATGACCATTCCTTTGGACGGATTTGAATTCTGAGATCTTAT
CGTTAGGGGGAAAGTGATTCGATAGGATTTGATCCGCGGTTATTTTGGCTCCAGCGA
GGACGACGGGTACGCCTGTGCCTTTTGGAAAAAAGAAAAGAAAGAAAGAAAAGAT
CATGAGACACATTTATGGGAAAAAAAAAAAAAAAGTGGGATGTTCGACGACTTTTTTTT
TTTACCTGGATGAGTGCTAGCTCCGACAAAGTACATATTCTTGAATTGGGAATGCTG
CGTGCAAGGACGGAAACACAGTGTGTTGCTGAGGAATGTTGTGAAGAGATTTTTTTG
ATTAGCACGAGTTAAACGGTTGCCTTCAGGGGGGGAAGACTTGCAGAAAGTTGAGT
GAGAGTCCAAGAGCTGCACCACGGTCGAGGTTGAACTTATTTTTCCCTTAAAAGTGG
GGGAACGACGGTGTGTTAAGTACTAGTGCA-
ATCAGCTATAACCGTGGGGGGGGGGGGGGTTAAACCCGATAAACTCACATACGATA
GGGTTATTCACTATCTCGTGCTTGATCCAACCATCCTTACTGAGATCGATCCCCATCC
TTGCATGGATGGTTTCGAAAACTTGGTTTCTTGCACGGTTCACCATAGTATTCACTGT
GGTTTGTACCTCGGAGTCCACCTCCCGCCGTGTGAGATGTCCAACAGGCACCAACAC
GACAATGGTGTCACAACCACTGGGCGCAGCAGTTTCGTCAAGGATACTAGGCACAT
TTACATAAAAGCTTGGATCAGTGGGAAGAGCGTGTTCCTTGAAGATTTGATCGAAGG
ATGGCTTGTAAGCTTCTGCGAGGAAAATGTTGTGCGCGGAGAAGTA--------C----
TTCTCCCCCTCG---GGTGAGG---AATCTGACGTTACTC---
CTTCTTTAGGGCGGCGCGGGAGGGATCGGGAAAGCGACCAGTAGAAGG--
AAATGGTCGAGCAGGATGCAGGTCTATTTAACACATTCCTCCTAGAGAAAGACAAG
GGATTGAGATCGTCAAAGAAACCGGAAATCTTGTCGAGGGACGATGGCGCGGGAGA
TGGAAGAAGCTTGTCATAAGCGTAAACGAGATCTGCATTGCAAACCACAATGTCTG
CGTTGATGGTTTCGCCGGACGCGGTGACCACCCCTGATACTCGCTTTTCATCCTTGGA
GATTAACAGTGACTTGACGGGAGTCGACAGGCGGAGGGTAGCCCCAAATCTCTGGG
CGATCTCTGCGAGTTTGATCGGGACCATGGCAAAACCTCCGATAGGGTACCATATAC
CTCGTGCGAACTCGGTGTATTGCAACAAGGAATAGGTTCCTGGCGCATCGAATGGCG
ACATTCCTACGTACATCGAGGCGAATGTAAACGCTTGGCGCATTTGAGAGGAAGTG
AAGTATTTGCAGACACGAGAATACTTTGATTCGCA-CCACAGCAGATAAGCAA-----
GATATTC---GAAGATCAACTTCATT---
GTGACCTTACCAGCGAATGGAAAGGATGAAGGCCAATAAAATTCCTAATGAGCGGT
GGGCCCAGCAGAGACAGAATGGAAGTGTAATTCTGATGCATTACGTGCTCGAGGGA
GACTTCATAGTGACGATGGGACTCCTTTAAAAAGTCGAGAAATCTGAAAAAAAAGA-
AAAGAATTAGCCAGATTGCAA--GATTGATT--GGGTCAA--GT--
ACCTCTCAAAACCATCGACACCTTCCCACTTCTCGATCTGGTCCTTCATCAGAGACA
TGTCCGTAGACGGGGTCACAATATCGCCATTGTAAA---------------AAT-
GGATTGTATAATTGGGCTCGCACTTGACGAGTTGGATACCTTCAGCCTCCATCGAAG
TACCGAGATCCTCGAATGTTTCTCTAAATAATTCTGGTATGAGCGTAAGCGACGGAC
CCTGGTCGAACCGCTAATCACACGGAACGAGTGTTCAGTGGATGGGAGAGCAACTT
GCATAGGGCAACAGGAATGCTACAAACAGACCCCAGAACTTACATGGCCGTCGTGA
TGAATGAGGGAACATCGCCCGCCGATGAAATCATTCTTCTCGAGCAGAGTGACCTTG
AGACCAGCTTTGGCGAGTCGTGCAGCGAGAGCAACACCTCCAACACCTGCACCTAT
CATGA------------TACATGAGTACTAGCCACAG---ACGACATGGC-----
CTCGACTCACCAATGATGATGGCGCTCTTAGCGTTCTTACCATCCCCCAGAGCCATG
TGAAGCG-TTATGTGTAAAGATGAAGGATTTTTGGTGGGATCGAGA-------

GGGAGATAAAAGCAGCCATTATATGGGAAGAATCGAACCGTTTGCCGCAGTCGGAA
ATGCAGTCGCTGATTTATTGTCAATGTGATTGCCGCAGATATCATGGCGACTTCCAA
ACTCGTCGTTTGCGGGATCGAACTCGCTTGTCATTAGAAAAGCCGCGGTGATCCGTT
GGAAAGTGACCAGACATATGGGGACTCGTTGAAATATAAACAGGTGTGAAGCACGG
GCTAGCACGGGCTAGCACGACCCTTTGCAGACGCTTCTGGAGGCCCGCCCGTTCTTT
CGATGAATACTACAGAATGCTGAGGTGAACTCCTAGTGGTTAAAGAGTACCTCATTT
TGGTAATAACGGGGGTAATGCACATTAAAGATAGTCTGGCTAGTATGAACCAGACT
AATGGCCCGGAGATGGACTCAAAAGTGGAAATAGCCCCGGCACAGTCGGCACGTCC
TCGTCGACACCGCCAAAAGGCACATTAATGTACCACATGGCCGCATCTTCAGTTCTC
CCACGCCGCACTTTTTAATTCCACGGAACAGAAAGGAGTAAATACGCCTAAATGGT
ATTTGCGTTGGAGTTGGAGGGTCGGGGAGTGGCGCCGACGGGGCAATGGTTTTTCCT
CATTGCAGAAAAGATTAGCCATCCATCTCCACGTCGGGTCAAGGTGTCAACGGCCG
AGCTTCAACCCAGTGAGGACAAGTATAATCAACTTCCTCTCAGAGATTTGGTCGTTT
TCTACGTTTCCACTTGGACCTTCTCAAAAGGGTTGTACATCCAGGGGCCTCTTGGCG
GAATATTGACATGCCAAAAACGGGTAGTTGAAACATGGGTCGCATCGGTGCATGCA
GATTCCTAGTCTGTACACTCGTGGAAGAAGCATCCACGATCCCGATCGGAGGTCCTG
GATCGACTTCCAAACAAATGTATCCTCTCATCATCGATACGTCATTAGAAATCTTGG
CCAAACAGCGTGCAATCTTTCACGCTATCTAGAAGGGTTTCGCATACTTTTTCGTCCC
GTGAAAAGATGGGCCAATCCTCTACTTTAGTAGAGGCTGGGATCCTGCCATGGCCCA
GCACTTGCACAGCTCGTGGAAAACGCCAGAAATTGTGCCAACTCAACGTGCTCTAC
GAGTGAAAGACCCCCCCCCCCCGCAAAGGCACGAAGAAATCGCTTTTCCAAGTAGA
ACGTGTGTTCACTGCTTTCTAGTTTTGTAAACGCGACCGTTCGCGTTCCATTCAGCAC
TCTATGCATTCGAACGCTGCCCAAAAAAAACATTTTATTACCAATCCGATACGTGGC
ATCCTTTTGGAGCATATGCTGTGGGCGGTACCCACACTGCCCGTGAGCCCAAGCGCC
TAACCACGACTGCGTGTCAGTTGAGGGCAGCGATACAAATGCGATGATAATGGAGC
GGCCTCAGTCTTCCAACCGACTGGACTCACGGTCGGAAAGGGGGCTTCAACGGAGG
TCCTAGGACTTGGGTCGGTTGAGAGACGACTCTAGGGCTGCACAAAGGCCAATACA
CATCGTGTGCGAAACGCGACGTGTCACAATACATATCACCCGTGATCTTGTCATTTA
GTTGCCAAATAGCTTGTGTGTGGGCAGACTAGCCGATCACGGGGAAGCCCCCCTTGT
ATTGACTTGCCATGCGCACCATTGCTACTCAACTAAGTACGAGTGCCTCCAGACATG
GTTGACACGTGTTTGAAATTCTTGATGGTTCCTCATTGCCCAGGTCCCGAATGTTTCA
TACACATCATTCGGCTAAGCATCCCAGCATCACTGTACGCTCGCACGTACGTACGCC
AAATGGAGGTGAATGACCCATCAGGATAGCCAGCTGCTGACCACGTTGTAACACAA
GAATCAGATCGCAAAGGATGTATTTGTCAGCAAAGAATGAAACGGCATTTGCATCA
TCTAGTTGTTAAAGGCAGGAACATGACTAGTGCTCTTGGAGTCAAGTGAAA

Aligned fasta file of *Cantharellus* Al-2 genes, from GenBank
>C._cinnabarinus_Al-2_scaffold_2474_len=32191

AGTACATCACACATTGCGATAGTTCTGACGCTTGTGGCACCTGGAAGAAACTCCAAA
CATTATTCCGTGCTGAAGGCCACAGTTCCGTTGCTGGACTTCGCCGTCAATTCAATTC
AATGGGCTATGACCCTAAGCTCTCAATGTGGGATTGGGTCACGAAGGTTGAAGACA
TGGTTCGTCGTCTTGCCGATAGCAATTCCCCCATGATCCCCATCGACATTGTAAACA
CCCTCATCCGTGCATCCATACACTACTCGCCCATTATGCCAGGGAGGTTACTCCATC
CCTGATGGTTATTCCAACACCCACTCCCTCATGGCACGCATACAGAGTCCAAAATGA
TATCACACACACACACACACACACACACACACACAGTACCCTCCCAACTTAGAGAACC
AGAACTGAACTGGATTGGAATCAGATCATAACAGTGTTGGAAGTTTAGGT--
GGTCACAAGACCTAAAAGTCTGGATGT-
TCTGAGAATTGAACTGCAGAACTCTGCATTGGTCCAAGCTGGCATGTTACCATTACA
CCAAACACCCACCATAGGCCAAGGTGGTTTCCAACAAACAG---
AACCACAGTTCGGTTCCAGTATGGTTCCATTCACAGTTCGGTCCCAGTTCGAACCAG
TTCTCTGAGAACTGGGGCACCCCTAGTGGAGATGTGGATCGGTATCTAGGAGACATT
CTGTTAA--
ACTGAGCAGCACGATACATCGATTAGTGATGTATCGGCTCTCAGTGCAAGTCTTGTT
CTTGTATACATCCAGTGTTAGTTCTATGCCACTGAAATAAAAAATGAGCAGTAGTCA
AAGCATGAAAACAAAGGCCCAAAGCAACATTGTGTTCACCATCGATCACCCCAAAA
GCGCCTCCTTCCCAAAGCTCTCAAAGCGTGAATGTACGGTCCGGGGACCGCCAGTG
GCATCGGGCTGTAACTTCTAACACATTCTGCTCTTCATGAGACGATACACTCGATGT
CAAGCAGATAGGATGGCGACGTTGTAAAAACTCAGGAGAGTGGGACAGACGGACG

ATGGATGGACGGTATAGATAATAGTGAATAAATGTGTCGAATTTCCA--
ACAGTGGAAGACCCATCTGCAGAACAG-
TGGCTCACAAGGGAGAAAAAGATCATACAGTCCATCGATTCACATCGCTGAATTAA
CAAGGCAGGTTTGT-
GAAACAGTTCTGGATGTGTGGCGAAATCTGCTATGGGTGAATTATGCTGAACCTCAT
TCTCAAATTGTGACAAGAAAGAAAACTCTGACATGAACTACTTTACAACAATATGCT
TCATCAAGGCGGGAATTCCTTGA----CAGACAAACAGACACAGGTCAATTA----
TCATCCCCATCGTAGTCGTCCCAAACAGCAACCTGGTCCGTCGACACCCATCGAAAG
TTTCCAAGAGCCGTTGCGTCTGAATCTTTGGTTTCAAGGGCTAGAGAAGCAAGTGAG
TAACCGCATCAAGTCTTGGAGTGGACTCGAGCTAAGACTCACGGAAGAACTAGTGA
AAAGGATGAGGGTGGGCCGAGAGCAATGTCATCAGACTTCACGAC-
TACTGGGCGAGTTTG-
GAAGGAAACACGTACAACGGCCGTCAGGTCTTTGCTCCCATTCTGAGGGTAAAATA
GAATTCGATCGTCGTTATAATTGACGACTGATTTAGTGCCCTTCTTGATCTTTTTGAT
GTCGTTGGTAGACAGATTTCCCCAGACGGCTGTGGGGACACACTATAAA----
GTTTCGTTCTATGCTACGAAAGGCAAATGCTCACAAATGTTTTTCCGGAGGCGTAC
CCAGCAGGGAGGTCGAACGAAACTTGTTTACCGAGAGTAGCGAGGGTGATATTGAC
TTCGTTTTCATTGCCTCTCTTCTCGACAAACGCAGTGCTCGTTGTGAGTTTAAGGGGA
TGCCACTCCCCTCCCCTGTGCGCCGTACCGTCCACGATCCACCAGACAGTGCGCGCC
TGGGTATTTTTTTTTACTCAGGCACGCGTCTAAAACACATGTAGACAGCCACCTACC
GGATCTCTGCCGACAAACTG-
AAGGCAAAAAGAGTCATCAATATCACAAGAAAGGAGAGGGTTCAATGGGGGAAAA
GTACCTCAGCATAAAAATTGCCCTGGCTCTTATTGTTGCCAAAGAATTGAACGATGA
CAGCCGCATCCTCGTTCTTCACAGAGGCATCGCTGTAGATCACATTGTACTTCGTCTT
CCCTCTGATATCGTTAATGTCGTTGTAGAACAATGTACCGACGGGATCTAAAACCCA
A-GCACGTCATTAA-
TTTCAGTATCAAGCAGACCAACATGCCACTACTACCTCTTCCGTAGGCAACGTTGTC
AACGAACTCCTTCAAGATCTCAGGGGCTTCGATGCGCACGCCTTGCTCTTCGTTGCC
ATCGGCATTGCTAAAGTAGAACAGAGCAGAGGCGCCAGCTGACCAGCCGTACTTTT
TGAGGCGTTCCTCGTATGCAGCCTTGTAGCCCTGGTTGGTCTTGGCAACAGTATAAC
CCATTGAGCTTTGCTGTCTTGGTGTTGGAGTGTGAGTAGCAGTGTAGGTGTAGTTCG
AGCCTGGGGTCTCTGGGATGAATCCATGAGCTATTTATACGCTGATTGGGTCAATAG
AGGCCCTGCGCCATGAGAGATCACCAGTAATGGCTATTAATTGAGTTGCTTCGCACT
TTTATTACTTACTGCCCCACACCTAGACATGTCGTCTTCAATACCATGTAGACTTTCT
TTGGCTGCTTGTTTCGGCCGTCGATGTCCTGCTTGCGTAGTGACAATCAGCGTACACC
ATACCCTAGGTTTTCTGCTCATTATTCTTCTAAACCAGCTGTGTTGTGAGTATCTCTA
CCTTGCAGTGGGTTGGCACGAAGGCATGACCTTCAGTAAGGAAGATCCCATTCATTT
ACAATATATGGCGCACGGAGATTGGTCCCCTTTCTCTTTCAGTTCATTCCGGACTGTG
AATTCCTTGTTCTTACACCATCAAAGTCTACTGCTGGGTATCCCCTCGATTGCACGGA
TGTTATGATTGAATTAAAATCATCTCAAGGCCACCCGTGGGAACTTACCTTTTATTTG
CGGCGTTACATAACTTCTCGTAGCGCTATATATAAATACCCTTAGTACCCCAGGCCC
TCGTTTCCCAGCACCCCCGCGCCATTGAAAGGAAGACAAATATTAATGCTATCACGC
ATGCTTCTATAGTTGGACACATGATGCAGCCATGGGCAGTGATAAGCCCTAGGACAT
TCAATGTCCACTTTATTAATTTCCACTTTTACGAGGCAATCTTCCCATTCACTCTCTTC
CTGCTGAAGAGAGGCTGGTCACCTGCCTACCCATTCGCCCTGCGTTGATACAAGCCC
CATCAGCTGCCCTGATTACCAACAAGTAATGATGATATCATCGAACTTTTATCGGAG

ACTGACGCTGCTGGATGGGACAACAGCCCCAGATCTGANNNNNNNNNNNNNNNNNN
NNNNNNNNNNNNNNNNNNNNNNNNNNNNNNNNNNNNNNNNNNNNNNNNNNNNNNNNNN
NNNNNNNNNNNNNNNNNNNNNNNNNNNNNNNNNNNNNNNNNNNNNNNNNNNNNNNNNN
NNNNNNNNNNNNNNNNNNNNNNNNNNNNNNNNNNNNNNNNNNNNNNNNNNNNNNNNNN
NNNNNNNNNNNNNNNNNNNNNNNNNNNNNNNNNNNNNNNNNNNNNNNNNNNNNNNNNN
NNNNNNNNNNNNNNNNNNNNNNNNNNNNNNAGCCATGCGTAGACGATAAGCCTTAG
GACATTCAACGTCCACTTTCTCTTTTACGCTGTAATCCTCTCATTCCTTAATATACGG
CTTATCAGCTGCCGCCCTGATTACTAATGAGTAACGATATTCATCGGGAGACTGATG
CCGTTGGACGGGACAGCAGCTCCGGATCTTAGGTAGAGTAAATGTATCTGTGTAATT
CTCAATCTGCGAGAGTTCGTCCAATATGTGCCGTGCCCCCCCTCCTCTTCATGCTCTT
TCATCATCTGGCCCTTCTGACGGTGCCGGGCAACCTCACCCCTACTGGCAGTGTCGC
CATATCATTTTCCAGGAAGGACGTCCCCTCAACCTCGGTGCTCCTTTATTCCATTTCG
TCTTCCTGAACTCTGTTAGGCGGGTGACCAGCTTCTAGAACTCTGCCTGAGTATTGA
CTGGGTGCAAGTCGTACACCCATGATTTCAATTAGACTTTATACATCTCGTCAGTTTG
CCCATCGATTTTATTCCTTGGACAACGGCTCCAGCGTGCTATTTTGCGTCTGCCACTG
CTGCTGAAACAACGTTCCAGGTGACAAGACTGGTGCCCCGCGCCACATGAATATTA
CTGGCGCTGCGTTGTTCTTGGCAAGCTGGATTACCCCCCAAAGTTTGCCTCATCTTGG
AGCTGCGCAAGGTACTTTCTATGAAGTGAGTAGACTCCGAGTCCGTGACTTTGTTGC
TCAATGCTTTCGGGGGGTAATTTGTGAATGATTGAAGAGTTGCTAGTGCCGGTGCAA
GATTCATATCGCTGAACTGATAAGATACGATCGTGAAGCAGCTCTGGATGTGTTGGC
AAAAATCTGCGAGTGAATATGTTGAATTCCATTCTCGAGCTGTGTACCTGCAAGAAA
ACATTGGCATGAACCATCTCAACGACGGCAAGGAATAGTACTTCAGGGACTTGACA
GGCAAGCAGTCACTGGTCAATCATTCGTCGTCCCAAATAGCAACCTGGTCCAAGGG
CTGGCGCATCTGCAGCTTTGGTTTCATTAGCTAGTAACACAAGAGTCGGAAGTGCAG
CATGGAGTCTTGCAGTGGAACCGAGACTCATGAATGAACTATGAGGGTGGACACAT
GATGTGAAATGAAAAGATGACGAATAATTAAGGTATAGAACAGTTCTAAAGGGGAT
GACAAAAATTAAGTAGTTGACAAGTCATTAATTTGAGAACGCTCTGTTTATCTGTCA
TTGACAACATTGACGCTGGTAGTTTGGGAAGAAAAGAGGAAAGTGGCAGAAAGGA
AAATTGCAGCACTCAGGATTCCTAAGTAGGAACCCACATAGTACTAACTGAGCGGT
GCTCAGCTTAACTACGCCTAGTTTAGGTTCTCATGCAAGCGACTTGATTGACAATGC
CTTCCCCCACTGTTTCACATTATCTTCACCCGCCTCACTCAGGCCCGGTGGCGTCTAT
GTACTACACAACAAAGCATCACCGTATGTTTGCTCTGCGCTGTACATGCAAAGCCAG
TTCTTGAAATTCAATGTGCATGCATGCAGTGCAGGTTTTGTTGCAAAGCAAAGCGAG
CAATCTAGTATGACGAGATTTGTTGGAGCGTGAAGGTGTAAAATGGTCGCAGTGGG
CAGTTTATCGATCTATTTTGAATGTGCTTGTTCTCAAGCTTCCATTGTGCAGTTTGCG
TTCGTGACTGTTTGTGTTGACGACCGATGTGTCCAGTTCAATTTCATTATAATTATTA
GGGAACTACGTTCCACCGCTGGAATGCCATACATCAGAACAACGTCCAGAGGCCAA
AGTCCGGGGTCGAAATGGAAGTGAAGGATGGATGAAGACCAGGGGGGCCGCCACG
TGGACTGGTAGTGGTCCCAACCGGTGAGAGCGAGGCTTAAGCGCGACAGAGGTGGA
CGCCATGGATGTTGAGGCGCAATCGGAGCGGGCAAGATTTGCGTCCGACGTGATGT
CAGTACGATGTCAAGTCGTCAAAGTCCACCCAAGGCCAGCGAATGAGGTGGACGCT
CGCAGCGAGAGTGAGGCTCGAGCATGATGGAGGTATACACCACGGATGTGAGGCAA
GTAAACGCGATCGGAATGTCGCTGGGCGCGCGGCAAGCCAATGAGAAAGCGAGCG
ACCAACCGGAGCGTTTCAATGTAACGTCTTTGCACATTGAACTGTTCCAGTGTATGT
GCCGTTGCAACGAAATCGATGCCGACAGATTGAGAGCACGAATAGCACGTATTGGG
AGTGACCCGCGACGATTTACTTCCCTGCGGAGAAATCGGCGAGCGATTGAGAGTGA

CCTTGCTCTGCAGGTACTTGAACATTCCGAGTTCCAGCTTGGCGCAATAAATCCGAA
GGCCTCGATGGCACCACGGGTTTGCAAATGGCAGCACTTGTACACGTCTTCATCAAG
CTTATCTTGAAGTTGGATAGTGTTCAGTCCCAACCTAAGCGGGCCGGTTCACACCTG
TGTTTCCGTCCTGCAACAAATCCAGTGACATAAACCAGTATAGTCAGAGCAAGTCTA
CAGCATCATCCAAGAGAAAAATAAAGATCATCTATAGTCATTGAGTTCCTTTCTAGC
ACATTAACCTGTAACGAGGGTAGACCAGGCGACCCAGACCCTTCTCCACTTCGGTAC
ACTAGGCCTTGTCAAACGGGTCTGAAGAGGAGTCATTGCAAAAATGTCATCCGCGTC
TAAAGTGTATTGTGTATCACCAATCTTCTTCTCTAACTGCCTTCCAATTTCCATGTAA
CTCTCGACCGCGACCCTCAAGCCCTTGCGTGCCGAGAAAATGGTCGGCATCTGATCT
ATCGCTGGTCGTGAGGTAAGGAAGAAGTCTTTCGCCATCTGAAGCAAGTGAATTCG
ATGAATCCGAGCTTCGTCAGGTGGGAAATGATATGGCGATTGTGTAGTCACGG---
GCTTCGCGTTCGATCCGTGATGGTCAACAGACTCGGAGCTTTCAGCTTTGGAGAAGT
TGCCGATCCACCATTCTCGCGGAACGTACACCCTCCCAGCCCGTTCGTCCACCCTAA
TATCGCGGGCGATATTTACGAATTGGAGGGCCATACCCATTTTGACAGCTGCGTCCA
GAATTTCCTCACGAGGACGAGGGGAATTGTAAGAAGTGGGCGCATAGTGGAAAATC
ATGTGGCAACACATCTTGGCGACTGTACCAGCAACCCGGTACGCATAAACTAAGAG
ACACGGAATGCTCCATGATT---------GAATTCGT-
GTATAACAAACGAGAAAGAACGTACGAATCAAATCCTCGTCGTTATGAATCAGAAT
TTCATTTGGTGGAGCACCCGCCTTCTCTGACTCTTTGAATATGAGGTCCGAGTCGAA
GCCGTCAAGGAGCTCCAATAGCGGTTCACGGAGTGGGTCCTCGACCAGCTTGTATTT
T----ATCG-----
AGGATCCAGAGCTTGTGCGGGGCAGCGCAAAGAAGGCCCGTCGCTGGGCAGTCGAC
AAGTTGAGCGGCTTCAGCAAATCATCGAAATGCGAGCCTTCCTGTCCGGCAGGTATC
CCCGAGGTGTACCATCGATCAATGAACGCTCGGACATGCTTCAGAATCTCATGACCT
TCCTGTCTCGTGCGGGCATCGTCATCGATCAAATCATCGATAACTCGGCAAAAGGCA
TACCTAGGCGCCGGAACTTAATCAGTCAGAATGACTAGGAACAACTGAAAGGC--
TCACAAATTGATCAATTCTATCCTTAAGGGACCTTGGAATATGCTGCTTGCGGTCCA
GAAACTTCTACTTTTTCCTTTGAGGATCAAACTGGTGTCCCGAAGGTTGTTGAGCCG
GTGAGTGATCACCTCATTATTCAAGTGCCTCGAAGTCACTGAGAATGATGCGATGAT
TGAGAATGGTGCCGAGAAGAAAGTGTGTGACGCGGTGGAAGGGAAGTCTATGGCGT
ATATGGTAAGGATCCCGTACGCTCTGTCAATGGCGATTTGACCAAAGACAACGAGG
AAATTCGTAATTAAAAAGAATACTGCTTCTCTGAATAAACACATCACGGGAGTGGG
ACGTTTCAGAAAACAGAGTGGCGAGAAAGGAGGCAAAAACGACGCACTCTACGTCG
AGATGGCCCCAAAGCTGAACATTAAATTTGGTGCCAGATTCGATAACCCAAGTACCC
CGTTGAAGAGCTGTCGCATCAAGAATCCACATATACGCAGTCGAGAGGAGAATAGG
TCCAAGCGAGGATAAGGTGGGCATGGCGATGATATGAAGTCCTGCTACGCTCCTTCG
AAACGG---TGGGTCCATA-CATTCA-----AAAACGAAGAAGCGAGGA-
TTATTCACCAGATAAACTGAAGGATGGGACACGCCCAAACGAGTATGAGACCCATA
TACATTCCTTGGCCTCCTCTCCAGGATAACCATGCACCGCTACCTGTTCCAGCAGCA
AACCATAATGAGCCAAGGCGATGGAGCACCGTATGCGATGTGGATGGGGGGTGTTC
ATGGGTGGGTATCAGGGCAGGATGTAACACGGGTTTCGAAAGGATGAGGTACAGAA
CTGAAGTGAAGCATGTTTGGATGGCAAAAAAAAAGAGTTCTTCAGCAGGAATGGAA
TAGAATGTCACGTTAAACGTCGCGTCTGGCGGGTATGACCAAACCTTTTGTG-
AAGTTGTAGATTTTATCAACAAGAGAACGCAGTGTCAGATCAAAAGTCGAGAACGT
ACGTTGCTTCGGATAAGATACGAATCCCATGGGATGGTGTACGCGGTAGCGATAAA
GACAAGGATGCCAACTCTGAAGCAGTCGATAGAAGTCCTCAATGGGAAGTAAAGTG

CCAAGAGCACAGAAAACGCAGGTATAGTCCAGCAGAGATGACTATT-
AAGCGTCCAAATTAGAT-----GGGCTCAA-----
TGTTAACGTGAAGCATGTCACCTACACCAAATCGTAATCCGGGATTTTCGTTAGAGA
CACCACGTCCAAGATGCGTTCCAT-AGCAGGTAGAAT---
AATGTGGGGATGCACGTTGCACGTCTGAATGATCGGAAGGGA-GCTTGA-ACTT-
GAC-CGCGCTTGCCAAGTGGCTCCGTGGCACCTGTGTTGGTAGGATGTATTGGAA-----
-----CTAACGAACAATG------TACCAATGACAG----
GGGGAAGATGGGAGGAAAGCTGACATTGGTGTTATAAAAATCTTGACATGCCATCT
TCATCACTCGCAGGGGTTCTGAATTGTTGATGGGTGTTGACATCCGGGTCGCGGTTT
CCGACAGC--ACATGAAAATGCAAGCATGA-TA------TCGGGTTCCATT------CCG--
AACCAAT------CATGCCTGTCAACGC-----
GTTCTTCTAAGAATGGAATCCGAATTTGATGA--ACGGATGCCATG-TAAAGG-------
TCACATGATTGATCTGATTTAGGAAAATGAAGGTCGATCATCATTTATTCGC-----
TACCATTTTTGGACAAT-CACA-TGATC-CG----CCCGGACATTCATCC--------
AAATCGGGCGCGCCAATTTTTTTTTG-------GCTTGCTGTAA--------TGACTTGACCG-
GGTCGACTCCGAATGAATGACCTTGCTGAATGA------CAGTC--TCAAAATGGGCAGA-
-GACACCG-GAAAAATGATTTGA-AGATTCCCTTCGACATTC----ATTGACTATAA--------
-----G-TTACGTAAGGTGCAGCCAACTATGTAACGGCCCAAAGGAACGTTTCC-
TTCTCTAGGGA--GAAATC--------GCT--CCATCAATTCCCAGCCTAGGCTGTG-
CCTCATA-TATTATCAACCGCACGTT-TCGCACCTGGTGGGACAA-
CAGTTCATCAGGCTGACGCACCCTGTGCTTG--
AAGTATACCCAGAGCTCTCATTACATTGCTGCCGTTCCCAAAAAATCGACCACCTTG
ATCTTATCGGGAAGGTGGCGAAATCATGGCGCTTTCTGTTGATACATA--
AGTAGTATAGTAGCGGCGGTTGCGGCATGAGGACGTGACCCCAT--
GTCCCGAGACTT-CTGAA--CTCAGCACGACTAG---
CACTGGGGTACAGAATAACAGTGCCAATC-TG--ATAG-AGAAAATA-
CATAACAGGACA-TCAGGCACCT---TGATCACCA-------ACTGCAAGACCGAAG-
TAATCACAACACA-----GCATCGA---ACAATCAAGAGG---CG----TGC-
AGCGGACACAGATGGGGGAC--AAATACATGTTATTGGAGCCTGAT-
GCCTAGGTGCTAGGCTGCAGCTGGCTGGCATTAGCG-AATCA-
ACAGTGCACGAAGGATCGTAAGCAGCAGGCGACT---GGCCAGAATG--
AAGGGGGCATGGAATACAACTCTTCGC------ACC------TCGATT-----AAATCTT--
AGCTAA------------TATAATATCCTATTTTACTTGACTAAACGTGTCGGTGTCCGC--
GCCGA-
GGGAGCTATGACTGATGATACGTTCCTGAGTCAGCCCGCACACCGTGAATCAGTTCT
TCCGTGGATGAGAC---AAAACTTTCTCA--
---GC---ACA----------------------------
GCTTAAGGGTACATCCCGGTCGACCT-TGGGATCGTGAGTCGAGGCTTT--
CTGTGAAGAGT-CTTCAG--ATTCAGA---TTTACTGGGGT-
ACTCATCGCCTTTGTGAGGACCGGCAAGCAGTTGTCGAACGATACAGGCACGCACT
CCTGAGGCGTGAGCAACTTCTCAGGGACATAGAGACCATGATATCGATATCTTTTGC
ACTTG--GCTCAAGATGTCTCCCCATAGTGACTGCGT----------------------GAATGC-----
AGG------GGGTTTCACGG--TACC-ATTGA-GTTTG--
CCGTCAAATGCCCCAAACAAGGAAACCGC-CTT---
CCGTGAGCCAGAGTTCTCGCCAACTAGGTGGTCGACCTGGTTGCCGGCCGAGTTTTT
TGAGAATCGCATGTGCCAGTGACATCTCTCGAGAATAGCAAGGCCTGATCTCAATAG

AATTAAAAATCGTCCAGCCTCTCTTTCGTCACAAGCAGGGTCGTGATAAGATCGAGG
ACCTGAACCTGCGAGAAGTTCCTAGCCGTGATCGAAGGCCCAAGGTGTCAGAATGT
TCCACAAAAAGACACGCAGCAATTACGCAACAAAGGCAAGAGTAACCGGAGTAGA
TCGTCAATAGAATAGGCACATACATCCGGAACCGGGGAGGGACAGAGAGGCTAGTA
ACGGCAGCGGGAACTGGACATGGTTCGAAATAACACATCCAAAACCTTCTGAATCG
GTTGCGGATTGACTATTATCAATGATTTATCATCGAGCAGCATTCTCATAATGGTAG
TATGACGAGTN-----------------
NNNNNNNNNNNNNNNNNNNNNNNNNNNNNNNNNNNNNNNNNNNNNNNNNNNNNNNNNN
NNNNNNNNNNNNNNNNNNNNNNNNNNNNNNNNNNNNNNNNNNNNNNNNNNNNNNNNNN
NNNNNNNNNNNNNNNNNNNNNNNNNNNNNNNNNNNNNNNNNNNNNNNNNNNNNNNNNN
NNNNNNNNNNNNNNNNNNNNNNNNNNNNNNNNNNNNNNNNNNNNNNNNNNNNNNNNNN
NNNNNNNNNNNNNNNNNNNNNNNNNNNNNNNNNNNNNNNNNNNNNNNNNNNNNNNNNN
NNNNNNNNNNNNCGCACGAGATGTCTGCCATGCGGATGATGTGATTCCCTCACGAT
TGGTTGAGAATGCCCTCGCAAGCGAAGAGGAAGACATATCTATCGCTTGAAACTCCT
AACACGCTATCCAATGTAACACCTCACACGTTCCATGTCTTGCATGTTTCGGACTAG
AAGACTGGCCATTGAAGTTCGTCACAAGCAGGAACTTGTTATGTAAGATCGGCGAC
CTGAACCCGCGGGAAGTCGCTATATAGGCGTGAAAGCTCAGGCTCAAGGTGTCAGA
ATGGTGTCCCACAAAAAGACATGCACGGCGCAACAAACGGAAGAGTAATCCGCAGC
CCGTGAGTGGTATCCCCTTTCCAGGTGAAATCGGCTTCTACTGACCACGAAAGCAGA
AAGGTCCTAAGCACCCTCGTCTGTAATATACAGAGCATGCTGTTGTAGACGTACTAG
CGCAGAAGTTGACGATGTCCACGAAAACAACCTCCCTGTTCCTTATATGCATAATTA
GACAGAAATCAATCTAGGGGTTATAGAGATAAGGAGAGTCAGATATGTATGAGGTC
GAGACCTGCATAAGTCTGGTTCCATGTAATTAATCGCTTGCCACAGTCCGAGAAGAT
AGCCGAATCGGGCTCCGATTGGGATATTATGTAATAGGATCTGATTTTTTACACAGG
AAAATACGTCATTGATCGATCCAGAAGGGCACTACTATGTACTGCAGCTAGAAGTC
AACCCGTGTTGCCGCGGATCAGGAACAAAGGGAATCAATGGGACCGTTTGGCGTGA
CAGCAGATATAGGCCGTGCCTCGCCGCGGGAGCTTATATAGGGCCTCAAAGTCGCC
AGAAAACTCGGTAGTCAGGGAAATGTGTACGTGCCCCACGGAAGTTGGGGGGACAA
GTTATTGAGGATGTATCTTAGACCGAGGAGCACAGATACCAATGTCATCCCCATGGA
TATGAGAACGCCGCCCTTGGATCAGGGATCAGTTTCCGACGACGGAATTCCGTATTA
AGGGACTACAGTAGGATAC---
AATATGGATCAGACCCACTCCCTAGTATGGCTGGTGTACTGGTATCAAAGTATAGAA
CCATCGTCGACGTACAGCACGTCGCACAGTAATCCCGCTAATATGGGGCCGCAAAG
AGGACGGAGTATGTTCCACAGCATTGTATACCAGGAAACGTTGTCAGCCGGGGTCG
TGTATATCCTAGCCCCTGAGAAGAGATGAGGCACGAGGAAATTGGCCTGCGGAAAG
CGTTGGGATCAGACAGAAAGTGCGCCCACAAAGGAAGCTACAGTCCTACTGACCCA
GGTCACCCACTCGCTCAGAATTGTCCAGAGCGTGAGTTCTATGATGTGCATACAAAG
CACGATGGGTACTAAAGTGGGGTTGTGTGTCCCTTGTTTATCATTGACCGCACGAAG
CACGGCCAAAAAGATAAGCCCAGAACAGACAACTTCGAAAATGAAGCATGCAACTT
GAAAATACGCAGTCAGCTTTGCAGGATGAACCGTGACGATGCGGGCGGAGGAATAG
AAAACGCTCACTGGCCGACAAGTATCCAGAAAACTCGTGAAGCCTGAAAACTGTTG
ATCCTGGATGAGCTACACTGATAGCGAAGGACCTTCCCCACAAAACCAGCACTGCA
ACAGCTCCTCCACATGTTTGCGAAAGAACGTATAGAGGCACATCTCTCCATGGGAAA
TAGCCAGTCAATGAGAGAGCCAAGGTGACCTTTTTGGAAATTCTAGATAGCGGTGG
GTAGTATACAGGAGGCACC--------
ACGCACCAATGGACTGAGATGAGCGTCGCTGTATCGAGCAGTCCATACACCAACAA

TTATCCCGAGGAAATTCCCGCAAAGGATGGACAAATAACCCTAAGAGAGCTCAATA
ACTACAAGAAAGTTACCCTTCCGTCCAATGATCAGACTGACCCCTCCAGGACTTGTA
GGGAAACCTACGGATCAAATACAAACTAACAATAAGACAGCTGGTTGGTACTAGTG
AAAAACTCACCGCAGAGAAGAAATTGTGATGAAAGCGAAGAAGCGAGTCTGAATG
AATGATTAATGCATGTCAGATATGAAAGCCGCTCGTGAC-
GGGGGGGACATGGGTCGCGAACAGCATGCGTATCATCGTCCCCAGATACTCAGACC
CAGCGATCCGAATAGCATTTGCGTGATCGTCGATTACCTGGCGAACGCGGCTGCCGC
CTCTCGGAAAGCTGAAGGAAGCTGAAGCTCGGCTGTAACTGTGAACAGGCATTTTTT
CTATATCACTTTTTATGGTAGCAACGTCCATGGTCGAATGAAGGCCGGGGGCTGAGT
GGACTGCGGAAGTGGAGGTTGAGTTGGTATTTGGGAGAACGTTCGTGCTGGTTACAC
ACGCCACCTCTCAAACTTCAGTCAGGTATCTGCCAGCATGTAGCTTTTTATATCGCTT
GGGAGATCATTTAAAGAAGGACAGAAAGGGTTTGGTGAACACACGAGGGTTCTCTT
ACGGCGTATCACAGGTGGAATTTGCCCGACTTCCCTGCGTTTCAAAGCTTAAAGCTA
AGAACAAGGAAGCCTTCGG---------------------
AGTTCGAACCAGCGTGCGTTTGAGAGGCGGCCGCGGAGAAACTAGTACATAGCCGC
AGAATCAGCAGAAGGAAAGGATGATCATTTTGACTTCTCGGGCGTAGAATCTGATC
GCATACGCTTGCGCTTGCTTCATCGAGCCAGGGTAACCGTGATCAAGTAAATTGGCC
GACCCTGTGGAGATTGGCAATGGAAGGGAACGTCGGAGCATAAATCCTCGATAGAT
CCTGAGCTAGAGCGATGCAATTA-
TGATGTGAACCTCTCTGTGACATATTACTTGCAAATTGTCAGTTGGTAGACGCGACG
GATCGTTGTTGAATGCTGAACTGTGGGGATTTGAGAAGGAGAAGTGGGCAACTCGA
CCGATCGCAAAAAAGACAAGTGCCTCTTCTGGTTACCCGTGTGGCCCTGCAAGTGGC
TCCCCGTTACGTATGATATATTAAGTAATGTCATGCCCGAATTAGAAACATCCCGGA
TTATGTAGGAAGCTTCCTGTGGCTTGTTGAAGGGTCACACGAGCGGAGTATATTGGG
GTTGGGGTATCAAAGTGCCAAGTGCAAAGTATATATACGACTTCCACTACAACCTAG
CACCCACCATACTTCCAGTCCCAACCTCGAGGCCCCTTTCAAGCCAGAAATCTTCGC
TCTCCCCAACCAGGAACTCTCTAATCCCCCCTTCGAACTCTTTCCAAAACTCCTCAAT
GACACCCTCCGGGAAACAATTTGCGTCCCAGCCAAACGAAACGTTGATCTTACCCCG
TAGAGTAAATCCCATGATGAGCATGCCACCAGTGTTTTTACGAGTTTGAGAGACAAG
CTGGAGAAGATTGATAGCAGGGAAATTGGATGGGTCGTAAATCGAGTCGATAGAAG
GCCAGATGGAAATACCGATGAGAGCGGCGGAAGGGGGTTTGGGTGTTACCGATGGT
TTCGAAGCTGGTGCCAACGTGGATGAGGGCTTTAGAGGAGGAAGGGGAGGAAGTCC
AGCGACGGCCCTTCTCTTATCCTCCTCTGCTCTATCATCTACAACCGCCCAAGCTTTT
GCCCTTGCAGCTCGTTCCTGGCCCATGATCCGGTTTCGGCTGGCAAAGAACGGGCTC
GCCAATGCTTTCCGTGACTGTTCCCTGACGCTCCTCGCTCTGAGCCAGAAGATCGCT
TGAAGGGCCCTCTTTTTCTCTTCTTCGCTCATATCCTTGCCGGCAAAGCGGGGGAGA
AAGCCGGGAAGAATGACGTTGAGGTATCCCAATGTCAAGTACACGTATGATTCTGG
TGGTGGTGGATTTGCATCCATATAAGGACGTAGATTCACAGCAGTGTACACCATGAT
AGGTAACATCTCGTTACCAATTTGCTTCCAGAGTCGCTTCTCGGTAGCTGGTGTGAG
GGGACTGGAAATACGCGACGATACGAGGCGAATCCAAGCGAACGAACAGAGAACA
AAAATGGCATTTGACACCGTTACACCACGAGCTTTGCATTTAGCGACAATGATTTGG
GATAGGTGTTCTGGAACGGCCATTGTTGTAATCTTGTTGTGGCGCCGTCTGCTCGTG
GGTTTTATGCGGGGAAACAGATGACCTCCCTGGATGATACGTGAGTGAAATAGAAA
AGTAATGTAAACATGTTTGATTCAGAAGAATTCGAGAAAGAAACGCACTAGTTCCTT
GTTCTGGAAATTCTGGAACGCGATTTTCTCAGCTGCTAACCTAAATCTGTTTGCGGG
AGTATGCATTCGTTCCTCGGCTGGTTTAGGTATAGGCTTGAACCCTTCGGGCCTGAA

CCTCGCGCCTCTCTGTCCCCATCTCATATTCCACTCGTCCTCCAAGATACGCCTCAAC
TCCTCTTCTGACCGTGGTCGAGAATGATTGCTGGCGGAAGGGCCACCAAGAAGTGA
CAACAATTCATTCGTAAATTGATGTGCTGCCGTCCCATCGACCGTAAAGTGGGCGAA
ACCGATGAGCATTTCGCATGTTATTCGGTTTTTATGGCTGCTCTCAAAGCCGTGGAC
GGATCGGAGTGCATAAGCGTAGGGCGGAAGTGAAACGATAAGACAGCAGAGCCTCT
GATCCGAAAGAATTCTAGGTCCGTTGACAAATTCATCGGCCAGTTCTTTCCCCGTTTT
ATCAAACGAAATTTCCAGGGAAGCGCGTGCTTCACTAAGAGCTGCATTTGGGTTGGA
GGGTGGTATGTATCTGCGAAGGTTCAGTGAGCTTCCTGAGCGGTGTCTGGTGGGAAA
ATGGTATTACTCGAAACGAGCTTGATCATAACATCCAGGATCCATCCTGACGGAGGA
TGCAAAAGGGGGGTGTCGAATTCGCAGGATAGCCCATGCGAGATGAATTCGCCTGC
TTGTGACAATATCTAGTCCGCAATCAAACTTGAAGTAACCTGTAGAAATCAACTGAG
CGTCAGCGCATAAACAATTAGACAATAAGGGTGATGCCAACATATCATATCATTCGC
TCCGTCTGCACGACTCGGAAGATAATATGAGCTGAGAGAGAATTAAGCATGTGCTG
TTAAAGTCGGCAGACGCGGCCCCAAAAACAGAGTTGTCGACGCACAGCTCGTTGTG
ACCCATGATCCGGGCGTACTTGCCCGAATAGTTGGGCAGCGTTTCTTCGTCGTATAC
ATGCCTCAAATTAGTATTCGGCAAGGATGTGGAATCCTCGGTCGGGAGATGAGCCTG
AAGGAAGGACTTGGCCGTCATTGTTGGTGATGAACAACTGCAACGTCGCACCCATC
GGCTAGCTTTTAAAGGATGAACACGTACATGGTACTGATGATGGGGCGATGAAATA
AAGGCCGGTCAACTGCCGGACGAAGTTCTATCAGGAACGCGACAGCTCGGATGTGT
GAGGATCTCAATACATACGGGGACTTCACATTTACAGCGGATTACATAAATCCAGA
GTGAAATTCCAAAGAGAACGCGGAGTGAACAAAAACGTAGATCTGAGATGGCACTT
TTTGGGCCTTCTCAACTTGGGCCCAATTCAACTCCCCACCTGCTACCGTTATTCGAAG
ACATCTGCCGGTTTCAATTTTTATATAGCGAGATCCTTTTCCGTGAAAAGATCTTCTT
CGCCCAATCCCAGGTGGATGATTGTCCTGCCGCGTCGGATTTGCGATTGCCCGATGC
AACCTTCTACATAAACTCCACGCAAAGTCCCTTTTGATGTGATTTAGAACCCAATCG
ACAAATCAACTGTGAAACACGGATGAATGGCCGCAAGAAAGAACGGAGAGGATGT
CCTTATGTAACCCGTGTGGATCGCATGCACACGGGGCTGGTGGCCGGTCGTATTGGC
AAGCTCAGCGGATCAGTGAGAAGGGCAAGGCACAGTTTACATACAAATGTTGTAAG
TGCGAACCGATGGTCGTCTAGCAGGAACGGTTGTCCTAGTCCGGAACTGGGCTAGTA
ACCTGCCTAGTCCGGAACAAACATCATTGGGATTGCCCAAACGCGGCTGTGTCAGTA
CACGTGACATTCAATCACGTAAGGCCTTGGTAATGACTCAAATAGAGCGGCAAATT
ATCGCCTTCATTGAACCCCATGTAGTTCATTAGCCTGCGCCGATCAAATCTAACGCG
CCATATACCGCGGTATTCGCCAACAACCAGTTCCATGGCATCCGGGAATTGCCAATC
AATTCAATTGCATCAATCTTTCATTCAATTCAAGATGATCGCGCGAAGTACGACTGC
TCATCGACACGAGACTTGACAGCGGAGAAGGCACATATGTAACAGAACATCAGATG
AATGGACATGATAGACAAGGAACTGATAGGATAGGATAGGATAGGACCTCTTTACC
AACTGCAGATGCAACACGCCATCGAGAAGGTCTCGAGGTTGTCCGAGCGAATGCCA
GATATGTAGAGCGGTAAACACCGGACCAAACCTGACTGATATGCGGAGCAATCAAC
AATTGGGGCTCTCTAGCGTTACCTCTTCGAGTTTACTCCCGTGACGTGAATGTCATCC
CCGATTAGGAACTTCTCAAATTAGCTGTCACACGAGCGGAGTGTAATGCTTACAACC
TCGACCGAAACTTTCAAGAAAACCACCCATCGTGACAGCACTGAGCCCGGGGAGTA
ATCCCATCCACAGACGAACCAAATCAACCCGAAACCAGACCCCAGAGGACACCGTC
ACCCCGACCCTACTAACCCGACGACACTCTACAGTCCCCTCATGTACAGATGTGATA
TGCCGCTCTGTCACGTGCTAACGCGACCGCGGGGTAACAAATATGATATCAACAAC
AACAACCCGCTTACAACCTAGCATCTACTAAACTCCGAGTCCCAACCTCAAGACCCT
TTTCCAGCCAGAAATCTTCACCTTCCCCAACCAGAAACTCTCTGATACCCGACCCGA

ACTCTTTCCAAAACTCCTCAACGACACCCTCCAGGAAGCAATTTGCATCCCAGCCCA
ACGAAAGGTTGATCTTACCCCGGAGAGCAAATCCCATGAGGAGTATGCCACCAGTG
TTTTTACGAGTTTGAGGGACAAGCTGGAGAATATTGATAAAAGGGAAATTGGATGG
GTCGTAAATCGAATTAATAGAAGGCCATATGGAAATACCAACGAGAGCAGTGGAAG
GGGGTTTGGGTGTTGCCGATGGTGTCGATCCTGGTCCAAATGTAGGTGGGGGCTTTA
AGGGAGGAAGAGGAGAAAGCCCAGCGACAGCCCTTCTCTTATCCTCTCTTGATCTAT
CATCTACAACTGCCCAAGCCTTTGCCCTTACAGCTCGTTCCTGGCCCATGATTCGATT
TCGGCTGGCGAAGAATGGGCTCGCCAATGCTTTCCGCAGCTGTTCCCTCACTCTCCT
CGCTCTAAGCCAGAAAATCCCACGAAGAGCCTTCTTTTTATCCTCTTCGCTCATAGC
CCTTCCGGCGAAGCGGGGAAGGAAGCCAGGAAGAATGACGTTGAGATAACCCAATG
CCAAGAACACGTATGACTCCGGCGGAGGATTTTCGACCATATAAGGGCGCAAATTT
ACAGCAGTGTACATCATGATGGGTAACATCTCGTTACCAATCTGCTTCCAGATCCGT
TTCTCGGTGGCTGATGTGAGAGGATTAGAAATACGTGACGATACGATACGAATCCA
AGCGAATGAACAGAGAGTGAAAACGGCATTTGATACAGTCACGCCTTGAGCTTTGC
ATTTAGCGACAATTATTTGGGATAGGTGTTCTGGAATGGCCATTGTTGTAATCTTGTT
GTGGCGCCGTTTACTCGTGGGTTTCGTGCGGGGAAGCAGATGGCCTCCCTAAGTGAT
ACAATCGTCAATGTTATGACAAGCGTGTTTGATTCGGGAAAAGGAACGCACTATTTC
CTTGTTCTGGAAATTCTGAAACGCGATTTTCTCGGCTGCCAATCTTAATCTGTTTGTG
GGAGTATGCATTCGTTCTTCGGCTGGCTCGGGTATAGGCGTAAATCCTTCAGGCCCG
AACCTCGCGCCTCTCACTCCCCATCTTATATTCCACTCGTCTTCTAGAATACCCCTCA
ACTCGTCTTCTGACCTTGGAGAATGATTACTAGTGGAGGGGCCACCAAGAAGCGAC
AACAATTCGTTCGTGAATTGATGGACTGCCGTCGCATCGGCCGTAAAGTGGGCGAGT
CCGATGAGCATTTCGCATGTTATTCGGGTTTTATGGCTCTTCTCAAAGCCGTGGATGG
ATTGAAGCGCACGAGCGTTGGGGGGGAGCGATACGATAAGGCAGCCGAGCTTCTGG
TCCGAAAGAATTCTAGGTCCATTGATAAATTCATCCACCACTTCTTTCCCCGTTTTAT
CAAAACTGATTTCTAAGGAAGCGAGTGCTTCACTAAGAGCAGCATTTGGGCTGGAG
GGCGATATGTATCTGCGAAGGTTCGGTGAGCTTCCTGAACAATGTCTGGTGGGAAAA
TGGCATTACTCAAAACGGGTTTGATCGTAACATCCTGGATCCATCCTAATGGAGGAT
GCAAAAGGGGGGTGTCGAACCAGCAGGATAGTCCATGCTAGATGAATCCTTCTGCT
CGTGACGACTTCTAGCTGGCAGTCAAACTTGAGGTAGACTATGAACATCAACCCGGT
TAGGGTGAGCGTCAGTGCAGAAACAACTGGACGATAAGGATGTTGCCGGACACATC
ATATCATTCGCTCCGTCTGCACGACCTGGAAGGTAATATGAACTAAACGTGTAAATA
TGCGCCATCAGAGTCGGCGGACGCGGTCCCCCAAACAGAGTGTGAACGCACAGCTC
GTTGTAACCCATGATCCTCGTGTATTTCGTTCCCCCGTCATATTCGGGCCTCGAAGAG
GGGAGCCTAGTAGTCGGCAAGGCTGTGGAATTTTCGCTAGGGACATGAGCCTGAGG
GGAGGACTTGACCCTCATGGTTGGCACAACTCCAACGTCGCCCCCATTGGTTAGATT
TTAAACGATGATGGTAGCCTTGGTATCGATGATGGGCAATATTGACATGGGTGAGGC
TGGTCACCTGCCGGATGGAGGGATATCATGCAGGAGCCCGACAGATCTGACCCGGG
ATTTTACAGCAGTAGATGCAGAGTGAGATTCCAAAGAAAACCTATGGATCTAGAGT
GGCACTTTTGGGCATTCTCGACCTGGGCCCAATGCACGTAAGCCAACTCACCACCTG
TGTAGCTGTTTTCGAAGAAATCTGCCAGTTTCGATTTTTTTGCGAAATTCTATGTAGG
CGAAGTCCTCTACCGAAATCGGCCAACGATTCCGTGAATGATCTCGCTAACCGTTTT
AAATGATTGTCCTGCTACGCCTATCACATAATGAATAAAGGTGCCACTATGGCGACG
CAGAGATAGAAAGAAACATAAATCAAAGCAATGAAGGAAAAATAATATCAAATAC
ATACAATAAGATGATAAGAGTATCCGTCCTACATAATCAAACAACCTTCTCTCGTCA
CTCCAATTGACTCGCCCCGCTCGCAGCTTTCCAACGATATCAACGTCATCCAAAATC

CATCCAGCAGTGACTGGGGTATCCTTGGGATTGGGTAGTCAGCCAATGATGATTTTG
AGAATAATTCCGTCAAATCGGACGGGACTGACCACGATGGATTCTGCTTTGATAATC
CCTATCGATCGGTATGAATTGCGTGTGGTGGTGTGTCAGGGCTTGGGGAAGGGAAA
AAGAAATGGTGCCCATCAGTTACTAATCTAGGGGTGGGTATCTCCGCTACGTGGGAC
CCATTCCCTGTTCCTGCGGCTGCTCTCTTGACCTCTCGTAGGCCCTCATGGGCTCGCC
GATTCCCTCATGGTTCTCGTGCTTCCCCAATGCGCCGGGGTGCATCACAATTCAAGA
GCGTATACGTGCCATTCTAGCGGCCGCGTGGGCTTGTAACAAAGGCGCTAGGGAGC
GAGACCATCAAAGGGGGCATATGCAGACCGGGACACCGCACAAGGAACTACTAGCT
ACCTATTGCAGATTCTTCTACGTTCGAGTGAGTCTAAAGTATGCTCTAAAACTCTGC
AGAGTTTGCAATCTACTCGATCCAATGTTAGACGATACACGCTTCCAGTCAACGAGC
TTACTTCACTGGATCAATAGGGTCGTGCAAGTAACCATTGACATGTGCCTTCTGACC
CTCTGCGACCGCGTGGAAGGGATAGGGGGGCTTGGCCCTTGTGTGCCACCAATCCC
ACCATCTCGGTTGCCGTGCCCGCCACTCTCGCTCCTCAGGAACGCCCCTGCAGTGAG
AAAAAGAACTGCGTCAGTTTTCTTCCAACGCCCTCTTGCTAAGCTCGCCGCTATGCC
CACCGCTTCACCCGGTGCGTCGCAACAAGCGTGGCCGCCGTTGTTGTGGAGCACCCG
AATTACTGCCCGTTTCAGCCGCCGTACCTGGTGCACCGGTGGCGAACTATCATTACC
CATCATCCCCTTGCATCGGCACAAGAGCCTAGTGACCATTTAGTTTCAATAACTGGT
GGACGCCGCCTGACGCGGCAGCACCCGTCACTCTTCTCAACCCAAGGCGCTTCGCCC
ACGCACCACCGCACGCGCTACAACCATTTCCCGAGGCACACTGGCCTCTGAGTCCTC
AGGACTTGATCCATCATAGCTCCGATGCTAGGAGACTCCGGCTCGGCTCTTCATTAT
CCTTGCCTAACAGTGACGCTCCGTCATGTACGTCGTCCAGATCGTGCGAGATATCCG
GAATCCCTTCACATTCGGATGAAGCTCGTCCAACGTAACTAGCGCCTCTTGGTCTGA
ACTCTGTCAGGGACTCGACGCAATCTTGACTATTTCAAGTTCCTTGGTCTCTGTGACT
GTCGCATCTATGCACGCAACAGGCTGGGCTTCCGACAAGACAAACTCGATTGTCCAC
GTTGACTTCGAGTATACCTTGCGCTATGAACTTCTTCGTCTCTGTACAATCTCGAATC
CCATGTCCGTATACGTTGCACATATAACACGTCAGGCCCTGGCGCTTAAGTAGCAAC
TCTGCGACCTGAACCCGACTCATCTCAGGTGGCGTCGTCAAATCCGTCCACAATCTT
TGCAAGCAGAGTTCACCTTCTCCGCCTCCACAAACTGACGAAATGCGTAAAGCATG
GCAGAACCATTTCTCATATTCGAAGGGGCATGCAGAAGTATGAAGACATTGTCATA
GTCAGGAAATCGGGAGGCAAGACGGACGCACGTGGGGAAGGCATGTGGATTTCGTA
ACAACCACCAAACAAAGTAAAACGGTCACGAATATGCGACACAGCGCAAACAGTTC
AGGAATTGCCATGATTTTCAGCATTGGAAGTCAATTGGTGAGGGGACGAAGAGTCG
GTTACACACCTGTGTCCTCGGTCAAGGAACTCAAAGCTAGCGGGTGGGCCAGAAAC
CATTGTGGGACTAGCGATGAGTAACGATAGGATGAGAGTTAGGTGAAAATATTGCT
TAGTGACTGGTTATGCAGAATTGGCAATAATAGAAGACCTTGTCATCAATCGGCTAC
GAAGTTAGCGGGAATTTTTGTTTCCTTGTTGTGTCCGCTATCCGCTGTTCGTTCAATG
CCACCAACAGCCTCTTTCTCACCCGATGAGTAATGTCATCATGCCATGATCCGCGGA
GCAAGATAATACGGGACTTGTTACTGCATCGCGCAAAGGAATTAATATAAGTAATA
CCTGAACCCGAATGATACAAATTGGTGTATTCAACATGAAGAAGAGGTCAGCGAGA
TGATACAAGCAGAAAATAAAAAAATCCTCAAGGCGTGAAAAAAATTATGGTTAGAT
CTTTTGAGCCCCTTCGTCGGTTCCTTTCTCAACATCGTGGTCCGTCGAAGGTGGAGGG
GGAGGGGGAGGAGGTGTGCTCGATTTGAGCCATGTATAATAAACCGTGCCTGCCGT
GATGACGAATATAGAACTAGCTCGATTCCTGGGCAATAGTGTGGTCAGATCAATTTA
CACAAAGGGAGCTTCGACCTAAAGACTTACGGTGTTATCACATCACCAAAAATGAC
AACGCCCAACACGCTTTGGATGACGCTCCTTGCGGCGCTCGAGAACATGTGAGTGA
CAGGGCTGGTCACTTTGATGCTCAACAACCCCGCCATACACAACAAGAACCCGAAA

AAGCCGGTGACAGCACACCCAATNNNNNNNNNNNNNNNNNNNNNNNNNNNNNNNN
NNNNNNNNNNNNNNNNNNNNNNNNNNNNNNNNNNNNNNNNNNNNNNNNNNNNNNN
NGCTATCAAGTTTGTCCAATATGCCATTCCAACGACATTGTTTCCACCCATATACTGC
TGTGCGGATTTGACCATGACGGCATGCAACGCAGTCATGAGTGAGGAGAGGATGCC
ATAAACGAGTGAGCGGGCCGTGACATCGAGTGCAGTTGTGTGACGGGAAAAGTATG
AGCTTGGTGTTGCTCCGATGAAGAAGCCACCAGTGACGACAAAGGCAGCCCGTAGG
ACTTTATATCGGGGCATGGTACGGGTCGCGATGGCCGAGACGACGACGGTGAGGGG
GAGGATAAGGCCACGAGCGATCTAGAGCGCGTTTGGACATCAGTCATATTTCGTAA
CGCGATGTGGCATCAAAAAGTTCATACTTGGAAGAAAGAGGCGTCGACATCTTTCA
GGCAAAGAGTGTTGAATATTATTCCGGCGATACCAATAAGCGAGACTGGAGCGAGA
GTGTAGAAACCTCGTAGGTCAATCTCAGGGAGCTCAGCGCGAAAGGGGAGGTAGGA
GGAAAAACCGGGGGTTTGCGTTATCCATTTACAAACGTGCAACAGTGCGATGCAAA
TGAAAGTTTGCACGAGCTGAGTGAAAGGCAGGTGTCATGAGGGGCCATGATCAACG
ATGATGGCTTGAACTGGGACATACGAGGAGAGAAAATGGTAAGTTCGGGGTGGAGT
TCAGAACTACTTTGTTCACGAACACCATCTACAAGTGAGAAATCAGTTTACAGTCAT
CGTGTGAACCACAGTTTTAGCAATGATAATGTAACTGAATCAATGGGGACTCTCAAC
TTACCACCATGGCCGCAATCATATAGAACGCCACAACGGTGGCTACCTTGAGCTTCG
ACGGTTGAGCCTCTGCGTCAGACATGATTGTAGTAACTGTTCCTATGTGCGGGATTT
CAAGGGGGGGGCACGTAGGATTCCAGGGGTTGTCCAAAAAGCTTAGGTTGACCTAG
GACCTTGCTCTTCACGATTGTCTACAATGAAAGGACAGGAGTCGTGAAGGCAGCGG
AAGAGAGAGAAAGTCAAGGAAGCCTGGTACGTAACATTATTCAAATTCACCAAGTC
TGGAACTTTGGTGGTCACGTGGGAACAAAGAGTGGATTCAAGCGTGAATCTTAATTG
TCTGTTGTGACAGTCCCGACTATCGCGGGCACGTAGGTTAACAGCAATGGCTGGTAG
ATACAACAAAGATCGGAAGTGTATGTCTGCTTGTCTTGGAACGGTACAATCGCGCGT
TTTCTTGGTGGATCGACTTCGCCTTTAGATCTGTGAGAATACATGTGCTGCCCCACGC
AAATACCACGCAATATGAGATCCCGTTATCACACCCTGTCTAATGTTGAATTCGCGA
TAAGAAGGCGTCGAGAAAGCACGGGAGCTGTTTTTACCTAAGTGCTGTTATCGACCC
CATATATATACTTCCCTGCATTCATTCCCTTTTCCCTTCGTCAGGGCTTCATCCGTAGT
ATATCCAGGCCATCGAGCACTCTTCATGTCATGTCGTGGAGAAGCTGATGTTCCTAT
CCCTGAATAATTGAAAACGTCATACCAGATTGGTCTGACACTAGTTGTCAATGTGTT
CAAGTGCATTACTGCACAGATCGCGAGTGAGTCTGCTGGTGGCAAGGGATGGGAAT
GTGCGGAAGGAAGAAAGTGAGAGAACAATTTGTTCGCCGGAACGGACGTTGGATGT
TCCATGCAGTTCTTCTACCTCCAGAACCACCGATGTGAAGGTTGCAGCATCAGCCCC
GCAAGCAAGCAACGGTCCAAATGAATGCACGTCCAGGCACGCGAGCAAACATGAG
CGGCTGTGATGATTCTGATCACCCGTCCTTTGGAGGTTGCATCATTTATGAGCGGCTT
GCTGCTCTTCTCCCACGCACTCTTGAGCTTGGAGCCCCCTAATATGAACGACGTTCG
ATTTTCCACAGATGAAGTACGACCCCAAGGATAAACGATGATGTGAATTGCGTCCTC
TTGCCTCGCTCGTGAAGTGACCAAGCACAGATGTGAGTAACCCACTAGTATTGAACC
CGTGTGAAGATGGCACGAGGGAAAACTTGTGCGATTCGTGGAAAATTAAAAGCTCG
AGGTGGCTTGGCCTAGTTGTGCGGGTAAGCATTAGTTGATGCCACGATGGCAAGGC
GAAGGCTGCTGGTACCTGTAGGGGCAGCTAGGTGGATTGATGGGGACCAGGGGACT
TATGTACATAATGAGTGGGCAAACTAAGAAGCGTCCGTTTGTTGCATGACATTGCAT
GTGGCACCACGGAGCAGGCACGTTCATTGTAACATTCAGTCAACCTGTGGATTAATG
TGTCAAGAACAGCGCACATACGATCGGAGGCTTCAAGTAAGATCCGAAGCGACGCC
GCATGCCTGCAAGCAGAATGCAGATGTGTCGCAGAAATCAGGCCTCCAGAAGCTAT
GGCGCTTCGTTGCTGGATTCTTCTCAGAAGGCTGGAGAGGTGGGTCATCAGTTGGCT

ACCCCTGTCACCAATGACCAGACGAGGGATTGCCTTGTAGGTTGCGTATGAATCAAG
TCACGGCTTCACTCCCAACTTTGTTGTTCACAGACAGTGAAAATTCCTCCTGGTTTCA
GAAGCAGCGGACGCTCCTCCGGACAGGCCGCCTTGAGCTTCGCAAGGCTTCTATTTG
GATTTATGTAGCTCATTCGTATATATACCCCCGCATACGCTTCCCTTTTGGGTGATGC
AAAAGCTCAGGTTCGAGCTCAGTCCCTGCGGCTGTTGGACAAAGCCCTGTAAAACG
GCCCTCTCCAAGCGGAGGAGCAAATGATTGCAGCATTTGTAATTCGGTTGCTATGGT
TTGATCATGAAATCACGACTGTGGATATCCATGCTAGCTTCCCCTGGATGCTGTGTC
GAACGAGGTTTGTCGGCATGCAGGGGCTGAGACTGTCGGGTTTCCGGACAATGACA
ATCAGAATGTCGTTGTCTAATGGAGAATATTCGGACTGTTTCCCAGACATGCTGTGG
GAGCCTCAGCGGTTTCGTTAATGTCTGTAGCCAAACGTCCGAGTACATCGTCACCCG
CGCCCGAGCTGGAGGTACTAATCAAGGGCAAGAACAACGTGCGAGAAAACTATTAT
TGGGGCGTGTGCGTGAGTTTTCGGAGGCAAATATCCATCTATATCTGGGCATGCCGT
CTGCATATTTCATTTTGTAAATTTAAAATGCGATTGTCTCGAACATGTTCGCTTCCTC
ATATGTAACTCATTGTCCTTACGTCTTTATGCCATTTCTTGTTTGGGATTCTTCCCCTC
TCCTCTTATTCGTGGTTGCACACAAGCTTTTAGCTTCTTGACCTTGGACATTTCCTGC
CAACTCCTGTAGTTTTGCTACTGGTGGTATTTCTCCTTCGCTGGACTGCCTGGCTGCT
CTCTTGAAATCTATTCTCCCACTCCTCAGGCCGCTTTCACAGCCCCAGGATGTCGTCC
GGACGCCCGCCGCTTCGCGCTTCTCCCGCTCGACATGTGGCTCTGGACTCTACGTCC
CATTCTTGGGCTGACAATCATCCCACTACTGACGAATATGAAGTGCAAACCCCAAAT
TTCAAGTTTGAGCGTATTATGAGTTCAAACGAATTGTAAGTTCATATAGTTATTTTAA
CCTGAAGTATCTTATTCATTGCGTAGGTCGTACTACCTCCCAAGTCGCGCGGATGGC
GCCAATGACACGTTCGGTGCGTCCAATTTTTGTTCTTCCCCATCACTCACTTTTCCCA
ATACTCATACCCATGCGATTTGCCATATGTAGTGTACTTCAGATTCGATTGTGCTTCC
GGTTTTATCACGCCCCAGAGGATAAACCTTGCGTGGGCTATCCTTCGCCTCCGGCAT
CCCCTTTGTTCCTGCAAAGTCCTGATGGACGCAGGCGAGTATGACCGTGTTCGGTTT
CTGTACGTGATAATCATCCCCCCCCCCCCCCTCCCCGCTCGATCTAAACCTTGTTTTAAT
TCGCCTCTGCACAGGTATACAGCACCCTCCAGTCCCACCGAGGCACTTGTAGAGGCG
CGCCAGCACTATTGGATCTTCGCGAGCAAGACCCGCGAGCAATTAATGGATATGTGT
AACAATGGACCTCGTATTCTTTCGAACGAGCGACCTTGCTTCCTTTTCGTTACTATCC
CCCCTCAGTCCTCTGACAGCGAGAAGACGGTGCACGATATTTTGCTCTGCTATAGCC
ATTATAGCACTGACGGTCTAGCGGGGCATTACCTCGGAAACGAGCTTCTCTCGCTCC
TCGGTGGCAATTCCTCAATTTCGCCGTCCTCCCCACGCTCTGAGGCAGAATTATGGC
ACATGCTTGAATATGAGTGGGAAAGGAGGTGGGGAAAGAAGCAGGGTTCCATTTAC
AGCCCGAAGGGATTTAAACCAGTGCCTAGCGCAGCCGAAGAGAGAATGGGACGTGA
TGACCGCAGGTTAAAGAAAGTTGCAGAGAGAGTGGCGTTTATGAACAGTTTGGATA
AAGATGCGGTGAGTGTGTTGTCGGCCTGCTGCTTTTTGTAACGAGTGCAGACCTGAT
CCGCGTTCTTCTTTTGACCTTGCCCTGGATCCAGGGCGGTCATGTGTTCCCACGAATC
AGGCCTACCTCTTCCGCTCGCAATTCGAAGATCCAAACGTTCATCATCTCGCAGCAG
CACACCCGCTCCATTCTTTCCTTGTGCAAATCTCACCATGCTACGGTGACGAATGCT
ATCTTTGTCCTGGTGACGTTTGCATGGCTTCGCCTAGTTGCATCTCGTACTGACCCTC
GCAACGCGCGTGTCAGCGAGGCGGAGCGGAAACGATGGAAGGCGATTGGGAGCGA
GCGGATGCCCATCATGATGTACACCNNNNNNNNNNNNNNNNNNNNNNNNNNNNNNNNNNN
NNNNNNNNNNNNNNNNNNNNNNNNNNNNNNNNNNNNNNNNNNNNNNNNNNCCTTCCTGGA
TTCCTTCCCCGCCTTGCTCTCGAACAATGTAAATCAGACGAAGAAGCGAGGAAAGTG
CTAGAGAGAACCTTCTGGTTACGTGTGCGGAGTGTACGAGATCAACATACGAAGGT
ATTGAGCAGTCCATTGTTCCCGAGTCGGAACCGGGAAATGGGAAAGGAAAGGGCTG

AAAGGGCCAAAGCCTGGGCGGTGATCGATGATTACGAGGAAGAGGTGAGGAGAAG
GAAGATGAGAGGGTTACCACCCCCGAATCCTGGGCCCAAGCTTACTACAGCTAAGG
CGGAAGGTGGCAGTCGAGTTGCGCCGAAGCAGCCAAGGGTTCCATCCGTCGCTCTC
CTTGGCGTATCCCTCCTCGCTAACCTTGACAACCTCTATCTACCGTCCAACTTCCCCG
CATTGAACCTTTTTGACATCCTTAACGGTACCAGGAAAAATACGGGCGGGATACTGC
TCATGGGGTACACGTTTAGAGGCAGGATTTACTCCACTCTTGGGTGGGACTCTCGAT
CGTTCGAGCCTGGCGTTGTGGAGGGATTCTTCGAGGAGTTCNNNNNNNNNNNNNNNNNNN
NNNNNNNNNNNNNNNNNNNNNNNNNNNNNNNNNNNNNNNNNNNNNNNNNNNNNNNNNNNN
NNNNNNNNNNNNNNNNNNNNNNNNNNNNNNNNNNNNNNNNNNNNNNNNNNNNNNNNNNNN
NNNNNNNNNNNNNNNNNNNNNNNNNNNNNNNNNNNNNNNNNNNNNNNNNNNNNNNNNNNN
NNNNNNNNNNNNNNNNNNNNNNNNNNNNNNNNNNNNNNNNNNNNNNNNNNNNNNNNNNNN
NNNNNNNNNNNNNNNNNNNNNNNNNNNNNNNNNNNNNNNNNNNNNNNNNNNNNNNNCGCC
ATTCATACCCCCCATTCTCCACAATGCTCGTCCTATCCCTCCTAACTGCATTAATATT
TTTCTTCCAATTTTTTCCTTGGAGATGAATTCCTACGAAAGTTGACATCCTTTGTTCA
GAGTTATTAGTAGTCCCCCAAATGGGTCGAGTTGCTCGATACAAACAACATTAAATA
CATACTCAATGCCGCTCTTGTGAGCGGTCCAAGTCGAAGTCATAGACTGAGTCCTGC
CTAGTCTAAGTCTGGGCTAGATTGATAGTAGTAACAAGCTCACTAGCCCGCCTTTTA
GTAAACAAGACCGCTCCTCAGGCTTTGTGAGGTTCCTCAGGTCACTGGGGTTGGGGG
GTTGCTCCGTTCGACCTCTCCTTGCCTGAGATACTTTTTCCAGTCCTTCTCTCCGATGT
TAAGAACGTATCTACATTGGTGCGAGGAACAAGGCATCAAGATAGACCCTCGATTG
TCGGTTCGAGTCCCTTCAGATGGCCAATCATTGAGTACAAGTAATGCGCCTGGCACA
GACGAGACTCAGCGGGGCGCAAGAAGCGTACTTGCTGCGGAAACGGGAAATGTCCC
TGACATGGACGAGACTGAGCAGGGTGCACATGGCCTTTGCGTGTTTGCCACTGCCTT
CATAGAAAATAGAGAAATTGGCAAGCTTTCGGATGACTTCACATAGATCCAATGGT
CCTAATTTCTTTCTCCCCCTCTCTTTCTCTCTCTCTCAGTGGCGAGTATTCCCAAATCC
GCTGTCTTGTCTGCTAGAAACTTTCGATATTCCAGAGGACTGGAAGAGTTCTTATGT
GAAAGGGAAGTCGATCCCAACTTGTCCCTGAGCCTTGCACTATGCATTGAGCGGTGT
GTGTGCTGCTGTCTTGGGAGCTTCTCTCTGTATCTTCAAACTAAGTTTTTCTCTCTATG
TTCTCGATCAGACAATTGGGACAGGCTTCTAAGTGGTGGGGGTACATACAAAGTCTC
CCCAAAGATCCAGTTGATCTCGTTGCCTTCTGGCCTTCCGATGCCAGTGATGACGAT
ACGAAAGAAGCTCTGCAGTGGCTTCGAGGGACTGGGTTACTTCGATACATGTCCTGT
GAAGCTCAGCCATCACTCGTAAGTCTCCTGCGTCTTGTTAAGCAGCTCAATGTGAGA
GATTTTTACTGGCATAGTCAAACATTCTATCTTTCTTCAATACGGTTTTCCTGCCATTT
TCTGGCAGTATAGGCGTTCAGCCCGTCACTTTTGACGATTTCTGCTATGCCTTTTCGA
TGGTTTCGTCGCGAGCATTTCGTGTGGATAACTATCATACCTTGGCTCTTGTTCCTAT
AGCGGATGCGTGCGTTGGACCTCTCAATGTTGACCCAGAAACCAAGCGTCATGTAA
AACTTTGTATTTTCAGGTTCAACCATGCGGAGGAGAATACGGTTCATTTAGAGGTAG
TACACATGATTAATTCGTTCCCCGCACCACTTCTGATTGTCGATTTAATCTAGACTGA
CTACTTTGTATGCGCAATTTGTGGCTCCTTGGATGAATGTCCGCATGACCGGGACGA
ACCTAAGGCTCGTGCAACCACCGGTTCTACTGTATCGACTTCGGAAGAGGATTGCTG
CGAAATGGTTACAAATGCCGTGCTATACGGCGGAGAAGAGGTGTTTAACATCTACG
ACCAGCGACTCTCGAACGTCAAACTCATGTCACATTATGGATTTCTACTCGATGGGA
ACTCGTATGACGAGATTTTGTTCACAGTGCAAGAGATCGAAGATTACCTCGCCCACA
CTCCTTCCCAACGCACACGCTTGTCCATGCAGCTCCTGAATGAACTGTGGCAAGACC
TGTTTGAGAGTCAAAAGCTCTGGGTGGAATGCCTTCCCACATCATCTCCCTTCTTTTC
GCCTTTCATCGAGGAGACGGAAGACGACATAGATAGGGAGTCTTTGGGGAAGCGCC

TTGATAATGAACGATTATCAGCTACCTTCTTCGCTATAAATGCGAGTGCTCAAATAT
CTCCAGCACTATGGCTTTACCTCCTCTTACGAAGAGCCCCGCAAGCCATGCTTGACT
CTATTGCCCCACCCGTCGTGCAGGCGCGGAACGAATCGCTTCTAGGGTATCTACGTG
ATTTGGCAAAAAGAATGTCTTTGTTGGATGTCGAAGATGAGGAGTGCGAAATTCATG
GTTTACGTCTGGGCAGTTGTCAAGCTCTCATCGACATCGCAGTGGATATTCTGACCA
TCTGCCGAGCGAGACTTGCCTCCATGCATTGGAGCGACCGACCCATATCAGAAATCG
CCGAGCTTGAAGTGCGTCTAATTATACTCCCACACAACATGCACTCGCTTACAACTT
TGGATCAAGGGTGCACTTCAACGATCGAGATATCGGCGACGGACAGAGATCGCAGT
ACGATTTGCTCTCAACGAAAGATTGATCTTGGACAGCTGCATCAAAATGTGGGAGA
ATATCGAGTCACAGTTGGCTAATTTTGGCGCAGACCATAGTGTCTAGACGTCCAACA
ATTTTCACATACACACTTAAGTTAGAATCTAAGCTAATTCTCCACGAAAATAATATT
GCAGCAGCCCGAAGAGAGCAGTGTGATTCTGTCAAACGTCATCAATGCATGGGTGA
CGGCAATCGCGACCCTTCCCATCCGCCTCGAAAGGCTGCAACGGCGGAAATGAAAG
AGTAAAAGTTCGTCCAAAACTGATCACGTCCGAAATCAGCGTACGAGACAAAAAGG
AAACTGACACGTAGTTTCAACGCACCTCATTCTTATAGAATCCTCTGACCAGGGACC
TATGCATTTACAAAATGTCCGATGATTATTCAGGTTTTTCGGAGGGTGACACCAAAC
TGACCAGTGCAATATGACAGGCAGGCGAGAAGCATTTGCTAACAATTGGTACCTAA
CCTGCTTCTTTCTCTTCAGCA
>C._cibarius_Al-2_scaffold1560_len=9010

------------------------------GGAATGCTCCATGATT---------GAATCCGT-
GTATAACAAGCGAGAAAGAACGTACGAATCAAATCCTCGTCGTTATGAATCAGAAT
TTCATTTGGTGGAGCACCCGCCTTCTCTGACTCTTTGAATATGAGGTCCGAGTCGAA
GCCGTCAAGGAGCTCCAATAGCGGTTCACGGAGTGGGTCCTCGACCAGCTTGTATTT
T----ATCG-----
AGGATCCAGAGCTTGTGCGGGGCAGCGCAAAGAAGGCCCGTCGCTGGGCAGTCGAC
AAGTTGAGCGGCTTCAGCAAATCATCGAAATGCGAGCCTTCCTGTCCGGCAGGTATC
CCCGAGGTGTACCATCGATCAATGAACGCCCGGACATGCTTCAGAATCTCATGACCT
TCCTGTCTCGTTTGGGCATCGTCATCGATTAAATCGTCGATAACTCGGCAAAAGGCA
TACCTAGGNNNNNNNNNNNNNNNNNNNNNNNNNNNNNN-------------------
NNNNNNNNNNNNNNNNNNNNNNNNNNNNNNNNNNNNNNNNNNNNNNNNNNNNNNNN
NNNNNNNNNNNNNNNNNNNNNNNNNNNNNNNNNNNNNNNNNNNNNNNNNNNNNNNN
NNNNNNNNNNNNNNNNNNNNNNNNNNNNNNNNNNNNNNNNNNNNNNNNNNNNNNNN
NNNNNNNNNNNNNNNNNNNNNNNNNNNNNNNNNNNNNNNNNNNNNNNNNNNNNNNN
NNNNNNNNNNNNNNNNNNNNNNNNNNNNNNNNNNNNNNNNNNNNNNNNNNNNNNNN
NNNNNNATGAGGAAATTCGTAATTAAAAAGAATACTGCTTCTCTGAATAAACACAT
CACGGGAGCGGGACGTTTCAGAAAACAGAGTGGCGAGAAAGGAGGCGAAGACGAC
GCACTCTATGTCGAGATGGCCCCAAAGCTGAACATCAAATTTGGTGCCAGACTCGAT
AACCCAAGTACCCCGTTGAAGAGCTGTCGCATCAAGAATCCACATATACGCAGTCG
AGAGGAGAATAGGTCCAAGCGAGGATAAGGTGGGCATGGCGATGATATGAAGTCCT
GCTACGCTCCTTCGAAACGG---TGGGTTCACA-
CATTCAACCAAAAAACAAAGAAGCGAGGA-
TTATTCACCAGATAAACTGAAGGATGGGACACGCCCAAACGAGTATGAGACCCATA
TACATTCCTTGGCCTCCTCTCCAGGATAACCATGCACCGCTACCTGTTCCAGCAGCA
AACCATAATGAGCCAAGGCGATGGAGCACCGTATGCGATGTGGATGGGGGGTGTTC
ATGGGTGGGTATCAGGGCAGGATGTAACACGGGTTTCGAAAGGATGAGGTACAGAA

CTGAAGTGAAGCATGTTTGGATGGCAAAAAAAAAGAGTTCTTCAGCAGGAATGGAA
TATAATGTCACGTTAAACGTCGCGTCTGGCGGGTATGACCAAACCTTTTGTG-
AAGTTGTAGATTTTATCAACAAGAGAACGCAGTGTCAGATCAAAAGTCGAGAACGT
ACGTTGCTTCGGATAAGATACGAATCCCATGGGATGGTGTACGCGGTAGCGATAAA
GACAAGGATGCCAACTCTGAAGCAGTCGATAGAAGTCCTCAATGGGAAGTAAAGTG
CCAAGAGCACAGAAAACGCAGGTATAGTCCAGCAGAGATGACTATT-
AAGCATCCAAATTAGAT-----GGGCTCAA-----
TGTTAACGTGAAGCATGTCACCTACACCAAATCGTAATCCGGGATTCTCGTTAGAGA
CACCGCGTCCAAGATGCGTTCCAT-AGCAGGTAGAAT---
AATGGGGGGATGCACGTTGCACGTCTGAATGATAGGAAGGGA-GCTTCA-ACTC-
GAC-CGCGCTTGCCAAGTGGCTCCGTGGCACCTGTGTTGGTAGAATGTATTGGAA-----
-----CTAACGAACAATG------TACCAATGACAG----
GGCGAAGATGGGAGGAAAGCTAACATTGGTGTTATAAAAATTTTGACATGCCATCTT
CATCACTCGCAGGGGTTCTGAATTGTTGATGGGTGTTGACATCCGGGTCGCGGTTTC
CGACAGC--ACATGAAAAT-----------TA------TCGGGTTCCATT------CCG--AACCAAT------
CATGCCTGTCAACGC-----GTTCTTCTAAGAATGGAATCCGAATTTGATGA--
ACGGATGCCATG-TAAAGG-------
TCACATGATTGATCTGATTTAGGAAAATGAAGGTCGATCATCATTTATTCGC-----
TACCATTTTTGGACAAT-CACA-TGATC-CG----CCCGGACATTCATCC--------
AAATCGGGTGCGCCAATTTTTTTTTG------GGCTTGCTGTAA--------TGACTTGACCG-
GGTCGACTCCGAATGAATGACCCTGCTGAATGA------CAGTC--TCAAAATGGGCAGA-
-GACACCG-GAAAAATGATTTGA-AGATTCCCTTCGACATTC----
ATTGACTACGAACCAGTGCCTCTTGATAAGTTAAGGTGCAGCCAACTATGTAACGGC
CCAAAGGAACGTTTCC-TTCTCTAGGGA--GAAATC--------GCT--
CCATCAATTCCCAGCCCAGGCTGTG-CCTCATA-TATTATCAACCGCACGTT-
TCGCACCTGGTGGGACAA-CAGTTCATCAGGCTGACGCACCCTGTGCTTG--
AAGTATACCCAGAGCTCTCATTACATTGCCGCCGTTCCCAAAAAATCGACCACCTTG
ATCTTATCGGGAAGGTGGCGAAATCATGGCGCTTTCTGTTGATACATA--
AGTAGTATAGTA---------GCGGCATGAGGACGTGACCCCAT--GTCCCGAGACTT-
CTGAA--CTCAGCACGACTAG---CACTGGGGTACAGAATAACAGTGCCAATC-TG--
ATAG-AGAAAACA-CATAACAGGACA-TCAGGCACCT---TGATCACCA-------
ACTGCAAGACCGAAG-TAATTACAACACA-----GCATCGA---GCAATCAAGAGG---CG--
--TGC-AGCGGACACAGATGGGGAAC--AAATACATGTTATTGGAGCCTGGT-
GCCTAGGTGCTAGGCTGGAGCTGGCTGGCATTAGCG-AATCA-
ACAGTACACGAAGGATTGTAAGCGGCAGGCGACT---GGCCAGAATG--
AAGGGGGCATGGAATAGAATTCTTCAC------ACC------TCGATT-----AAATCTT--
AGTTTA-----------ATATAATATCCTATT----TT------ACGTGTCGGT-TCCGC--GCCGA-
GGGAGCTATGATTGATGATACGTTCCTGAGTCAGTCCGCACACCGTGAATCAGCTCT
TCCGTGGATGAGAC---AAAACTTTCTCA-
TCACTAAACCCCAAAGATATCAACGTGAACAATTCCATTCACCAGCACGACAGGAA
GGATGGGGCATCTGAAGAATAAAAGAAGGCACCAGACAGAAGCTCGGGACGAACT
TGGTATGAGAAACAAAGGAACCACATCGGTGAGCCCTTTCTCTGCTTATATGCGCTC
AAGGATACATCCCGGCCGACCT-TGGGATCGTGAGTCGAGACTTT--CTGCGAAGAGT-
CTTCAG--ATTCAGA---TTTACTGGGGT-
ACTCATCGCCTTTGTGAGGGGCGGCAAGCAGTTGTCGAACGATACAGGCACGCACT
CCTAAGACGTGAGC---------------------CATGATATCGATATCTTTTGCACTTG--

GCTCAAGATGTCTCCCCATAGTGACTGTGTGGGGCGGTTGGATTGCTACGGCGAATG
C-----AGG------GGGTTTCACGG--TACC-ATTGA-GTCTG--
CCGTCAAATGCCCCAAACAAGGAAACCTC-CTT---
CCGTGAGCCAGAGTTCTCACCAACTAGGCGGTCGACCTGGTTGCCGGCCGAGTTTTT
TGAGAATCGCATGTGCCAGTGACA--
TCTCGAGAATATCAAGGCCTGATCTCAATAGAATTAAAAACCGTCC--------------------
--A-------------
------------------C-----------------
ATCCGAAACCGGGGAGGGACAGAGAAGCTAGTAACGGCAGCAGGAACTGGACACG
GTTCGAGATAACACATCCGAAACCTTTTGAATAGGTTGCGGATTGACCATCATCAAT
GATTTATTGTCGAGCAGCATTCTCATAATGGTAGCATGACGAGTACTGGCGGACCAC
GCGGCCCCCCATACAGCAGCATCAAATCCCCACTCCACGAACAGCCTGAGCAATAC
TTCCGGGGAGGTGGCTCACTAGAAATTGGTCGGCATCGATATCAAAAGCTACGCCTG
CAGAGGTTGGGTTCCAACGCAGGGAACAGAATGTCAGTCAGAAAGACATGACTGTA
TGACTCGGGCGCGCCACGTTGGCCAGGCCATAGGGGTGCCTGATGGTCTGCCGGCC
CTTCTGCCACCTGTGGCCCGGGCGTCGGGTTGCCACTCCGACACCAGATAACATAGT
GATGACGCACGAGATGTCTGCCATGCGGATGATGTGATTCCCTCACGATTGGTTGAG
AATGCCCTCGCAAGCGAAGAGGAAGACATATCTATCGCTTGAAACTCCTAACACGC
TATCCAATGTAACACCTCACACGTTCCATGTCTTGCATGTTTCGGACTAGAAGACTG
GCCATTGAAGTTCGTCACAAGCAGGAACTTGATATGTAAGATCGGCGACCTGAATCC
ACGGGAAGTCGCTATATAGCCGTGAAAGCTCAGGCTCAAGGTGTCAGAATGGTGTC
CCACAAAAAGACATGCACGGCGCAACAAACGGAAGAGTAATCCGCAGCCCGTGAG
TGGTATCCCCTTTCCAGGCGAAATCGGCTTCTACTGGCCACGAAAGCAGAAAGGGC
CTAAGCACCCTCGTCTGTAATATACAGAGCATGCTGTTGTAGACGTACTAGCGCAGA
AGTTGACGATGTCCACGAAAACAACCTCCCTGTTCCTTATATGCATAATTAGACAGA
AATCAATCTGGGGGTTATAGAGATAAGGAGAGTCAGATATGTACGAGGTCGAGACC
TGCATCAGTCTGGTTCCATGTAATTAATCGCTTGCCACAGTCCGAGAAGATAACCGA
ATCGGGCTCCGATTGGGATAT----CAATAGGATCTGATTTTTTGCG-
AGGAAAATACGTCATTGATCGATCCAGAAGGGCACTACT----
ACTGTAGCTAGAACTCAACCCGTGTTGCCGCGGATCAGGAACAAAGGGAATCAATG
GGACCGTTTGGCGTGACAGCAGATATAGGCCGTGCCTCGTCGTGGGAGCTTATATAG
GGCCTCAAAGTCGCCAGAAAACTCGGTAGTCAGGGAAATGTGTACGTGCCCCACGG
AAGTTGGGGGGACAAGTTATTGAGGATGTATCTTAGACCGAGGAGCACAGATACCA
GTGTCATCCCCATGGATATGAGAACGCCGCCCTTGGATCAGGGATCAGTTTCCGACG
TCGGAATTCCGTATTAAGGGACTACAGTAGGATACAGTAATATGGATCAGACCCACT
CCTTAGTATGGCTGGTGTATTGGTATCAAAGTATAGAACCATCGTCAACGTACAGCA
CGTCGCACAGTAATCCCGCTAATATGGGGCCGCAAAGAGGACGGAGCATGTTCCAT
AGTATTGTATACCAGGAAACGTTGTCAGCCGGGGTCGTGTATATCCTAGCCCCTGAG
AAGAGGTGGGGCACGAGGAAATTGGCCTGCGGAAAGCGTTGGGATCAGACAGAAA
GTGCGCCCACAAAGGAAGCTACAGTCCTACTGACCCAGGTCACCCACTCGCTCAGA
ATTGTCCAGAGCGTGAGTTCTATGATGTGCATACAAAGCACGATGGGTACTAAAGTG
GGGTTGCGTGTCCCTTGTTTATCATTGACCGCACGAAGCACGGCCAAAAAGATAAGC
CCAGAACAGACAACTTCGAAAATGAAGCATGCAACTTGAAGATACGCAGTCAGCTT
TGCAGGATGAACCGTGACGATGCGGGCGGAGGAATAGAAAACGCTCACTGGCCGAC
AAGTATCCAGAAAACTCGTGAAGCCTGAAAACTGTTGATCCTGGATGAGCTACACT
GATAGCGAAGGACCTCCCCCACAAAACCAGCACTGCAACAGCTCCTCCACATGTTT

GCGAAAGAACGTATAGAGGCACATCTCTCCATGGGAAATAGCCAGCCAATGAGAGA
GCCAAGGTGACCTTTTTGGAAATTCTAGATAGCGGTGGGTAGTATACATGAGGCACC
CACNNNNNNNNNNNNNNNNNNNNNNNNNNNNNNNNNNNNNNNNNNNNNNNNNNN
NNNNNNNNNNNNNNNNNNNNNNNNNNNNNNNNNNNNNNNNNNNNNNNNNNNNNN
NNNNNNNNNNNNNNNNNNNNNNNNNNNNNNNNNNNNNNNNNNNNNNNNNNNNNN
NNNNNNNNNNNNNNNNNNNNNNNNNNNNNNNNNNNNNNNNNNNNNNNNNNNNNN
NNNNNNNNNNNNNNNNNNNNNNNNNNNNNNNNNNNNNAAATTGTGATGAAAGCG
AAGAAGCGAGTCTGAATGAATGATTAATGCATGTCAGATATGAAAGCCGCTCGTGA
CGGGGGGGGACATGGGTCGCGAACAGCATGCGTATCATCGTCCCCAGATACTCAGA
CCCAGCGATCCGAATAGCATTTGCGTGATCGTCGATTACCTGGCGAACGCGGCTGCC
GCCTCTCGGAAAGCTGAAGGAAGCTGAAGCTCGGCTGTAACTGTAGACAGGCATTT
TTTCTATATCACTTTTTATGGTAGCAACGTCCATGGTCGAATGAAGACCCGGGGCTG
AGTGGACTGCGGAAGTGGAGGTTGAGTTGGTATTTGGGAGAACGTTCGTGCTGGTTA
CACACGCCACCTCTCTAACTTCAGTCAGGTATCTGCCAGCATGTAGCTTTTTATATCG
CTTGGGAGATCATTTAAAGAAGGACAGAAAGGGTTTGGTGAACATGCGAGGGTTCT
CTTACGGCGTATCACAGGTGGAATTTGCCCGACTTCCCTGCGTTTCAAAGCCTAAAG
CTAAGAACAAGGAAGCCTTCGGGGCATAAATGAGTGTGTTAGTAGTTCGAACCAGC
GTGCGTTTGAGAGGCGGCCGCGGAGAAACTAGTACATAGCCGCCGAATCAGCAGAA
GGAAAGGATGATCATTTTGACTTCTCGGGCGTAGAATCTGATCGCATACGCTTGCGC
TTGCTTCATCGAGCCAGGGTAACCGTGATCAAGTAAATTGGCCGACCCTGTAGAGAT
TAGCAATGGAAGGGAACGTCGGAGCATAAATCCTCGATAGAACCTGAGCCAGAGCA
ATGCAATTGTTGATGTGAACCTCTCTGTGACATATTACNN------------
NNNNNNNNNNNNNNNNNNNNNNNNNNNNNNNNNNNNNNNNNNNNNNNNNNNNNN
NNNNNNNNNNNNNNNNNNNNNNNNNNNNNNNNNNNNNNNNNNNNNNNNNNNNNN
NNNNNNNNNNNGCAAGTGGCTCCCCATTACGTATGATATATTAAGTAATGTCATGC
CCGAATTAGAAACATCCCGGATTATGTAGGAAGCTTCCTGTGGCTTGTTGAAGGGTC
ACACGAGCGGAGTATATTGGGGTTGGGGTATCAAAGTGCCAAGTGCAAAGTATATA
TACGACTTCCACTACAACCTAGCACCCACCATACTTCCAGTCCCAACCTCGAGGCCC
CTTTCAAGCCAGAAATCTTCGCTCTCCCCAACCAGGAACTCTCTAATCCCCCCTTCG
AACTCTTTCCAAAACTCCTCAATGACACCCTCCGGGAAACAATTTGCGTCCCAGCCA
AACGAAACGTTGATCTTACCCCGTAGAGTAAATCCCATGATGAGCATGCCACCAGTG
TTTTTACGAGTTTGAGAGACAAGCTGGAGAAGATTGATAGCAGGGAAATTGGATGG
GTCGTAAATCGAGTCGATAGAAGGCCAGATGGAAATACCGATGAGAGCGGCGGAA
GGGGGTTTGGGTGTTACCGATGGTTTCGAAGCTGGTGCCAACGTGGATGAGGGCTTT
AGAGGAGGAAGGGGAGGAAGTCCAGCGACGGCCCTTCTCTTATCCTCCTCTGCTCTA
TCATCTACAACCGCCCAAGCTTTTGCCCTTGCAGCTCGTTCCTGGCCCATGATCCGGT
TTCGGCTGGCAAAGAACGGGCTCGCCAATGCTTTCCGTGACTGTTCCCTGACGCTCC
TCGCTCTGAGCCAGAAGATCGCTTGAAGGGCCCTCTTTTTCTCTTCTTCGCTCATATC
CTTGCCGGCAAAGCGGGGGAGAAAGCCGGGAAGAATGACGTTGAGGTATCCCAATG
TCAAGTACACGTATGATTCTGGTGGTGGTGGATTTGCATCCATATAAGGACGTAGAT
TCACAGCAGTGTACACCATGATAGGTAACATCTCGTTACCAATTTGCTTCCAGAGTC
GCTTCTCGGTAGCTGGTGTGAGGGGACTGGAAATACGCGACGATACGAGGCGAATC
CAAGCGAACGAACAGAGAACAAAAATGGCATTTGACACCGTTACACCACGAGCTTT
GCATTTAGCGACAATGATTTGGGATAGGTGTTCTGGAACGGCCATTGTTGTAATCTT
GTTGCGGCGCCGTCTGCTCGTGGGTTTTATGCGGGGAAACAGATGACCT------
NNNNNNNNNNNNNNNNNNNNNNNNNNNNNNNNNNNNNNNNNNNNNNNNNNNNN

NNNNNNNNNNNNNNNNNNNNNNNNNNNNNNNNNNNNNNNNNNNNNNNNNNNNNNNNNNNN
NNNNNNNNNNNNNNNNNNNNNNNNNNNNNNNNNNNNNNNNNNNNNNNNNNNNNNNNNNNN
NNNNNNNNNNNNNNNNNNNNNNNNNNNNNNNNNNNNNNNNNNNNNNNNNNNNNNNNNNNN
NNNNNNNNNNNNNNNNNNNNNNNNNNNNNNNNNNNNNNNNNNNNNNNNNNNNNNNNNNNN
NNNNNNNNNNNNNNNNNNNNNNNNNNNNNNNNNNNNNNNNNNNNNNNNNNNNNNNNNNNN
NNNNNNNNNNNNNNNNNCCCATCGACCGTAAAGTGGGCGAAACCGATGAGCATTT
CGCATGTTATTCGGTTTTTATGGCTGCTCTCAAAGCCGTGGACGGATCGGAGTGCAT
AAGCGTAGGGCGGAAGTGAAACGATAAGACAGCAGAGCCTCTGATCCGAAAGAATT
CTAGGTCCGTTGACAAATTCATCGGCCAGTTCTTTCCCCGTTTTATCAAACGAAATTT
CCAGGGAAGCGCGTGCTTCACTAAGAGCTGCATTTGGGTTGGAGGGTGGTATGTATC
TGCGAAGGTTCAGTGAGCTTCCTGAGCGGTGTCTGGTGGGAAAATGGTATTACTCGA
AACGAGCTTGATCATAACATCCAGGATCC--

>C._appalachiensis_Al-2_scaffold1419_len=12185bp
TTCCCTCCCACCACTCCCACCGCTCCCACCACTGAAGGAGCGGAGCAGGATCACACT
ATGAAACGGTTTACCTGCTTCCACAATTATGAGTTGATACGATGCTGAATTACCTAC
TATTGCTAGTGTTTTGGTTGCACTTGTATATATCTATCTGCTGTTTATGGGACAACAA
ACCAACCTGGATGATCTCAACTGTATGTTAGGATCAATTACACTACTGTTTTATATTG
ACTTTACTATAATTTCGAGTCAATATTCACCAATTTACCACTTTTAAATAGGATGTGA
TTTTCCCTTAATTTACTGAAGAAAATATTATGAAAATAAATCCTCAATAAATAACAC
TGATCACAGTAGTGTCAAGACTGCTCTTTCTGTCGCTTAGAGGGCGTGCTAGTTATC
GAACGATTAATAGACTTTCACTTTTCACCTGTGGTGTGATAACCAATAGTTTTAAGA
GAAATTTCAATTAAGAAGTCCTCTCCTCCTTATTCTCACTGTGCTGCCCTTTATCAGA
GGCAGCTAACTGACCCTAATTCACGCTTAGAGTGATGCACTAGTTATCTAGCGATTT
ATTATCACTTTTATCTTTTGGAGTGATAGAACCAACTCTGGTGAGGAGAATTTATCTT
TATTCTACCTAGGGGTTTGTGAAGGGGCAGAAGCGGGGTTCTTTTATACCTTTCAAG
AGCCTCCATACTCCCACACTTCACGCTATTTACATATGATACATTTGTATAGAGAAA
GTACTGGAATATTATAAGTTCCGGGTTTTTGCATATGAAAATGGCTCCGGATAATAA
TCATAAGATAGATGACCAAGTTTTCCATTGCTGCGCTGGGTCTTGGCACTGCCTTAT
GGACCTTAGGCTGTACTTTTCATGCGCCTTTTACCAAGCAACTTTATATTTAGAAATG
ACTCAGTCAGAATAGATCCTTCTAGACCCTCCTGCAATTCACCCAAAATTAAAGTAA
TTTATTAAGAAATTTCCGGCATTTGTTCCAGCCGTTCTGAAGATCTGAGATGATCCAT
CCCATGTCCTTCTCCATCGCCTCGCTTCGGATCATCAACCATTATCATGCTACTGTTT
GTATTCGGCTTAAATTAAAGGTTGTTTAATGAAGTGGGAATTTCTATGTGACCTTCTA
GACCTGATTTAATTTCCCCTCTCTGAGTCATATACTGGGCGTCCGTACGCGTCGCTGG
ATACCCTAGCATGTGATCTTTAATTTCCTAGGCCAATATACTTTTCCCAAGTCATGTT
ACCCTTTTTAAGATTTATATATCTTATTTAAGCCAAAACTTTAGTCGTCGTACCGATC
TGGTCATATCTCCGTCAATTCTCAACCGTTTTTCATGAAATCAAAGCTTCAATAAACT
TAAGAAACAAAGCTTTCTAACGCAAAAAACTTCATATTTTTACATATTTTACCCGTT
GAGAAATGGACGTTTCTTTTCTCCCGCTTCCTTTTGTCATTATTCCGGTACTCATGTTC
TTGAGTCGTAAATGCTTCAAATATTATTTCTCCTACACCGCTCCCACCGCTCCCACCG
CTCCCACTGCTCCTGCCACCTCACATACTCCCCACCACTCCCAGCTGCTCCTGAATCA
TGTCCCCCTCATTTCCCCCCTCATTCTACTCTTCATTTCCCCCTTCATGTTCCCCCCTT
TTCCCCCCCCCCTTTCCCCCTCCATTATCCCCCTCCATTATCCCCACATTTCCCCCCCA
CCTCCCCCCTAGCTTGAACATCCCAAACCCCAGAAATGTCTAAGTCAACATATAGAC
AGTATATCATGCCCAGACTTGAACCCAGCCATCTTTGCTCCGTGGATGCACACACAA
TGCAATTTCTGTTTGTATTGATGGTCACAGTCACGGTTGCATAATACACGAGTAGTC

ATGGTTTGTATTTAATTGTTGGAGTCACATGTGCCGGCAATAATGTTAAATCCGATC
ACCTACTCCGAAAACCAACCATGCAACACTTTGCTACGATGACTGGTTCACTTTGGC
AGTGGCGATGTCCAACGGCAACTTCCGAAATCAATAGTAGGATCATGCATGTTGTAA
ACAGGTTGTAAAATATATGTTTTGAATTCTAACCCATGTGGTCTTGTACCCAACCTA
ACCCAGAATGAGTTTGGAGGAGCATATGCAATTGAGAAAAAGGTTTTCCTGAGTGA
GAGTCGAACCTGTGCAGCCAAAAGAAACTGCATAGCCTCAACCAACACTCTAGCAC
CTATGCCACCAGGTACCAGTCCTACTATGTCATCAAAGTACTATATAAAGGGTTAAA
ACTCCGTGGGGGCTGGCATTAACTTCACAGCATTAATTGTGCTCCCCAGGTCATTCT
AGGTTGGGTTGGGTACAAGACCGCATGGGGTTAGAATTCAAAACATATATTTTACAA
CCTGTTTACAACATCTGTGATCCTACTATTGATTTCGGAAGTTGCCGTTGGACATCGC
CACTACCAAAGTGAACCAGTCATCGTAGCAAAGTGTTGCATGGTTGGTTTTCGGAGT
AGGTGATCAGATTTAACATTATTGCCAGCACATGTGACTCCAACAATTCAATACAAA
CCATGACTACTCGTGTATTATGCAACCATGACTGTGACCGTTGTCCAAGGGAAATAG
AGGGTTTCCAGGGATGAAAAGAAGGGGGCAGGGGATAGGTCGCTTTAGGGTAGGG
GTGCACAGGTTCGATACATGATGTAGGGAATCTACAGTGCTCAATGTATCAGTTCTG
GTGGTCAATCTAACTCACTGGTCTATAAGACACTGGAGTTGTCCAATAATCAAGTTA
GGAAGGGAGGGGGGAAGCGGTTCAGTACTCAGTAGTCTATACTCTTCACAAGGGGA
GGAATCCAAATTGCATTTCCTAATGGAGTTGTCCTTTTATATGTACAGGGAACTACC
ATATAAGGAATCGGAAATAGAAGGAAACAATAGAGAGGAACATATAAATGAAAAC
AAATAGAAATATAACATGAAAATAATGGCCTGCAAAGATCAGGGCCGCCAGCGCCA
ACCGCTGGGCCGTTCGCCACCCACGTTTGCAGGTGGGTAGCCCTGCTTCCCCTCTTTC
CATCGAGCCCCTGATCGTCGTAAACGGATCCATACTTCCTGCGAACGGGCGTGATTA
ATCCGGAGGGTTAGTGGGAGCAGAGATGGAGGAAGTGAGGGCCGGAAGCGGCCTG
GAAAAATATGTAAAATATGGATAGTATAGGGGATGTGTTTTAGAGGGTAGGGAGAG
GCCTCGATGGCCGTGGGGAGGCGGGCTTGCACAGGTACATCGGCTAGGGTGTAAAA
TACGCGTTTGCTGGGTTTCTTCGGTACACGGGCTTTGCCCTGGTACACCGTCAATACA
AACAGAAATTGCATTGTGCATGCGTCCACAGAGCGAAGACGGCTGGGTTCGAGTCT
GTATCATGCTGCAAATTTGAGCCATATACAGCCAAGGCCACATGCTTCTAGTGTATG
CTATGAAATTTAATGCAGAAGTTAAGCATAACATTGCTAGGTTAGTCCTAGGGTAGG
AGGCATGTAGGGGCTTTCTTTTTTCCTCCCTGGAGCATCCCTGTCCTCCCAGGAGTAG
CATAATGCTGGGAGTAGCATCATGCTCCTGGGTACTCTGGGAGTAATCTGCAAGATT
TGCTGTGCTCCGGAGACCAGGGCCCTTCAAGGGGGTGCTGGAAATTCCCGGCTGAA
ACATATGCCAGGGGGGGGGAGGGGTCTGTGGGGGCTTGAACACGTAACTTCATGTAC
TTATGTGCAAGGTACAGTACATGGAATTTGAGTATCCCCTCCCTATTATTCCCCAACC
TAACCCATTTACAGCTCAGAATTACTCCTACCCAGCTATAATACCCAACCCAACCCG
TTCAATACAATAACTGGTGCTGGTGAGGCCTCTCACCTTCTTGCTTTTGCGATTGCTA
CTCCTCCTTACGGTAGGTAGCCACTTGAACTCGCCTAAAGCTCCCAC----
CTGCTGCCTTGGTTCCACTT-GGTCACCCTTCCGCTGTGTAAGC-----TGACACTGC----
CTTAGCCCACTAT-CATGTTTGCTTGCCACCACCACCACCTTAAAATTCATGCCTT-
TTCATCCTACTTACAATTTTGGTTATTC--
TTTGCCTCTTGCACATGCGATATAAGTAGTTACAATGGGTTGCACACCA----
CCAAATAACCACTCGGCTGTTTCCCTTGAAACAACCTAGTGTCTTCAATTAGCAAGA
GTT---CTGAATCTCAGATCAACCTGCAATGCACACATCGTTC--
ACTTTCAGCACAAACAAATACTTAGATTCCCAATTTTGGTTCAAACTCAATT----
GAGCCTAGCTTGAAATGGGTTAGGCTGAG-TAATAACT-
AGCTTATAGTGTGGGTTAGGTTGGGGAATAATAGGGAGGGGATGCATCTCAGGATT

G----CCAAATATGGACAAGGCAACTGCCAGGCCTGC-
AGTATTCTCCGATTGAATTAGGGAGGG--
GGGGTGGTTTCCCGAGATCAGGGAAAACACATAACTGGCATATGTTTCTGCTGTGCA
TTACCGGCACCCCCTTGGGGCCCTTTCCTGGAGTGCCTGATCTCCTC----------
CTGGTTCTCCAAGTGCTCCCCATTCTCCAGAGCACTCAGGAGCATAACA---------------
A---------------AGTTTTGCTGATGTCTAC--------
CAAGATCACTTTCACCAAAATCAGAACTGGCAATTTTTTTGCAAATGGCTGTAAGTT
CC--GAG---
TTTCCTGATGAACAATGTGAGACTCAACATCACCAAATGTGAGTTGGCCTCA--
GCAT-
ACTTCATGATTCCGAAAGGAAGTGGGCCCGATGGTTGATATTTCCAACAAAATAAAC
TTCTGA----ACAATATCATACACGTCTGCTAGGCAAGATGTCTGTGTCCT------
TCAATTGTCTCGCTTGCTCAGAAGA---ATCG--GA---ACATAATACTGAA-
CAGTGGATCGACCCCCCGTGACTTTGCTTGGCTCAATGCTTTCAGAGATTGCGAATA
GCCGAAGAGTTGGTAGTGCTGGTGCAATATTGACGTCCTTGAACTGATAAGCAAAAT
TAGTCTGTACAGTTGAAGATGT-------TGAATTCCAT-------TT---CTCAA----
ATTGTACACTTGTAAAAAG-----
AAACCTTGGTATGAACTATCTTACAACGCATTGCGATATCAACGACAGCATGCACTG
AACTGGAAACAAGGCATCGCTGGTGAATCAGTCGTCGTCTCCGTCGTTGTCGTCCCA
AATTGCAATCTGGTCCTGCGACACCCATCGAAGGCCTCCCAGAGCTGTCGCGTCTGA
AGCCTTGGTTTCAAGAGCTAGAAGAACAAGTAAGTA-
CCACATGAGGCCTTGTAGTG-----GAGTTGAGACTCACGAATGAACTA---
AAAGGAACGAGGGTGGACAGAGAGCGACGACGTCAGACTTCATGACCTACCTGGGT
ACTAAGCGAAGAGAACACGTACAACAGCGGTGAAATCTTTCTTTTCATTTTGAGGAT
AAAATAGGATTCGGTCATTGTTATAATTGACAACTGATTTCGTGCCTTTCTTGATCTT
CTCGATGTCGTTGGTAGACAAATTTCCCCAGACAGCTGTAGGGGCATACTATCAACC
ATGTTCCGTTCTATGTTATTA---
GAAAATACTCACAGACATTGCCACTGATGTCGTATCCAGCAGGGATGTTGAACGTG
ACCTGTTTGCGGACAGGAGCGAGGGTGATATTGACTTCGCTTTCGCCAGATCTCTTT
TCAACAAACGCGGTGGCTGTTGCCAGTTTGAGGGGATGCCACGCCCCCCCTTTGTGT
TGAGTGCCATCTACGATCCACCAGACGGTGCGCGCCTGCG---
TTTCACTTACTCAGGCGTATATCTGGAACACATGGAGACAGCCACCTACCGGATCTC
TGCCTACAAACTGAAAAGTAGAAAAAGTCGTCAGTATTATAAGCTAAATGAGAGCC
C---------AACGTACCTCGGCGTAGAA------
CTGGCCCTGAGTGCTGCCGAAGAACTGAACGATGACGCAAGCGTCCTCGTTCTTCAC
AGTGTTATCACTATATATCACGTTATACTTCGTCTTTCCGCGGATATCATCAATGTTG
TTGTAGAACAAGGTGCCGACGGGATCTAGAACACAATGCACACCATTAGCTCTCAG
TATCAAGCAAGCCAACACGCC---
ACTACCTCTTCCATAGGCAACATTGTCGACGAACTCCTTCATGATCTCGGGTGCCTC
GATGCGCACACCCTGCTCTTCCACGCCAGTGGCCTCGCCGAAGTAGAACAGAGCAG
AGGCGCCAGCCGACCAGTTGTATTTGTTCAGGCGTTCTGCGTAGGCACCCTTGTATC
CCTGTGAGATGGCGGCAGGAGTGTAACCCATTGAGCTTTGCTGTCTTGGTGTCGGTG
TGTGAGTTGCTGTACAGGTGTGGTTTGAGGCTGGGGACTTCGAGTCGAACCCATGAG
CT-TTTATACGTCGATTG---------------GCATCAGGAAAG-CCACAAGGAACGG---G-------
----CTCGGTATTACCGGTGCTTAC--------------CCTG--GTTTTCGA-T-----------
TCTTTCACTACT-------GACTGCCATG----------------------C-----------CCAGGCCTGCTG-

TCATAATTCATGGAA----TC---------AATAGCGCCA-
TGTGCACTTTCTTTCCTCGTTTGCGTCCACTGCAG---------------------GCAT------CTCAC-
---------------T-------------TACGGAGTGACAATCCGTGGATGTTA-----------------------GT----
--------AATAATGTTAGTCATGATT--------------GGCCA--------------
CTCTTATCTGTGTTGTGA---------GCG---T---
-------------ATTGATCCTGCAATGCAT----------------ACATGCTTCGG----------------------------
----------------------------------AAA-------------GCTCTCCCCGTTAAAGAG---
CTGATCACCTGCCTATTCA--------GGGT-------------------------ATT-----CACGTCATG-------
------TCATTTAGCGGAGT-TGTCCGAAATGATTGTTACGCAAAATCACGTTCTGTG------
--TT--
---C---------------------------------
-------------------------------GTAGT----AAACCGTTGAA----------C------------------------
TCTCAT-------------ACTTACCTTCTCC---CATC------------------------------CCGGA-------------
--T--------------------CCCATATCCGCC--
CCCTCCCCACCTCTCCTCTCTGATTCA----------CTTGT----------------ATAACA--------------
---------------AGAA----------------------TTTGTTTTCCCCAT---
GTGTTTGTGCGCTCGTCTGGGGGGTTGA-----------------A--------------------
AGCCCTACCCT----ATCTAGAAAAGCGGTTATTC-----GACACTGTG--------G----------
GACGTCCGC-CTTGAG---GCTTTTATGACGCCAGCTGC--CTGTAAAGACCC-----------
ATACTGTTGC--------A--------GTCCCCAGGCTCTGTCG-------C---------
TTCCGCTCATGCTCACACTGCCCTTGGATCTGACTAATCTA-----------A----------
CGGAGTCCCTG---------CT----GCGTT--------AA----------T--------AGTTAGTACCAGCCCA-
----------------------GC----------------------------CGGC------CTTCGTTTTCATAT--------CC--------
GCTGTGGGC--GCAATTAA-------AAAGAA------GA--------TAGATATGCTACTAC---------
CCATGCATGCCGTGCGTG-------------------------AA-------------------CAGCTG-----A-----------
---------------------CTGCAGCA---------GCGGTG---------CT-----------A-------
GGACCCTTGATGT---
CCATTCTGTGAATCTGT--GC----------------------------
G----------------------------AGCGGTGCCC---------ACGC-----------
TCCGAAGCAAGAGCCATGA-----
AATGTTTGTACCCCCGAATACACATTTTTTCCAGCTGCTTC-----------------
CTGATTACTA----ACGA-------------------------GTA-----------AC-----
GATACTCACGGGACTTTCATGAGAAGTCGATAGTGTT----------------------------------
AGGTGGAAC------A----------TCTCAGGCG-----TGACAAATGTATCTCTAGTGT--CACTT--
-----TACCATTG---A-----CGTTC-------------C------CTCATG------
GTCCCAATACCTCAATTCTGTTAGG------CGGTCAGCCCCTAGACTG------------
ACTACG------------AGTCC--ACTTCA-------
TAGTTCTGTTAC----A---------------CAA-----AC----GATGCCCCC----------A------------
GCGCGCTA---TTCCGT-----------------GAAT---
--AGC-----------C----------AAAGTACGTGA-
----------G------------TCTGTGCATACT----T----------ATGT----TTCCATC-------CTGCC---------
--A-------------------------CAAGTGGCGATCTA-TTCAC--------AAGTGGCGATCTATT--------
---C--------------------ACAAGAGGTGTTCAAATGGTGG-
-------CAAGTCTACATCA--TC------------
ATCCAAGAAAATA--T---------------------------TCATCAAAGAGA------
AAGATCATCTATAATTGTCAAAGCCCCTTCCAGCACATCAACCCGTAACGAGGGTAG

ACCAGGCAACCCAGACCCTTCTCCACTTCGGTACACTAGGCCTCGTCAAACGGGTCT
GGAGAGGAGTCATTGCGAAAATGTCATCCGCGTCCAACGTGTATCTTGGATCACCGA
TCCTCTTCTCCAACTGCCTACCGATTTCCATGTAGCTCTCGACCGCGACCGTCAAACC
TTTGCGTGCTGAGAAAATGGTTGGCATGTCACCGATAGCTGGTCGCGAGGCAACGA
AAAAGTCTTTCGCCATTCGAAGCAAACGAAGTCGATGAATTCGAGCTTCGTCAGGTG
GAAAATGGTACGGCGATTGTACAGCCGTGGTGAGTTTCGTTTTCGATTCGTGATGAT
GGTCA---
ACAGAACCTTCAGCCTTGGAAAGATCACCGATCCACCATTCTTGCGGAACGTACACT
CTCCCGGCTCGTTCATCCACCCGAATATCGCGGGCGATATTTACGAATTGAAGGGCC
ATACCCATCTTAACAGCTGCGTCCAAAATCTCCTCACGAGGACGAGGGGAATCGTA
GGAAGTTGGCGCGTGGTGGAAAATCATGTGGCAACACATTTTGGCGACCGTACCAG
CAACCCGGTATGCGTAAACTAAGAGGCCCGAGGTGGGCCATGATTAAATAAACAGA
ACTCGTTGCGTGACAAGCGAGGAAGAACGTACGAATCAGATCTTCATCGTTGTGGAT
CAGGATTTCATTTGGTGGAGCACCCGCCCTCTCCGACTCCTTGAACACGAGATCCGA
GTCGAAGCCGTCAAGGAGCTCCAATAGCGGCTCACGGAGGGGATCCTCCTTCAGCC
TATGTTTCGGGCTTCGGGATGATGATCCAGCGCTTGTGCGCGGCAGGGCGAAGAAG
GCCCGTCGTTGGGCGGTCGACAAGTTGAGTGGCTTCAGCACATCGTCGAAATGCGA
GCCTTCCTGTCCTGCAGGCAGCTCTGAGGTGTACCATCGATCAACGAACGCACGGAC
ATGCTTCAGAGTATCATGGCCTTGCTGCCTTGTGCGGGCGTCATCGTCGATCAAATC
GTCGATAACTCGGCAAAAGGCATACCTAGGTGTCGGGACTTGATCAGTCAGAATGA
CTAAGAACAACCAAAAATCTTTTACAAGTTGATCAATTCTATCCTCAAGGGACCCTG
GAATATGCTGCTTGCAGTCCAGAAACTTCTACTTTTCCTTTTGAGGATCAAACTGGTG
TCCCGGAGGTTGTTGAGCCGGTGTGTGACCACCTGGTCATTCAAGTACCTCGAGGTC
ACTGAAGATGAGGCCACGAGCGAGAATGGAGCCGAAAATAAAGTGTGAGACGTCG
TGGAAGGAGAATCTATGGCGTATATGGTAAGGATTCCGTACGCCCTGTCAAAGGCA
ATTTGACCAAAGACGATGAGGACATTTGTGACTAAAAAGAACACTGCTTCTCTGAAT
GAATGGATCATGGGAAGGGGACATTTCAGAGAGCAGAGTAGTGGCAGATGAGATG
AAAACGACGCACTCGATGTCGAGATGATCCCAGAGCTGAACATTAAATTTAGTGTCT
GACTCGATAACCCAAGTGCCTCGTTGAAGAGCTGTCGCGTCGAGAATCCAGAGAAA
CGCAGTCGAGATAAGAATAGGTCCAAATGAAGACAAGGTGGGCACGGCGATGATAT
GGAGTCCTGCAACGCCCCTTCAAAACGCAATTGGGCTCAGAGCATTCA-
CCCGAATACGAAGAAGGGAAGAGTTACTCACCAGAGAAACTGAAGGATAGGACAA
GCCCAAACGAGTATGAGACCCATATACGTTCCTTTCCCCCCCTTCCAGAATAACCAT
GCACCGCTACCTGTTCCAGCAGCAAACCATAACGAGCCAAGGCGATAGAGCGCCGT
ATGCCACGCAGACGGGGGATATTCACGGGTGGGTATCAAGGCAGGATGCAAAACAG
GCTTCGAAAGGATGAGGTACAGAACCGAGGTGAGGCAAGTTTGGATGACGAAAAA
AAAAAGTTCTTCGGCAGGAATAGAATATAACGTTATGTTGAACGTTACATCTGGGGG
ATATGACCAAATCTGTTGTAAAAAAAAAAAAAGGTTATTATAAAG-GAACACAG-ATC-
GA-
AGAAAGTCGAGAACGTACACCACTTCGGATAAGATATGAATCCCATGGGATGGTGT
ACACTACGGCGATGAAGACAAGGAAACCAACTCTGACGAAGTCGATAGAAGTCCTC
AGCGGGAAGTACAGTGCCGACAACACGAAGAACGCCGGTATGGTCCAGCGAAGAT
GGCTATGAAAGCAGCCAAATTAGATTGGGGGGGGTCAACGACGAGTTGACGTAGGA
CATGTCACGTACACCAAATCGTAATCTGGGATCTTCGCTAGGGA------
GACCAGGATGGCTTCCATAAGCGGTTGGATTCGCTACAGAACGATACA-
GATGCACGTATGCACGCTGCAGTGGTACGCTGAATAATCAGAGGCGCGTCCGCCAG

GTAGCCTCGTGGCACCTGTTCCGGTAAGATGCGTCGGAAACACAGGGCACTAACAA
AGAAACGAAGGCAACCAACAACAGCAGAGTGAAAGACGGGAGGAAA---------
GGTGGTGTATAAATCTTGACATGCCAT------------CAAGCTTGCTAAATTGTTGAT--
ATGT----AT--GTATC-
TGGCTTCCGACGGCGTACAGGTATATCAGTGCCGAAGTAGAGAACTGAGGCGCCTTT
ACGTGGCCGGGCGCCTATATCGCCCATAGCTGGCAATACTTAAAGCACAGCAATCA
AAGGAGCTAGAATATATAGACAACAG-
TGTTGTGGTATCGGCAGTACTTCGCATCAATG--CTGCGTT-----AATTAAGAATAA--
ATCATTTAACCGCGGTGATTACACAACCCGACACTACACACTCGTCGCGGGATCGCA
CACACCCAACCACGCCAGAACACCCAACCCTGCAATTGCTGCTAGAGGCGTTGCTC
GCTGCAACTACCACGTGAC--GACAGAGATCGACGCCACATCGA--
ACGTTTCCAAATGATCTATGCAGGCGTTCACA---
CACAGAATGACCCTGCGAGTGCGGGTTTGAGTTACTCCATCCGCAAGTCTGAGATCG
AGAATGACC------------ACAACTTGTAGCGTAGCCTATTCCGG-
TCTTCCTCGAGACAGGATCCCAGGCTCCAGCCATCGAATACAGTACTAGGCTAGCAA
TGCCTTCCAAGCTCGGCCAATGACCTGGTAGTCTCTTGAGTCTCAGGTTATCTCATGT
ACGAATATAAGCAGTTGACCAG--
TGAGGAAGCCTCGGCCGGCCAACACGGCCCGGGGCCGTTTCGAACTTG--
TCCATTCCCCATAATTC------CTTGCTAGCTTCG---ATGCTTCGTAATCTTG-----
TTCTCATCTCACATCTCCGTCCTTCTTTT----CACTCGCAG--
TCCCCACGCGCGCCGATAGGTTC---
GATTTCCAGAAAGCCCATCATTGTTCGGATCAATTGGCCAGGTATTAAAAGTG--
GATCGTGGCATCGCCGATGATAGCTGAACAAGCGAGTCGGCGATCTGCGTAATAAC
CTTTTACACAGTGATAAAGCAAGGCTCATTGCAAAATAATTGAGCCGCGATATACAA
CCAGTAGCTATCGGATCTCCTAGCCAATTCTAGTGGAAAAGGTAACTAGGAGTGA-
TGGCACATGAAAGACTACG-GCCAAGCATCACAT--
TTGGTTCTAGCCTAAACATAGAGCATAGAATGGACGGTATGAACAACGCCACCAAC
CCCCCAAATGCAAAAAGGGCC-
GGGTTAGCTTTCTCCGCGGAGAAATCGTTTTGTCGATTCTCAGAACTGTTCCAGCTCA
GGCTGTGCCTCATATATCGTTCAATTCGAATTCG-TGCACGT-
TCGCATTCGGCAGGACAACCAGAACCATGTTCAGTGACA---TC-----
TCTTCGAGAATATCGAGGGCCAA-
TCTTCGGGAGACCCGACCCGACCCCTTTACCACTCGCTATCCTTCGTAGA--------
AGGACA---C----CATAAGCGCGACATGCCGG--
GACGCGTGAGGGAATGCTGAGCCCGGACCACA-AGACAGTC------
AACAGGGTCCATCCAACT-
CCGCACTACAGGAGCGAGTGCTACCGCCGATTCGACATCCCTACGGAGGAAAAGAA
ATGCACCTGTGGGACCAGACGTACAGACTATGCCGCTGCACAATACTTCCGGTATAA
AGAAAATTTAAAAAGTTCAGACATC--CACTGGCCGGCCCGGCCTCCAAA---
CTAAGTTCAAAGGCA-----TTCTCAGA--TGAGCAGAATCCATCAAAAGTCCCG--
CACGCACTGGATA---
AAAGGAGTAGAAGCACAGACAGCCCCCCTAAAGGAACGGGGAGCAGAGG-
CGGATCTACGCGCCGAAAGCTAACTAGGAGCTGAGGAATTTTCGGATTACCCCGTG
ATGTATGTACAGT--AATTGACCACTCGATGCAACTGCACTTTTCCCATGA-CC--
ACTCCTCCTCAGCCA---

Aligned fasta file of *Cantharellus* Al-1 genes: G=golden, W=white
>G_Al-1_1379bp
CCATGTACACCGGGAATGTCACCATTCGATGCACCAGCAACTTATTCGTTGTTGCAA
TACACCGAATTCGCCCGAGGGATATGGTACCCTATCGGTGGTTTTGCCATCGTTCCC
ATCAAACTGGCCGAGATCGCTCAAAGATTCGGGGCTACCATCCGCCTGTCATCCCCC
GTCAAGTCACTGTTATTCTCCAATGACGGAAAGCGAGTATCAGGCGTTGTTACAGCG
TCCGGCGAATCCTTGGAAGCAGATATTGTGGTCTGTAATGCAGATCTCGTTTACGCT
TATGATAAACTTCTCCCATCTCCCCGGCCATCGTCTCTCGGCAATAATATCTTCGGTT
TCCTCGATGATCTCAATCCCCTTTCCTCTTCTAGGAGGAACGTGTTAAATAGAACTGC
ATCGTGCTCGACCATTTCCTTCTACTGGTCTCTTTCCCGACCTCTCCCACGCCGTCCT
AAACACGCACCAGCAGCATCAAATTCTTCTTCGCCTGCCAAGGGGAAGGATGACTA
CTACTTCTCCGCCCACAACATTTTCCTCGCAGAAGCTTACAAGCGATCTTTCGATCA
AATCTTCAAAGAGCATACACTTCCAACCGATCCAAGTTTCTATATAAATGTGCCTAG
TATGCTTGATGACACTGCTGCACCTGGTGGTTGCGATACCGTTGTGGTGTTGGTGCCT
GTTGGACATCTCACCCGGAGGGACTCGGTGGATTCTGAGGAACGAAATAGGGTGAA
TGCACTGGTGAACCGTGCAAGAATGCAAGTTTTGGAAACTATCAAAACTAGGATGG
GGATCGATCTTAGTAAGGATGGGTGGATTAAGCATGAGATGGTGAATAACCCAATC
GTATGTAAGTATAAGTTGACCTATAGTATAGCTGATACACTCACACCGTCGTTTCCC
CCTTTCCTTAGGGAAAAATAAGTTCAACCTCGATCGTGGTGCTGCTCTTGGACTCTC
ACTCGATCTCTTGTGAGTCATCTTGCAACATCGGCGGCATAAGCATCAGCAGCCCTG
CTCATCCTGATCAAACCCGTTCATTCTGCTAGCAATACACTCTGTTTCCGTCCTTGCA
CGCAGCATTCTCAGTTCAAGAATATGTACTTTGTCGGAGCTAGCACTCATCCAGGTA
AATACACACACACACCTTTCCCTCATGATCTTTACTTTTCTTTACTTTTTTTTTTTTTTT
TTCAAGGCACTGGCGTACCTGTCGTCCTTGCTGGAGCCAAGATAACCGCGGAACAA
ATCCTATCGAATCATCTTCCCCCTAATGAGACCTCAGGATTCAAATCCCTTCAAGGG
AATGGCCATGCCGTAGGGTACAACAAAGTATCCAGGCTTAAATCGAAGCGCACGGC
GTTCATAG
>W_Al-1_1375bp
CCATGTACACCGGGAATGTCACCATTCGATGCACCAGCAACTTATTCGTTGTTGCAA
TACACCGAATTCGCCCGAGGGATATGGTACCCTATCGGTGGTTTTGCCATCTTTCCC
ATCAAACTGGCCGAGATCGCTCAAAGATTCGGGGCTACCATCCGCCTGTCATCTCCC
GTCAAGTCACTGTTATTCTCCAATGACGGAAAGCGAGTATCAGGCGTTGTTACAGCG
TCCGGCGAATCCTTGGAAGCAGATATCGTGGTCTGTAATGCAGATCTCGTTTACGCT
TATGATAAACTTCTCCCATCTCCCCGGCCATCGTCTCTCGGCAATAATATCTTCGGTT
TCCTCGATGATCTCAATCCCCTTTCCTCTTCTAGGAGGAACGTGTTAAATAGAACTGC
ATCGTGCTCGACCATTTCCTTCTACTGGTCTCTTTCCCGACCTCTCCCACGCCGTCCT
AAACACGCACCAGCAGCATCAAATTCTTCTTCGCCTGCCAAGGGGAAGGATGACTA
CTACTTCTCCGCCCACAACATTTTCCTCGCAGAAGCTTACAAGCGATCTTTCGATCA
AATCTTTAAAGAGCATACACTTCCAACCGATCCAAGTTTTTATATAAATGTGCCTAG
TATGCTTGATGACACTGCTGCACCTGGTGGTTGCGATACCGTTGTGGTGTTGGTGCCT
GTTGGACATCTCACCCGGAGGGACTCGGTGGATTCTGAGGAACGAAATAGGGTGAA
TGCACTGGTGAACCGTGCAAGAATGCAAGTTTTGGAAACTATCAAAACTAGGATGG
GGATCGATCTTAGTAAGGATGGGTGGATTAAGCATGAGATGGTGAATAACCCAATC

GTATGTAAGTATAAGTTGACCTATAGTATAGCTGATACACTCACACCGTCGTTTCCC
CCTTTCCTTAGGGAAAAATAAGTTCAACCTCGATCGTGGTGCTGCTCTTGGACTCTC
ACTCGATCTCTTGTGAGTCATCTTGCAACATCGGCGGCATAAGCATCAGCAGCCCTG
CTCATCCTGATCAAACCCGTTCATTCTGCTAGCAATACACTCTGTTTCCGTCCTTGCA
CGCAGCATTCTCAGTTCAAGAATATGTACTTTGTCGGAGCTAGCACTCATCCAGGTA
AATACACACACACACCTTTCCCTCATGATCTTTACTTTTCTTTACTTTTTT---
TTTTTTTTCAAGGCACTGGCGTACCTGTCGTCCTTGCTGGAGCCAAGATAACCGCGG
AACAAATCCTATCGAATCATCTTCCCCCTAATGAGACCTCAGGATTCAAATCCCTTC
AAGGGAATGGCCATGCCGTAGGGTACAACAAAGTATCCAGGCTTAAATCGAAGCGC
ACGGCGTTCATAG

Aligned fasta file of *Cantharellus* Al-2 genes: G=golden, W=white
>G_Al-2_2249bp
GGTGTAGGTGACATGCTCACGTTGACATTGAGCCCATCTAATTCGGACGCTTAATAG
TCATCTCCGCTGGACTATACCTGCGTTTTCTGTGCTCTTGGCACTTTACTTCCCACTA
AGGACTTCTATCGACTTCTTCAGAGTTGGTTTCCTTGTCTTTATTGCTATCGCGTACA
CCATCCCATGGGATTCGTATCTTATCCGAAGCGACGTACGTCCTCGACTTTTGATCTG
ACACTGCGTTCTCTTGTTAATAAAACTTCACAAAAGGTTTGGTCATACCCGCCAGAC
GCGACGTTTAACGTGACGTTCTATTCCATTCCTGCTGAAGAACTCTTTTTTTTCGCCA
TCCAAACATGTTTCACTTCAGTTCTGTACCTCATCCTTTCGAAGCCCGTGTTACATCC
TGCCCTGATACCCACCCATGAACACCCCCCATCCACATCGCATACGGTGCTCCATCG
CCTTGGCACATTATGGTTTGCTGCTGGAACAGGTAGCGGTGCATGGTTATACTGGAG
AGGAGGCCAAGGAACGTATATGGGTCTCATACTCGTTTGGGCGTGTCCCATCCTTCA
GTTTATCTGGTGAATAATCCTCGCTTCTTGGTTTTTGGTTGAATGTATGAACCTATCG
TTTCGAAGGAGCGTAGCAGGACTGCATATCATCGCCATGCCCACCTTATCCTCGCTT
GGACCTATTCTCCTCTCGACTGCGTATATGTGGATTCTTGATGCGACAGCTCTTCAAC
GGGGCACTTGGGTCATCGAGTCTGGCACCAAATTTAATGCTCAGCGTGTGGGCCATC
TCGACGTAGAGTGCGTCGTATTTGCCTCCTTTCGCCACTCCATTTACGAAACGTCC
CGCTACCGTGATGTGTTTATTCAGAGAAGCAGTATTCTTTTTAATTACGAATTTCGTC
ATTGTGATTGGTCAAATCGCCATTGACAGGGCGTACAGGATCCTTACCATATACGCC
ATAGACTTACCCTCCACCGCGTCACACACCTTCTTGTCGGCACCATTCGCACTCATC
GCATCTTTCTCAGTGAGTTCGAGGTACCTGAATATCAAGGTGATCACCCACCGGCTC
ACCAACCTTCGGGACACCAGTTTGATCCTCAAAGGAAAAAGTAGAAGTTTCTGGAC
CGCAAGCAGTATATTCCAAGGTCCCTTAAGGATAGAATTGATCAATTTGTGAGCCTT
TCAGTTCTTAGTCATTCTGACTGATTAAGTTCTGGCGCCTAGGTATGCCTTTTGCCGA
GTTATCGACGATTTGATTGATGACGATGCCCGCACGAGACAGGAAGGACATGAGAT
TCTGAAGCATGTCCGCGCGTTCGTTGATCGATGGTACATCTCGGGGATACCTGCCGG
ACAGGAAAGCTCGCATTTCGATGATTTGCTGAAGCCGCTCAACTTGTCGACTGCCCA
ACGACGGGCCTTCTATGCGCTGCCCCGCACAAGCGCTGGATCGTCGGCCCGGAGCC
CAAAATACAGGCTGGTCGAGGACCCACTCCGTGAACCGCTATCGGAGCTCCTTGAC
GGCTTTGACTCGGACCTCATATTTAAGGAGTCAGAGAAGGCGGGTGCTCCACCAAAT
GAGATTCTGATTCATAACGACAAGGATTTGATTCGTACGTTCTTCTCGCTTGTTATCC
ACGTATTTGATCATGGAGCATTCCGTGTCTCTTAGTTTATGCGTACCGGGTTGCTGGT
ACAGTCGCCAAGATGTGTTGCCACATGATTTTCCACTATGCGCCCACTTCTTACAATT
CCCCTCGTCCTCGTGAGGAAATTCTGGACGCAGCTGTCAAAATGGGTATGGCCCTTC
AATTCGTAAATATGGCCCGCGATATTAGGGTGGACGAACGGGCTGGGAGGATGTAC

GTTCCGCGAGAATGGTGGATCGGCAACTTCTCCAAAGCTGAAAGCTCCGAGTCTGTT
GACCATCACGGATCGAACGCGAAGCCCGTGACTACACAATCGCCATATCATTTCCCA
CCTGACGAAGCTCGCATTCATCGACTTCACTTGCTTCAGATGGCGAAAGATTTCTTC
CTTACCTCACGACCAGCGATAGATCAGATGCCGACCATTTTCTCAGCACGCAAGGGC
TTGAGGGTCGCGGTCGAGAGTTACATGGAAATTGGAAGGCAGCTAGAGAAGAAGAT
TGGTGATACAAAATACACCTTAGACGCGGATGACATTTTTGCAATGACTCCTCTTCA
GACCCGTTGACAAGGCCTA
>W_Al-2_1752bp

CGGTGCATGGTTATACTGGAGAGGAGGCCAAGGAACGTATATGGGTCTCATACTCG
TTTGGGCGTGTCCCATCCTTCAGTTTATCTGGTGAATAATCCTCGCTTCTTGGTTTTG
GTTGAATGTATGAACCTATCGTTTCGAAGGAGCGTAGCAGGACTGCATATCATCGCC
ATGCCCACCTTATCCTCGCTTGGACCTATTCTCCTCTCGACTGCGTATATGTGGATTC
TTGATGCGACAGCTCTTCAACGGGGCACTTGGGTCATCGAGTCTGGCACCAAATTTA
ATGCTCAGCGTGTGGGCCATCTCGACGTAGAGTGCGTCGTATTTGCCTCCTTTTCGCC
ACTCCATTTTACGAAACGTCCCGCTACCGTGATGTGTTTATTCAGAGAAGCAGTATT
CTTTTTAATTACGAATTTCGTCATTGTGATTGGTCAAATCGCCATTGACAGGGCGTAC
AGGATCCTTACCATATACGCCATAGACTTACCCTCCACCGCGTCACACACCTTCTTG
TCGGCACCATTCGCACTCATCGCATCTTTCTCAGTGAGTTCGAGGTACCTGAATATC
AAGGTGATCACCCACCGGCTCACCAACCTTCGGGACACCAGTTTGATCCTCAAAGG
AAAAAGTAGAAGTTTCTGGACCGCAAGCAGTATATTCCAAGGTCCCTTAAGGATAG
AATTGATCAATTTGTGAGCCTTTCAGTTCTTAGTCATTCTGACTGATTAAGTTCTGGC
GCCTAGGTATGCCTTTTGCCGAGTTATCGACGATTTGATTGATGACGATGCCCGCAC
GAGACAGGAAGGTCATGAGATTCTGAAGCATGTCCGCGCGTTCGTTGATCGATGGT
ACATCTCGGGGATACCTGCCGGACAGGAAAGCTCGCATTTCGATGATTTGCTGAAGC
CGCTCAACTTGTCGACTGCCCAACGACGGGCCTTCTATGCGCTGCCCCGCACAAGCG
CTGGATCGTCGGCCCGGAGCCCAAAATACAGGCTGGTCGAGGACCCACTCCGTGAA
CCGCTATCGGAGCTCCTTGACGGCTTTGACTCGGACCTCATATTTAAGGAGTCAGAG
AAGGCGGGTGCTCCACCAAATGAGATTCTGATTCATAACGACAAGGATTTGATTCGT
ACGTTCTTCTCGCTTGTTATCCACGTATTTGATCATGGAGCATTCCGTGTCTCTTAGT
TTATGCGTACCGGGTTGCTGGTACAGTCGCCAAGATGTGTTGCCACATGATTTTCCA
CTATGCGCCCACTTCTTACAATTCCCCTCGTCCTCGTGAGGAAATTCTGGACGCAGC
TGTCAAAATGGGTATGGCCCTTCAATTCGTAAATATGGCCCGCGATATTAGGGTGGA
CGAACGGGCTGGGAGGATGTACGTTCCGCGAGAATGGTGGATCGGCAACTTCTCCA
AAGCTGAAAGCTCCGAGTCTGTTGACCATCACGGATCGAACGCGAAGCCCGTGACT
ACACAATCGCCATATCATTTCCCACCTGACGAAGCTCGCATTCATCGACTTCACTTG
CTTCAGATGGCGAAAGATTTCTTCCTTACCTCACGACCAGCGATAGATCAGATGCCG
ACCATTTTCTCAGCACACAAGGGCTTGAGGGTCGCGGTCGAGAGTTACATGGAAATT
GGAAGGCAGCTAGAGAAGAAGATTGGTGATACAAAATACACCTTAGACGCGGATGA
CATTTTTGCAATGACTCCTCTTCAGACCCGTTGACAAGGCCTA

Fasta file of deduced amino acid sequences from *Cantharellus* Al-1 genes: G=golden, W=white
>G_Al-1
GMSPFDAPATYSLLQYTEFARGIWYPIGGFAIVPIKLAEIAQRFGATIRLSSPVKSLLFSND
GKRVSGVVTASGESLEADIVVCNADLVYAYDKLLPSPRPSSLGNNIFGFLDDLNPLSSSR
RNVLNRTASCSTISFYWSLSRPLPRRPKHAPAASNSSSPAKGKDDYYFSAHNIFLAEAYK
RSFDQIFKEHTLPTDPSFYINVPSMLDDTAAPGGCDTVVVLVPVGHLTRRDSVDSEERNR
VNALVNRARMQVLETIKTRMGIDLSKDGWIKHEMVNNPIVCKYKLTYTSAALLILIKPV
HSASNTLCFRPCTQHSQFKNMYFVGASTHPGKYTHTPFPHDLYFSLLFFFFFQGTGVPVV
LAGAKITAEQILSNHLPPNETSGFKSLQGNGHAVGYNKVSRLKSKRTAFI
>W_Al-1
GMSPFDAPATYSLLQYTEFARGIWYPIGGFAIFPIKLAEIAQRFGATIRLSSPVKSLLFSND
GKRVSGVVTASGESLEADIVVCNADLVYAYDKLLPSPRPSSLGNNIFGFLDDLNPLSSSR
RNVLNRTASCSTISFYWSLSRPLPRRPKHAPAASNSSSPAKGKDDYYFSAHNIFLAEAYK
RSFDQIFKEHTLPTDPSFYINVPSMLDDTAAPGGCDTVVVLVPVGHLTRRDSVDSEERNR
VNALVNRARMQVLETIKTRMGIDLSKDGWIKHEMVNNPIVCKYKLTYTSAALLILIKPV
HSASNTLCFRPCTQHSQFKNMYFVGASTHPGKYTHTPFPHDLYFSLLFFFFQGTGVPVVL
AGAKITAEQILSNHLPPNETSGFKSLQGNGHAVGYNKVSRLKSKRTAFI

Fasta file of deduced amino acid sequences from *Cantharellus* Al-2 genes: G=golden, W=white
>G_Al-2
IRTLNSHLRWTIPAFSVLLALYFPLRTSIDFFRVGFLVFIAIAYTIPWDSYLIRSDVWSYPP
DATFNVTFYSIPAEELFFFAIQTCFTSVLYLILSKPVLHPALIPTHEHPPSTSHTVLHRLGTL
WFAAGTGSGAWLYWRGGQGTYMGLILVWACPILQFIWSVAGLHIIAMPTLSSLGPILLS
TAYMWILDATALQRGTWVIESGTKFNAQRVGHLDVECVVFASFSPLHFTKRPATVMCL
FREAVFFLITNFVIVIGQIAIDRAYRILTIYAIDLPSTASHTFLSAPFALIASFSVSSRYLNIKV
ITHRLTNLRDTSLILKGKSRSFWTASSIFQGPLRIELINLYAFCRVIDDLIDDDARTRQEGH
EILKHVRAFVDRWYISGIPAGQESSHFDDLLKPLNLSTAQRRAFYALPRTSAGSSARSPK
YRLVEDPLREPLSELLDGFDSDLIFKESEKAGAPPNEILIHNDKDLILYAYRVAGTVAKM
CCHMIFHYAPTSYNSPRPREEILDAAVKMGMALQFVNMARDIRVDERAGRMYVPREW
WIGNFSKAESSESVDHHGSNAKPVTTQSPYHFPPDEARIHRLHLLQMAKDFFLTSRPAID
QMPTIFSARKGLRVAVESYMEIGRQLEKKIGDTKYTLDADDIFAMTPLQTR
>W_Al-2
GAWLYWRGGQGTYMGLILVWACPILQFIWSVAGLHIIAMPTLSSLGPILLSTAYMWILD
ATALQRGTWVIESGTKFNAQRVGHLDVECVVFASFSPLHFTKRPATVMCLFREAVFFLI
TNFVIVIGQIAIDRAYRILTIYAIDLPSTASHTFLSAPFALIASFSVSSRYLNIKVITHRLTNL
RDTSLILKGKSRSFWTASSIFQGPLRIELINLYAFCRVIDDLIDDDARTRQEGHEILKHVRA
FVDRWYISGIPAGQESSHFDDLLKPLNLSTAQRRAFYALPRTSAGSSARSPKYRLVEDPL
REPLSELLDGFDSDLIFKESEKAGAPPNEILIHNDKDLILYAYRVAGTVAKMCCHMIFHY
APTSYNSPRPREEILDAAVKMGMALQFVNMARDIRVDERAGRMYVPREWWIGNFSKA
ESSESVDHHGSNAKPVTTQSPYHFPPDEARIHRLHLLQMAKDFFLTSRPAIDQMPTIFSA
HKGLRVAVESYMEIGRQLEKKIGDTKYTLDADDIFAMTPLQTR
